# Supplementary figures and images for: Endomembrane targeting of human OAS1 p46 augments antiviral activity
Source: eLife. 2021 Aug 3;10:e71047. doi: 10.7554/eLife.71047 (PMC8357416; doi:10.7554/eLife.71047)

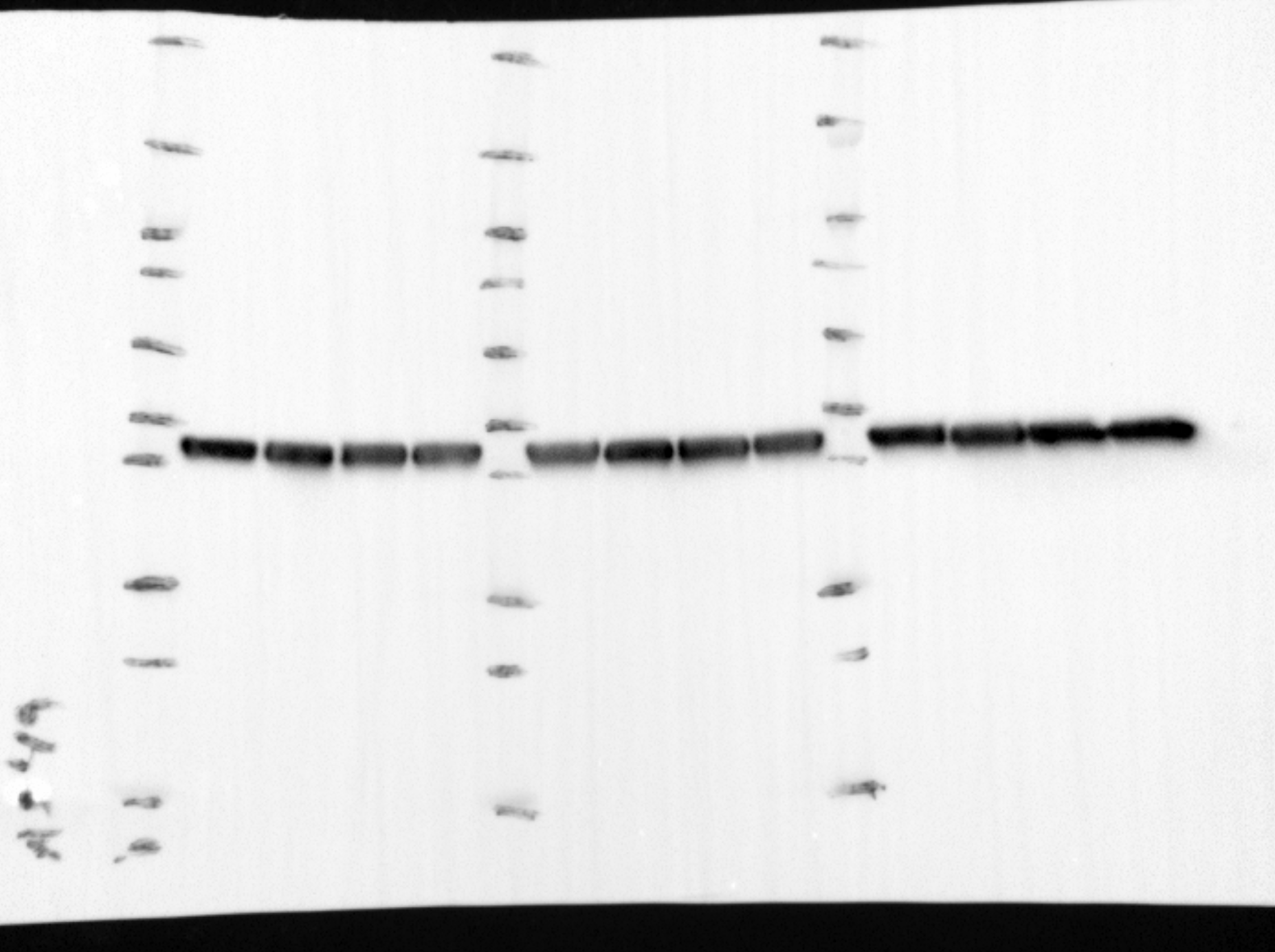

Supplement: Figure 1—source data 1. [file elife-71047-fig1-data1.zip › Figure 1 - Figure Supplement 2B IFNB A549 Actin.tif]

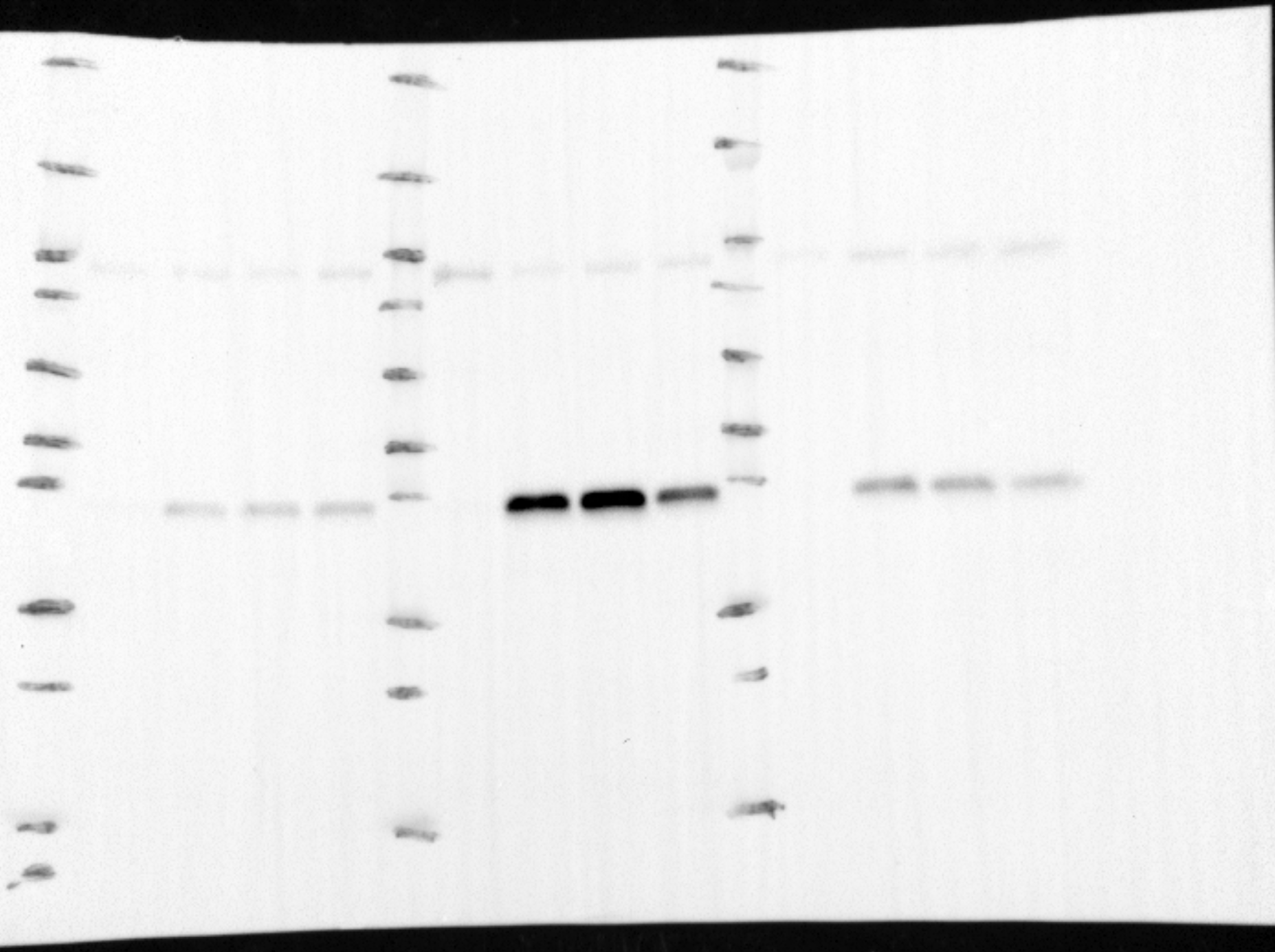

Supplement: Figure 1—source data 1. [file elife-71047-fig1-data1.zip › Figure 1 - Figure Supplement 2B IFNB A549 OAS1.tif]

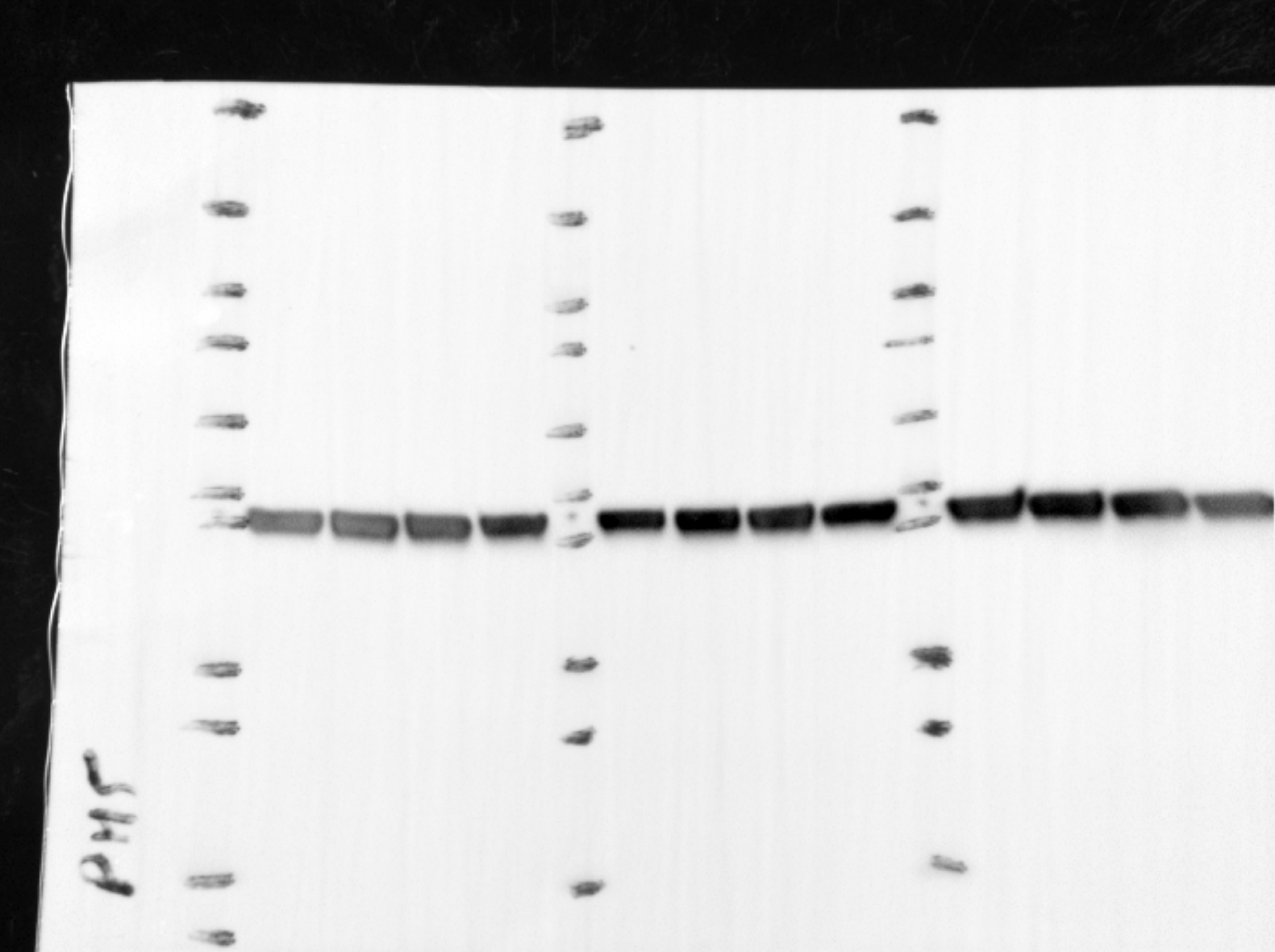

Supplement: Figure 1—source data 1. [file elife-71047-fig1-data1.zip › Figure 1 - Figure Supplement 2B IFNB PH5CH8 Actin.tif]

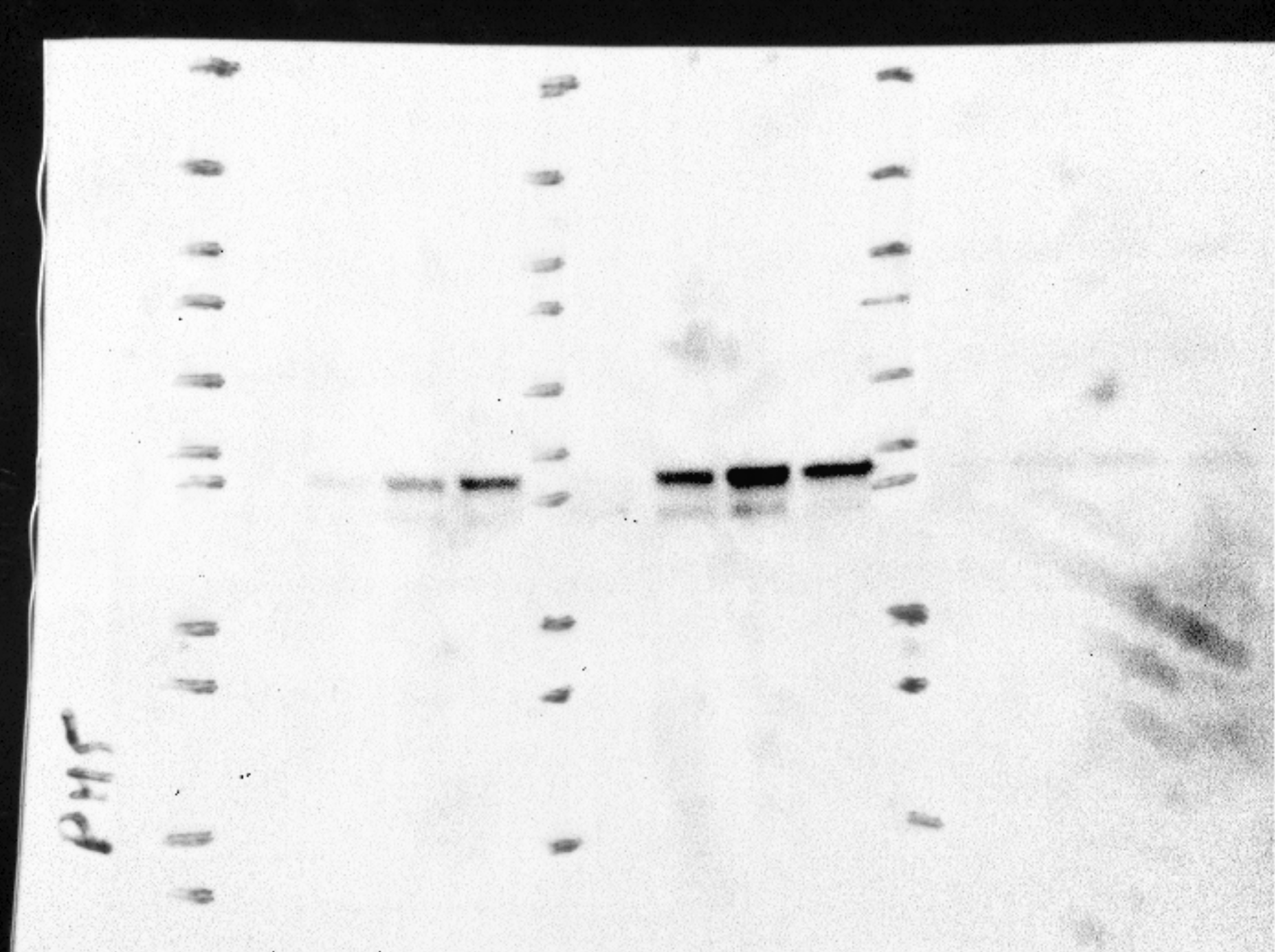

Supplement: Figure 1—source data 1. [file elife-71047-fig1-data1.zip › Figure 1 - Figure Supplement 2B IFNB PH5CH8 OAS1.tif]

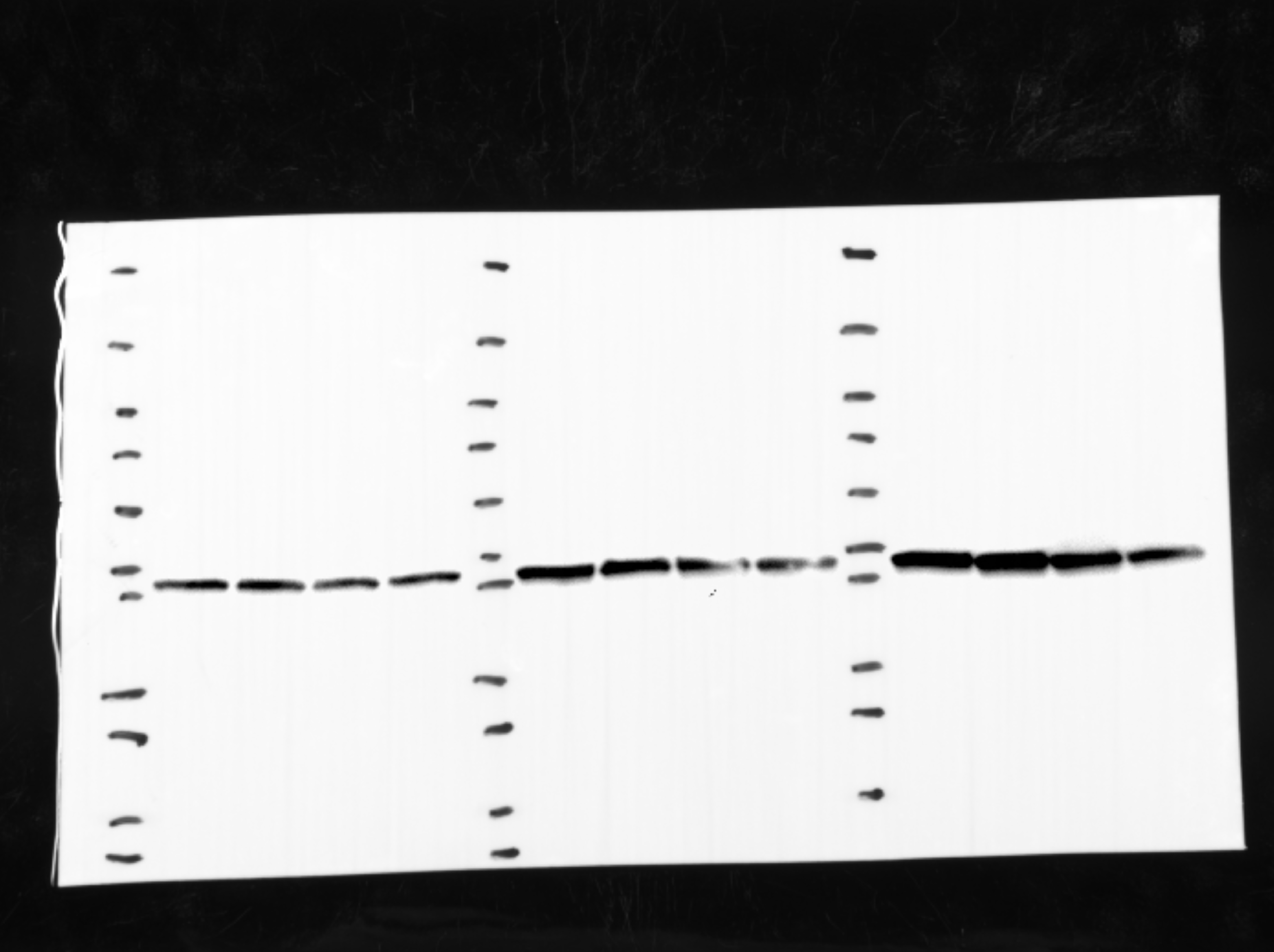

Supplement: Figure 1—source data 1. [file elife-71047-fig1-data1.zip › Figure 1 - Figure Supplement 2B SeV Actin.tif]

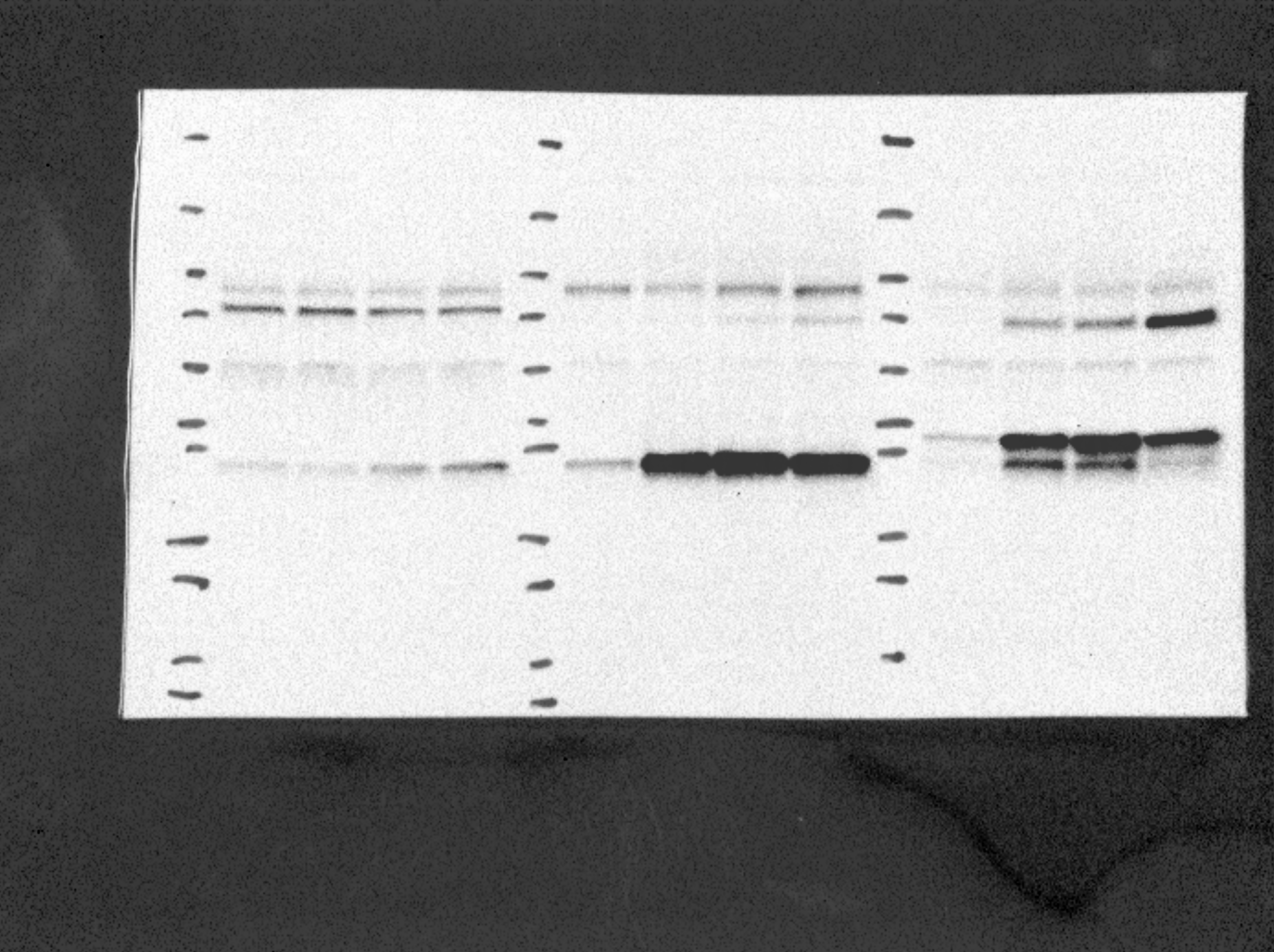

Supplement: Figure 1—source data 1. [file elife-71047-fig1-data1.zip › Figure 1 - Figure Supplement 2B SeV OAS1.tif]

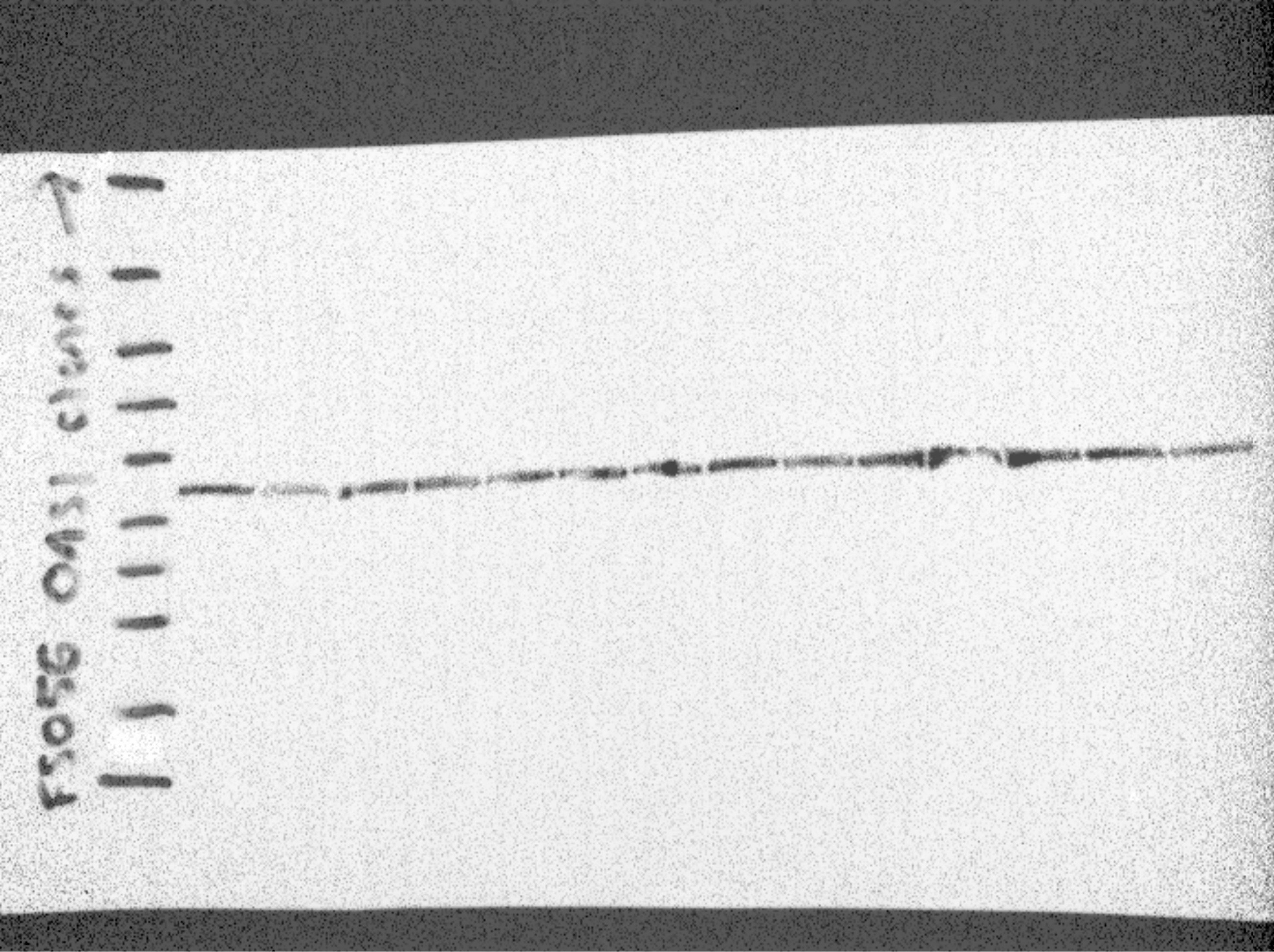

Supplement: Figure 1—source data 1. [file elife-71047-fig1-data1.zip › Figure 1 - Figure Supplement 2C Actin.tif]

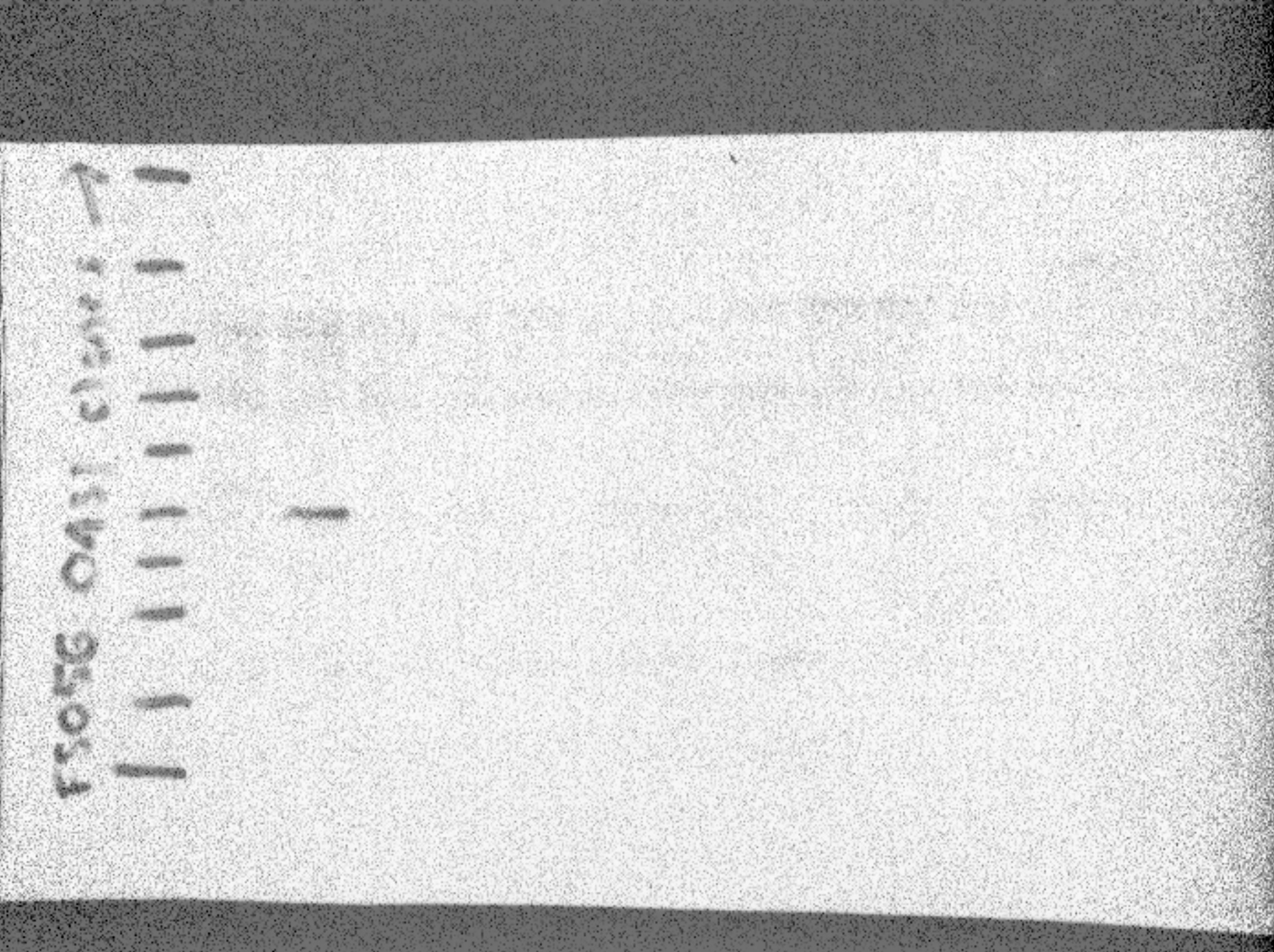

Supplement: Figure 1—source data 1. [file elife-71047-fig1-data1.zip › Figure 1 - Figure Supplement 2C OAS1.tif]

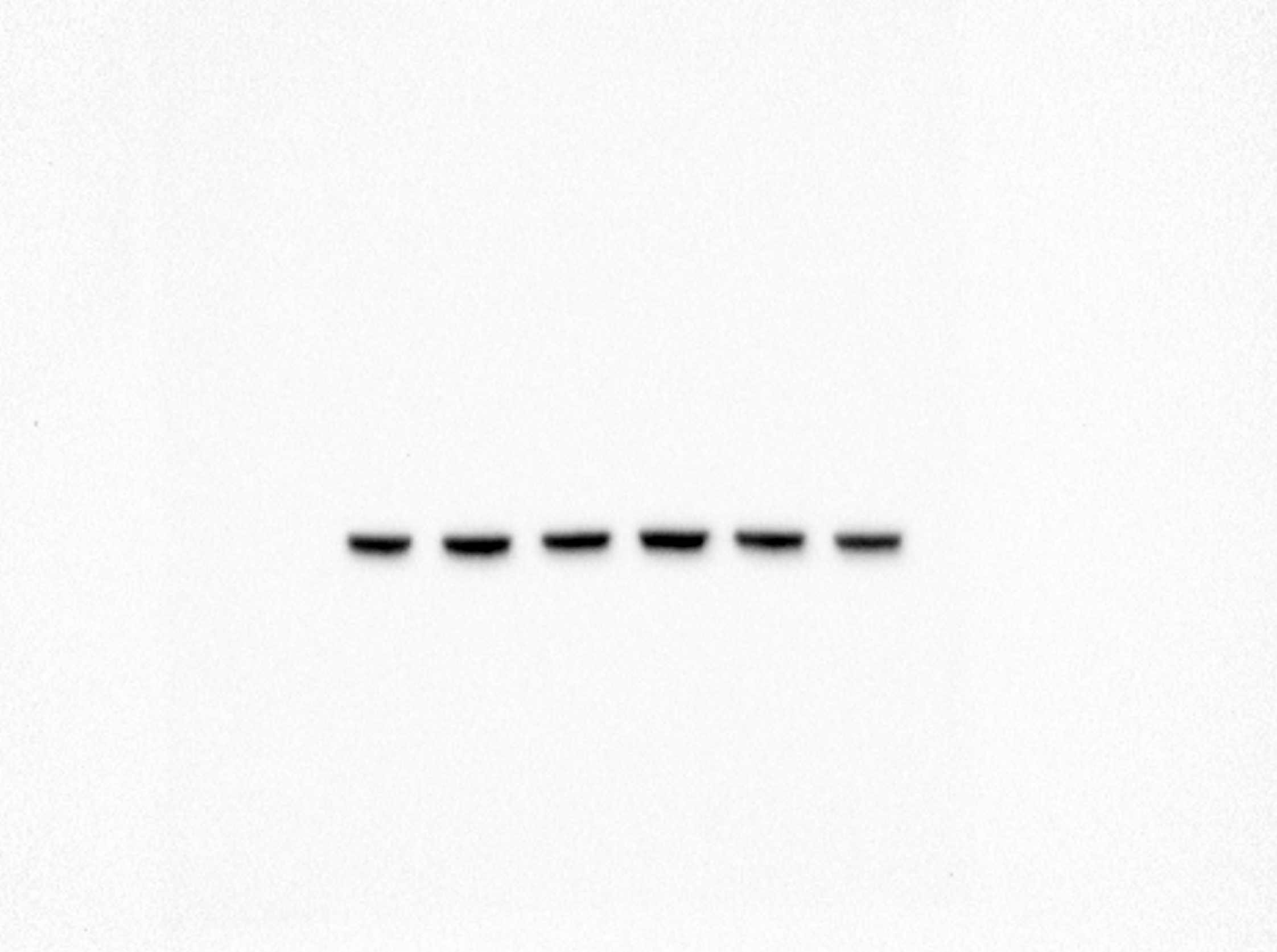

Supplement: Figure 1—source data 1. [file elife-71047-fig1-data1.zip › Figure 1 - Figure Supplement 2F Actin.tif]

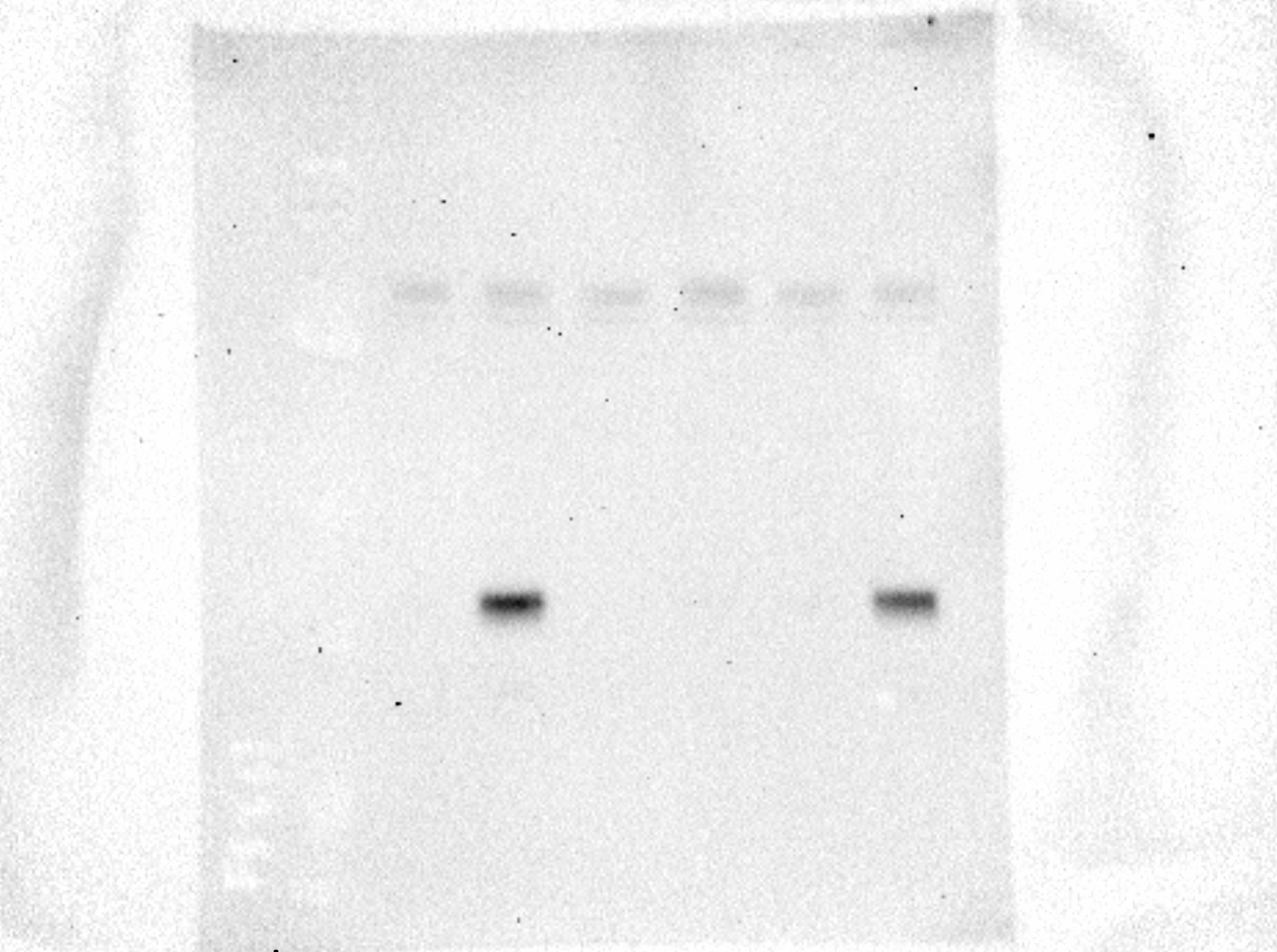

Supplement: Figure 1—source data 1. [file elife-71047-fig1-data1.zip › Figure 1 - Figure Supplement 2F OAS1.tif]

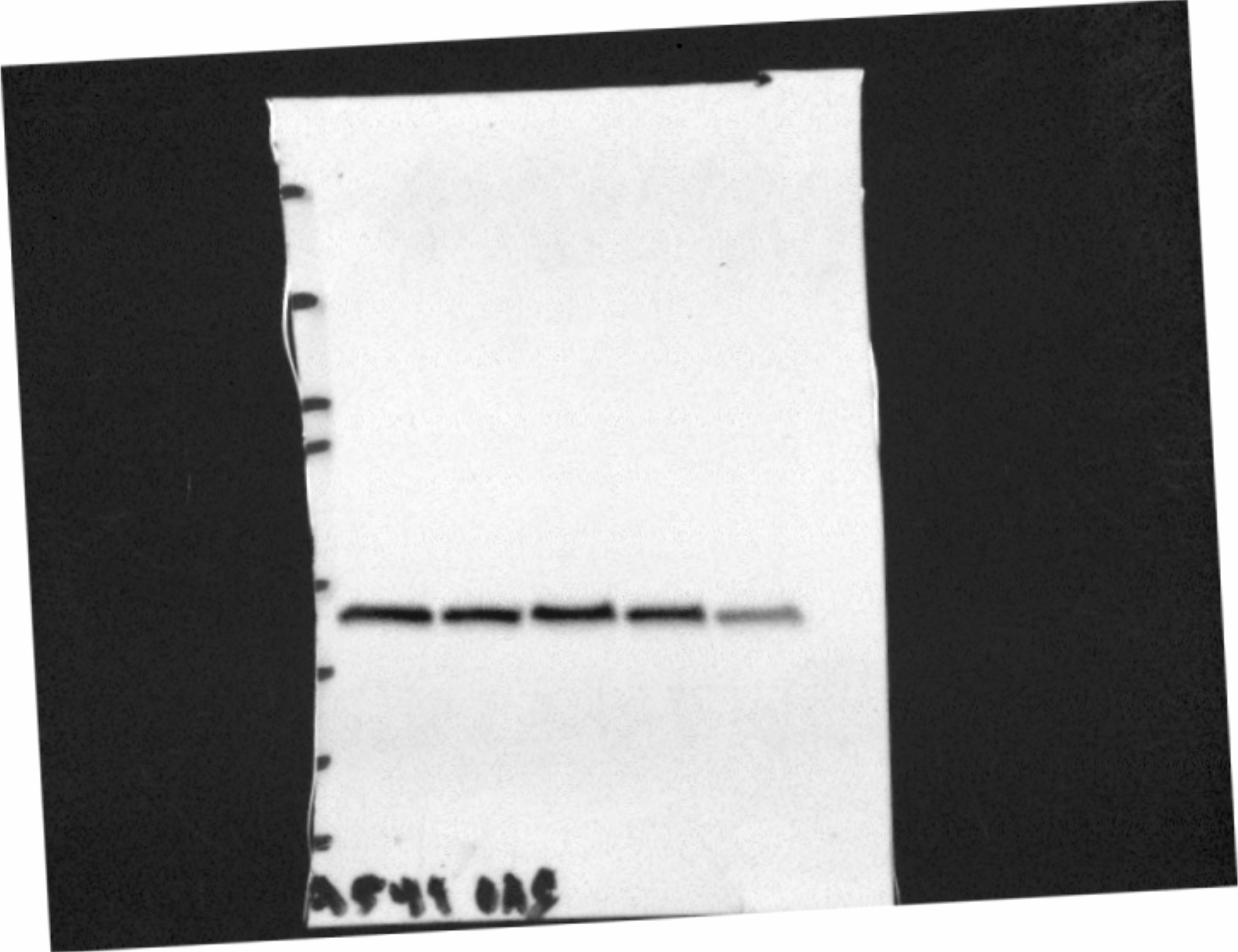

Supplement: Figure 1—source data 1. [file elife-71047-fig1-data1.zip › Figure 1 - Figure Supplement 2G Actin.tif]

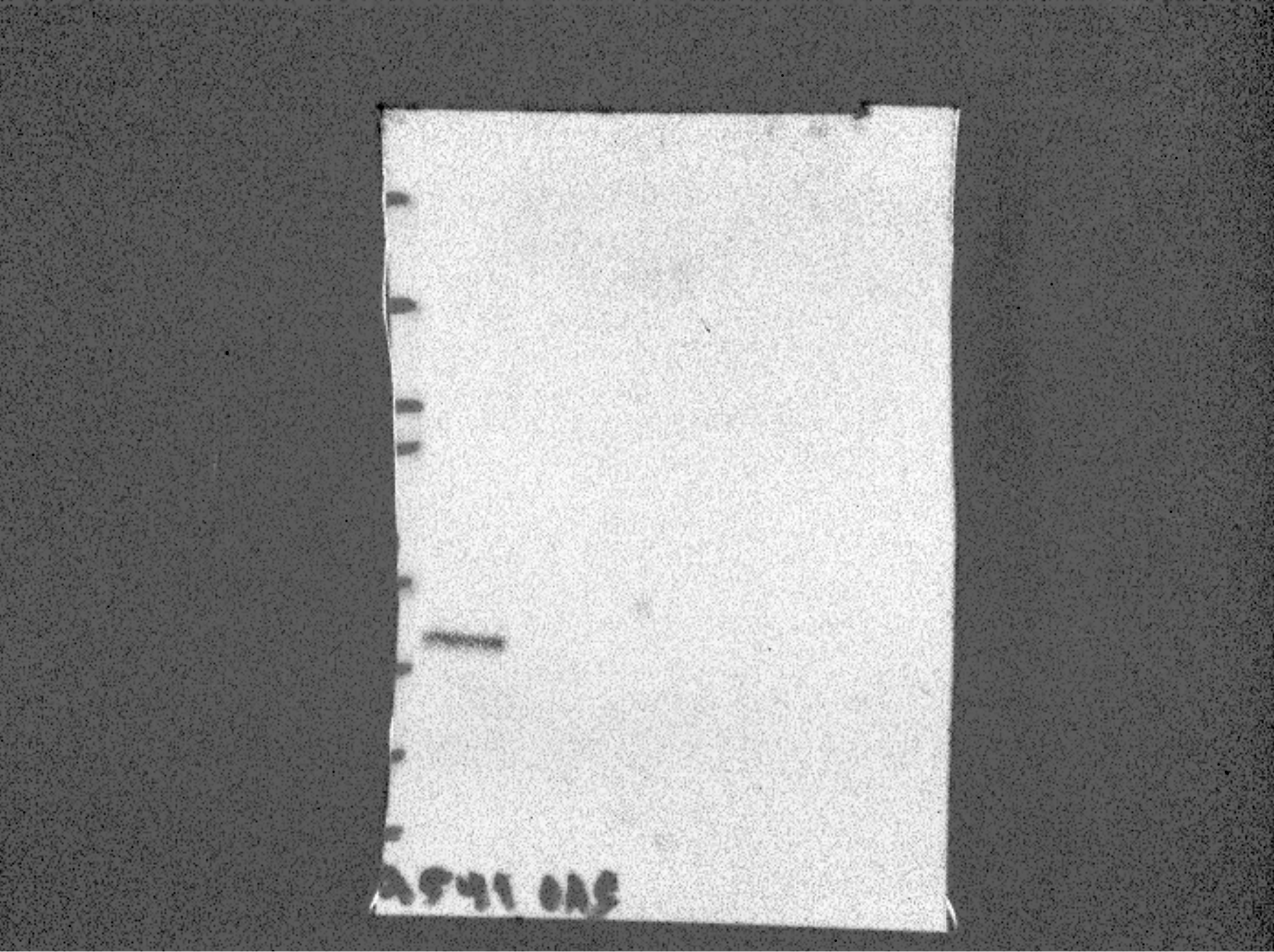

Supplement: Figure 1—source data 1. [file elife-71047-fig1-data1.zip › Figure 1 - Figure Supplement 2G OAS1.tif]

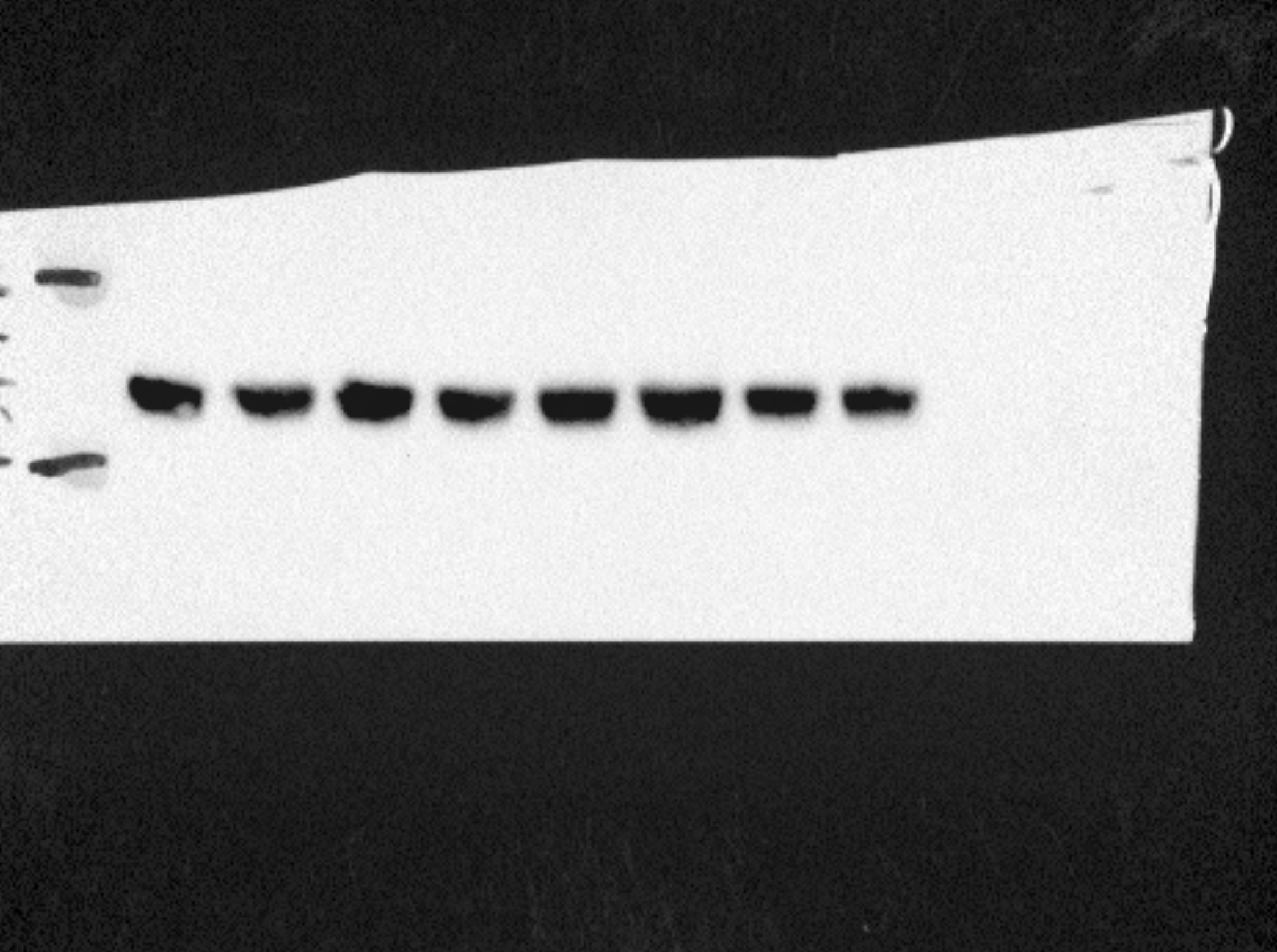

Supplement: Figure 1—source data 1. [file elife-71047-fig1-data1.zip › Figure 1B Actin.tif]

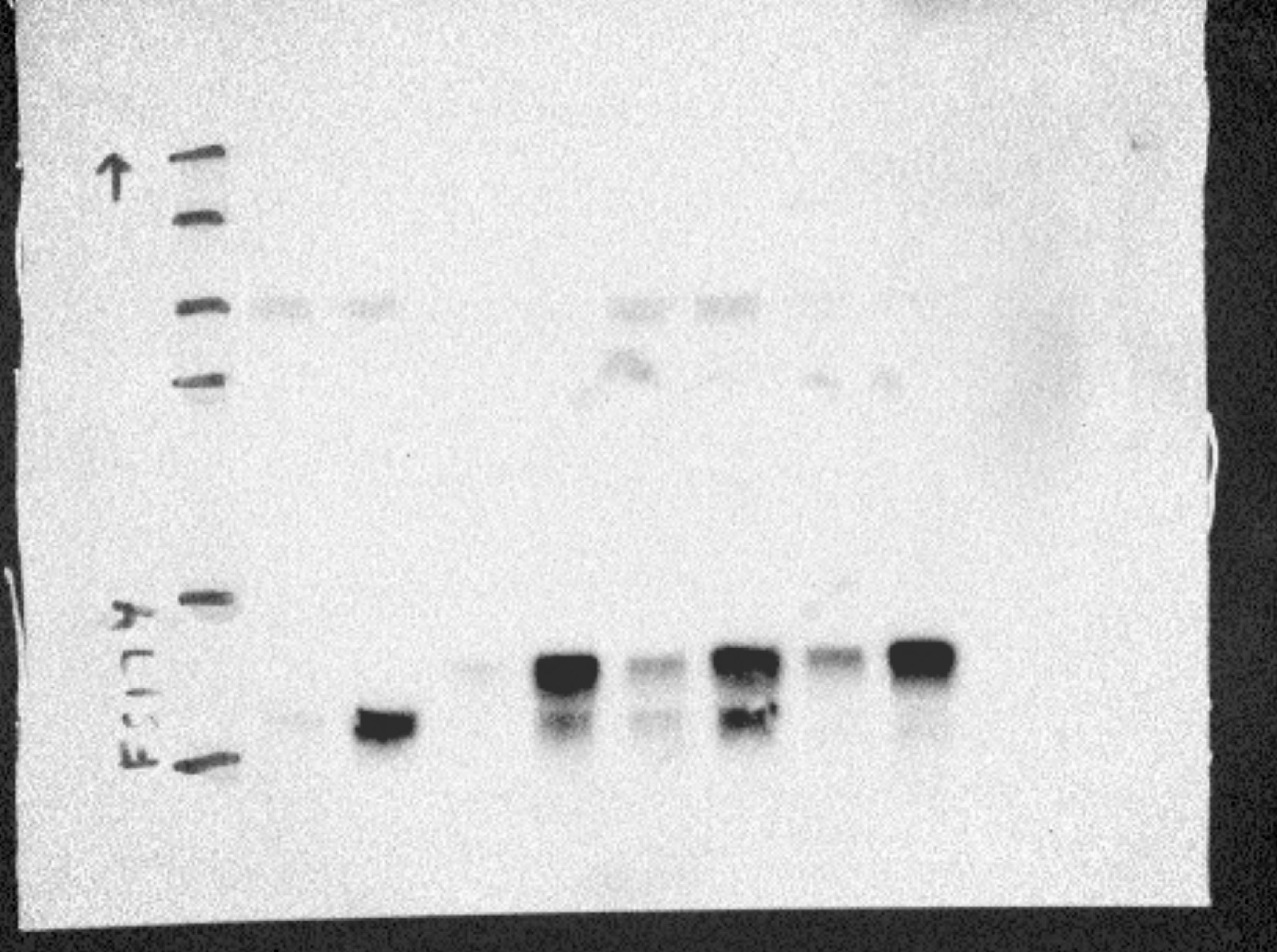

Supplement: Figure 1—source data 1. [file elife-71047-fig1-data1.zip › Figure 1B OAS1.tif]

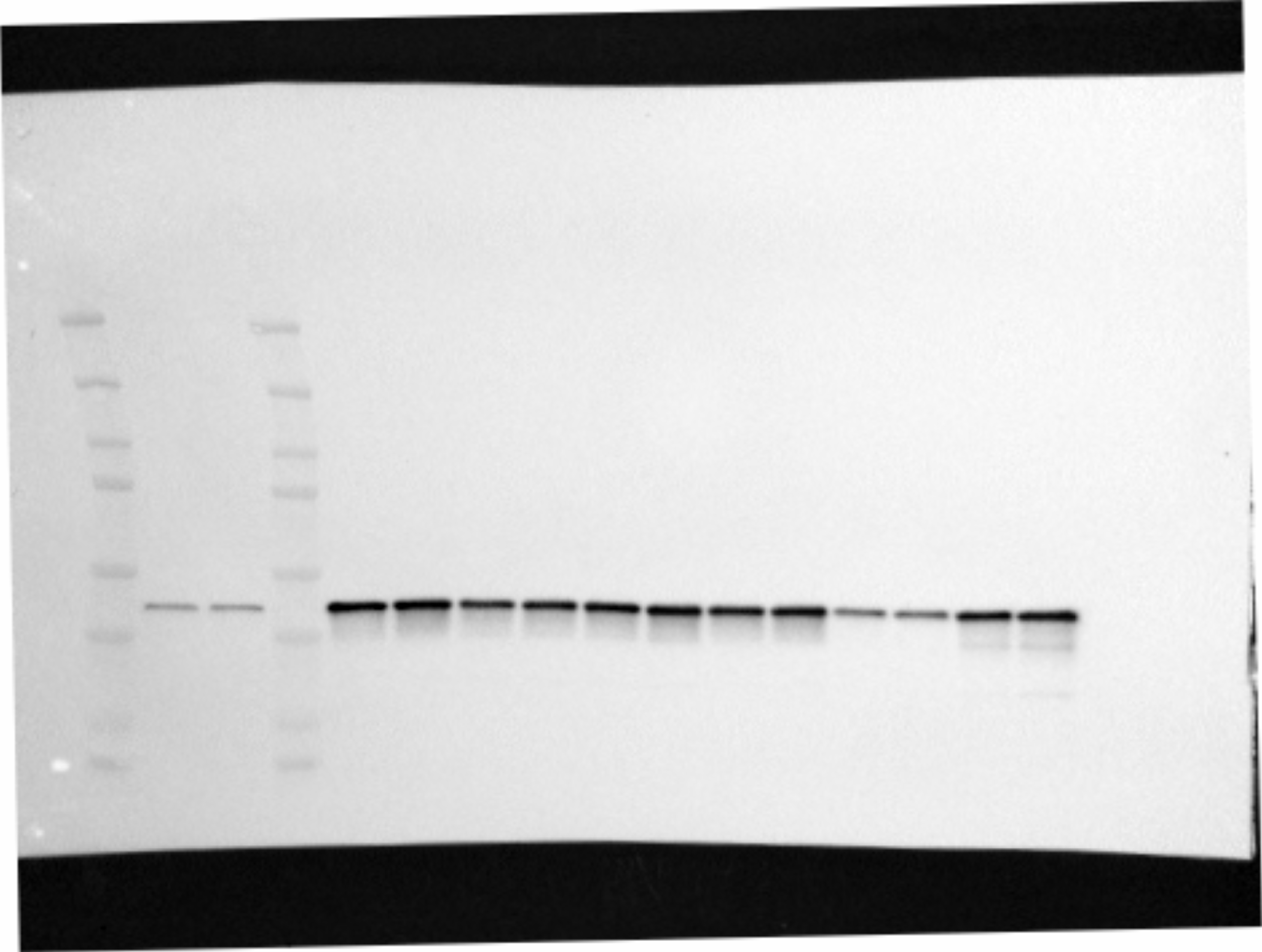

Supplement: Figure 1—source data 1. [file elife-71047-fig1-data1.zip › Figure 1C left Actin.tif]

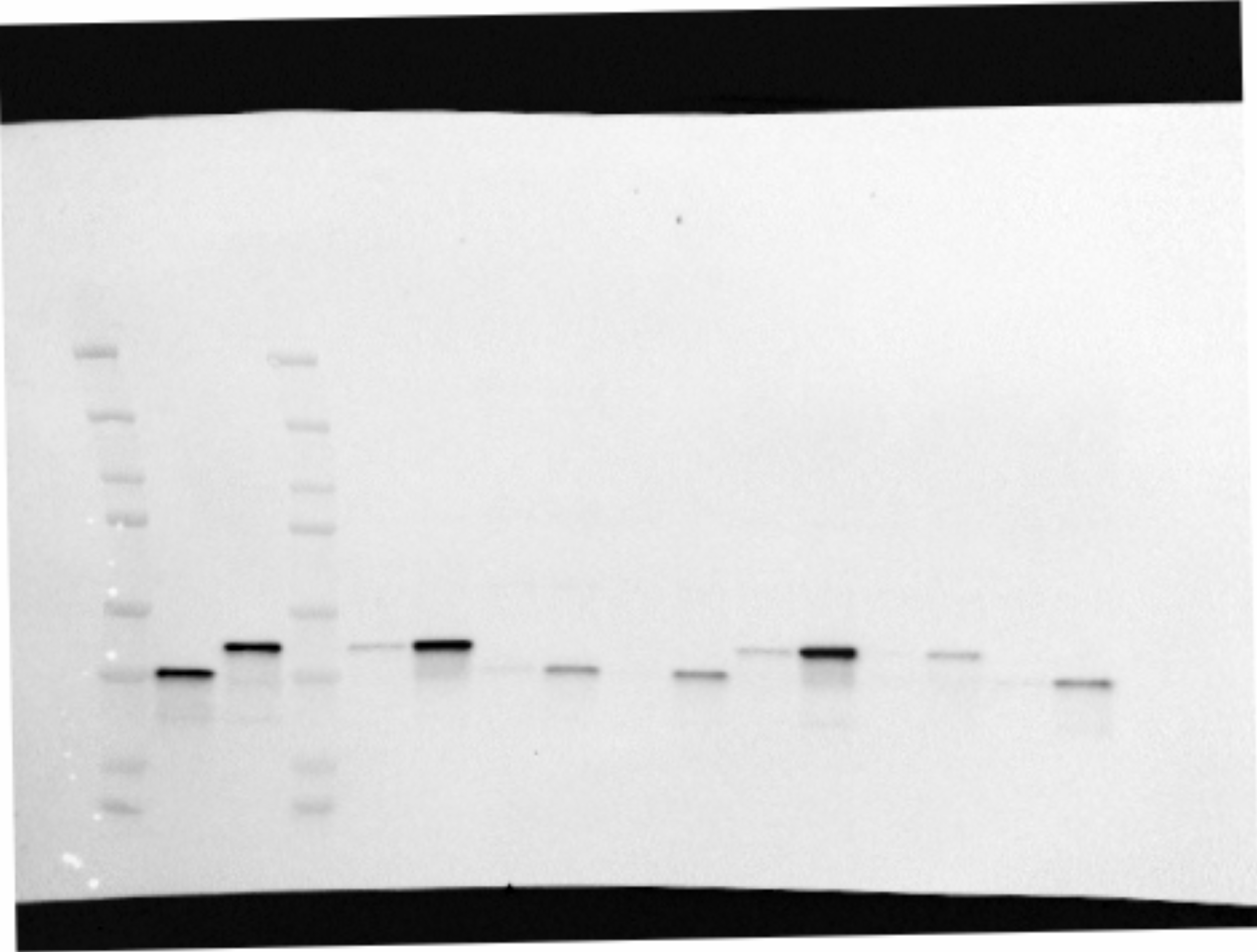

Supplement: Figure 1—source data 1. [file elife-71047-fig1-data1.zip › Figure 1C left OAS1.tif]

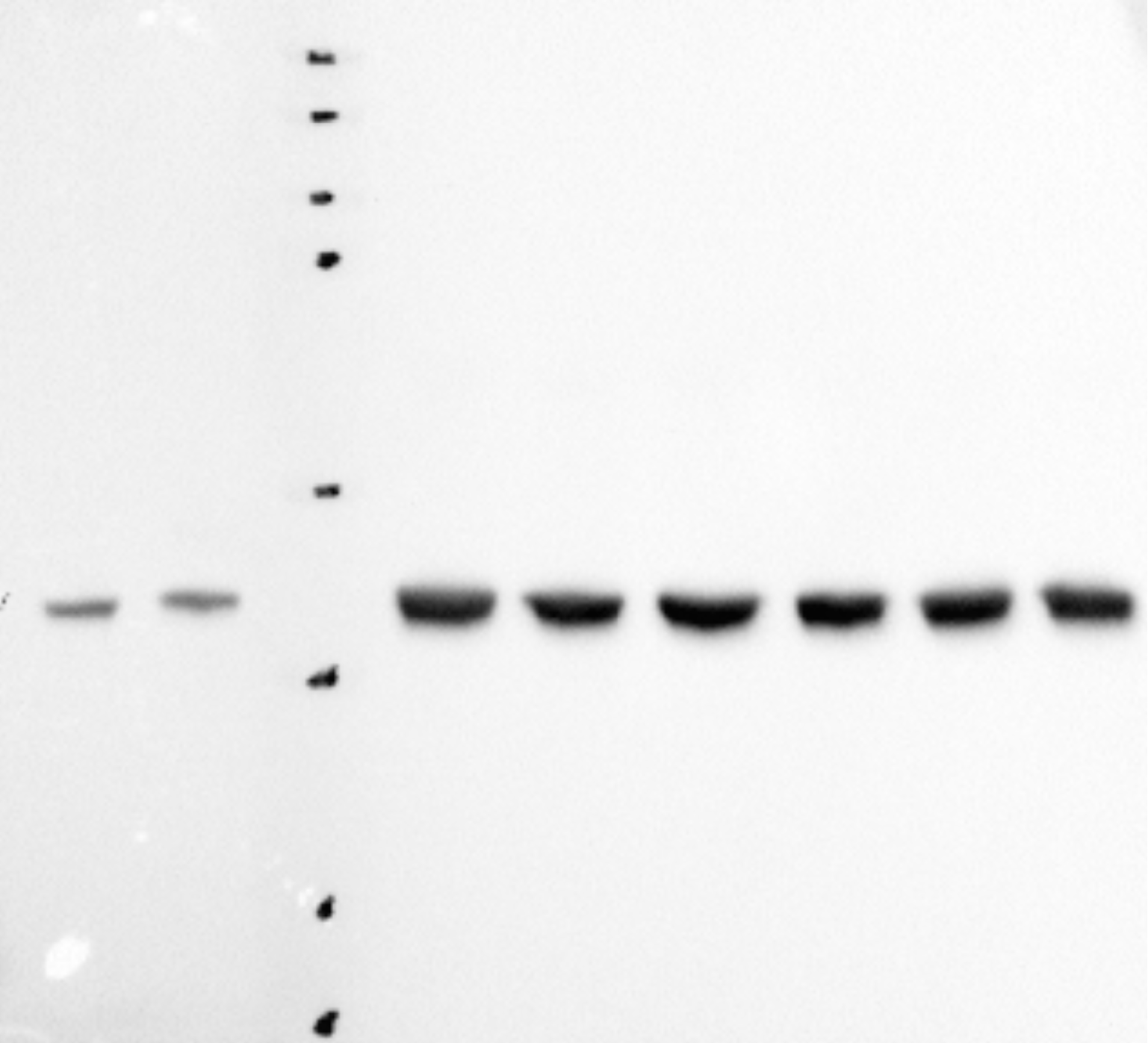

Supplement: Figure 1—source data 1. [file elife-71047-fig1-data1.zip › Figure 1C right Actin.tif]

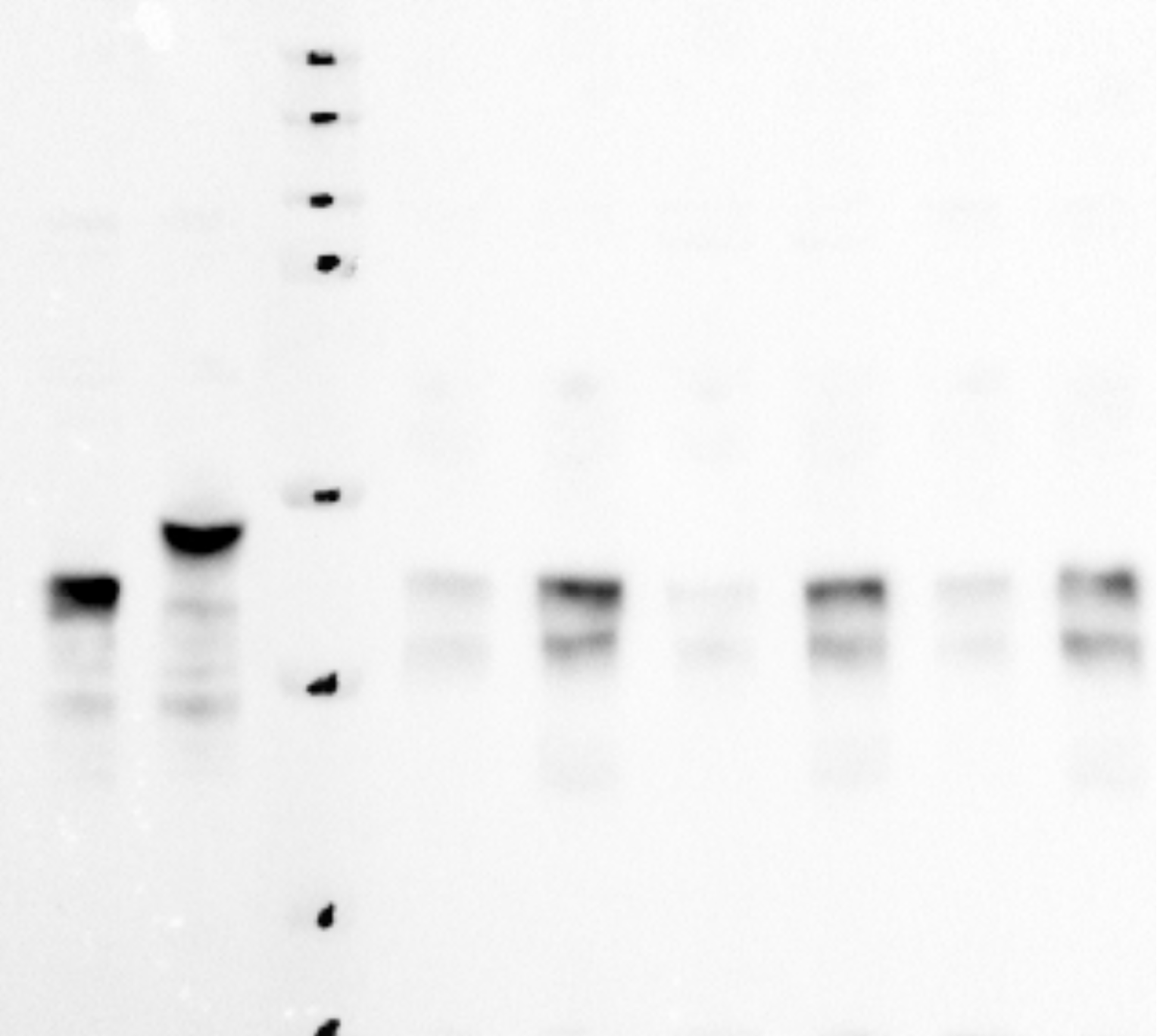

Supplement: Figure 1—source data 1. [file elife-71047-fig1-data1.zip › Figure 1C right OAS1.tif]

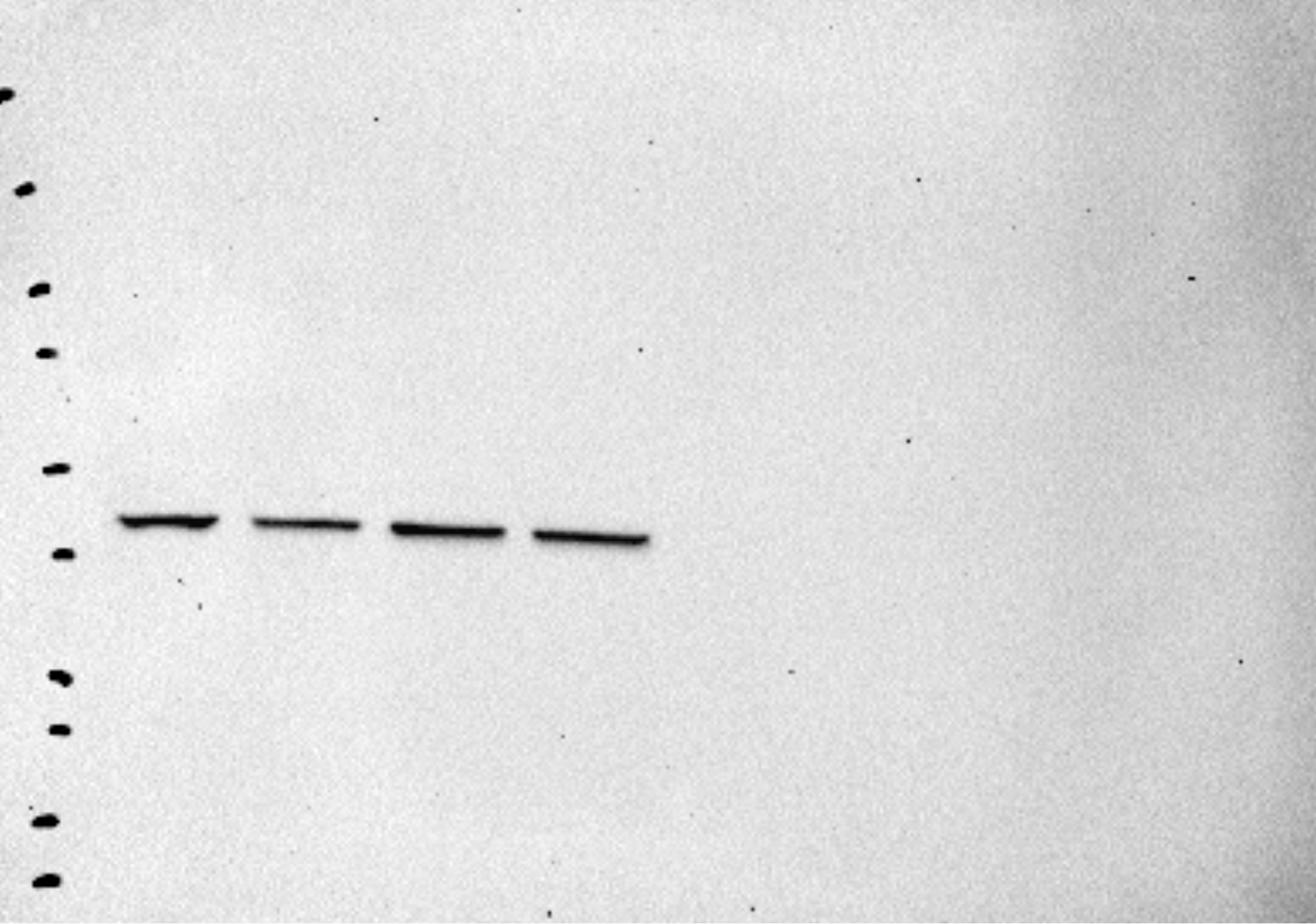

Supplement: Figure 1—source data 1. [file elife-71047-fig1-data1.zip › Figure 1D Actin.tif]

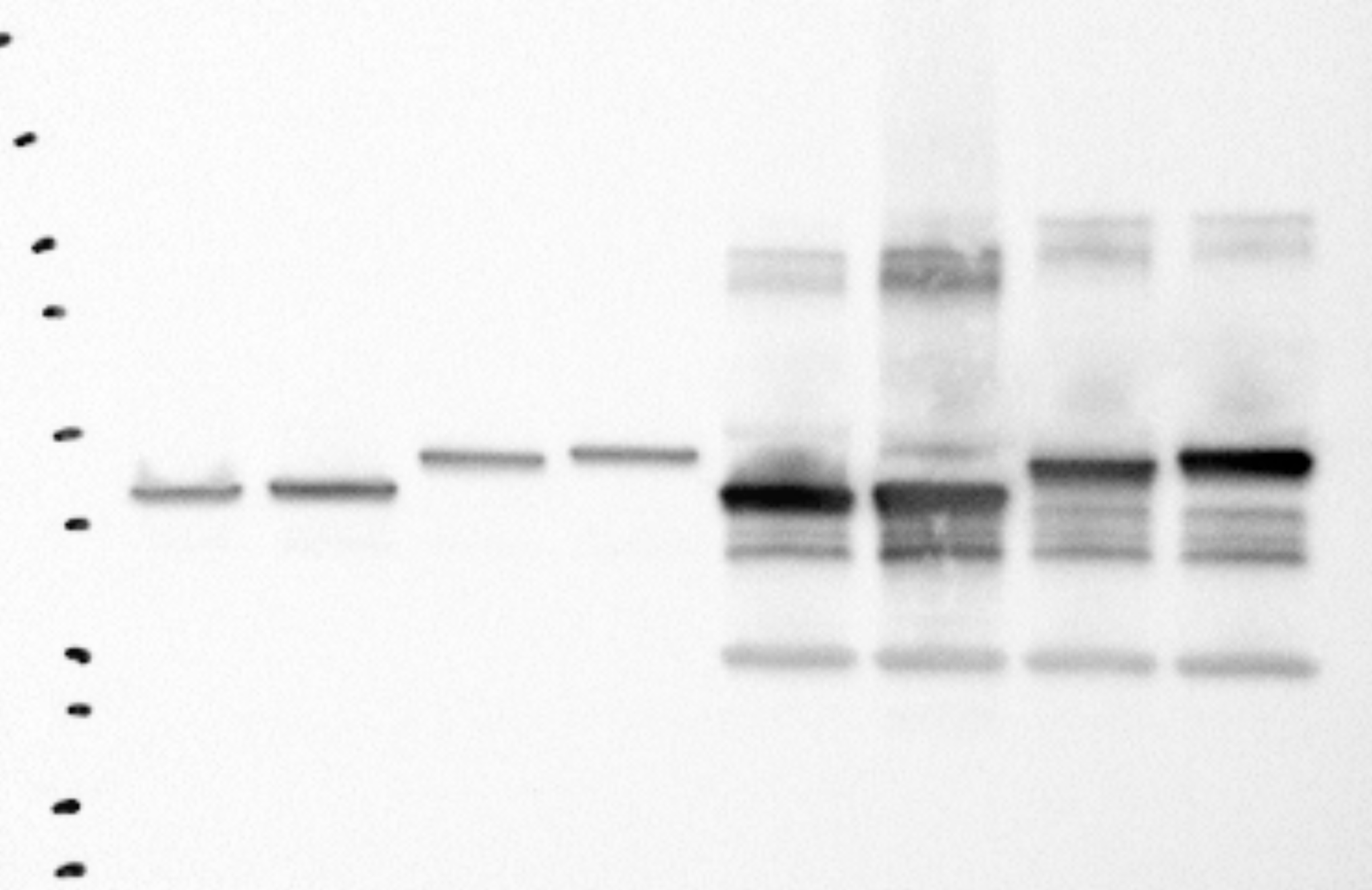

Supplement: Figure 1—source data 1. [file elife-71047-fig1-data1.zip › Figure 1D FLAG.tif]

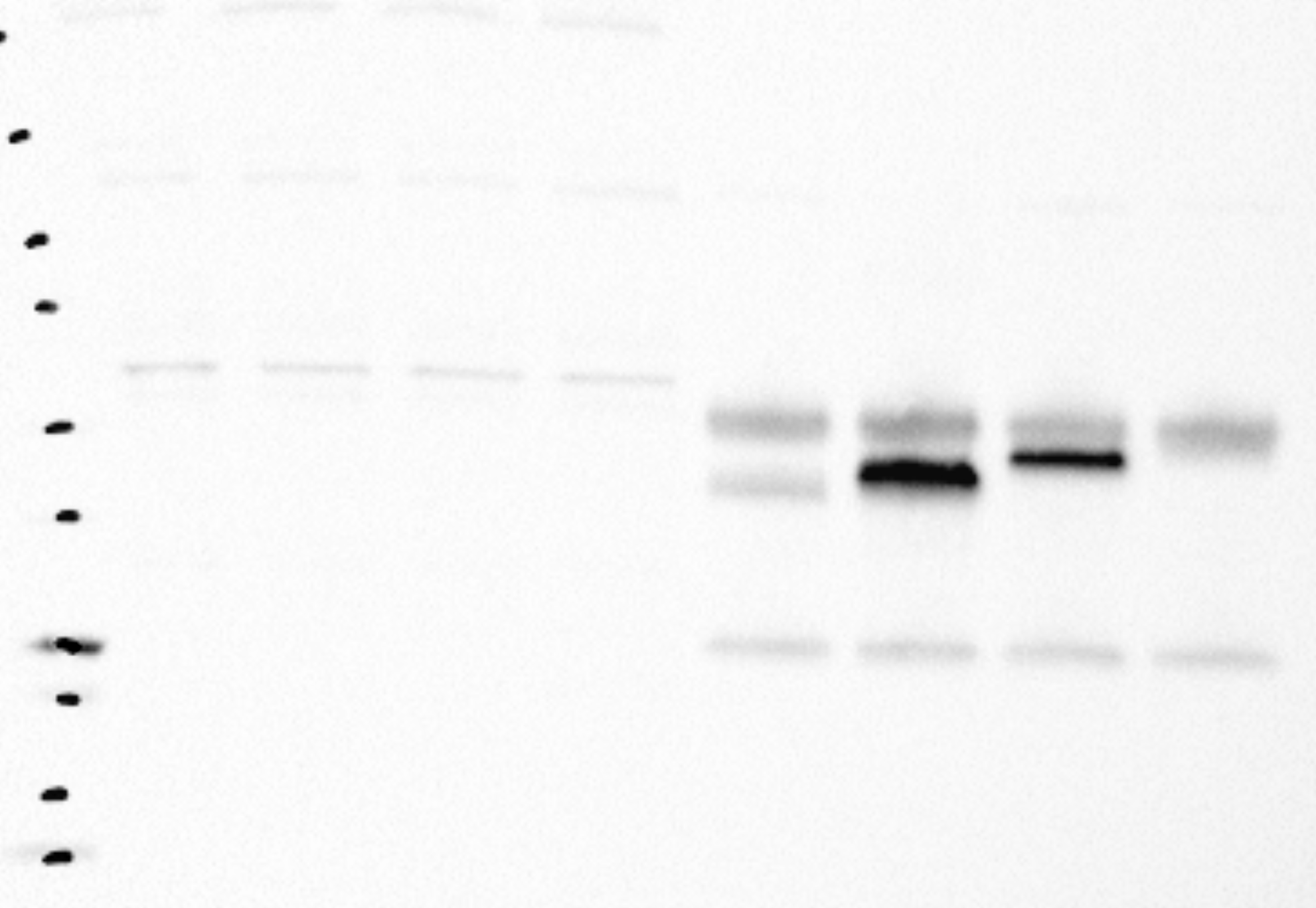

Supplement: Figure 1—source data 1. [file elife-71047-fig1-data1.zip › Figure 1D Strep-HRP.tif]

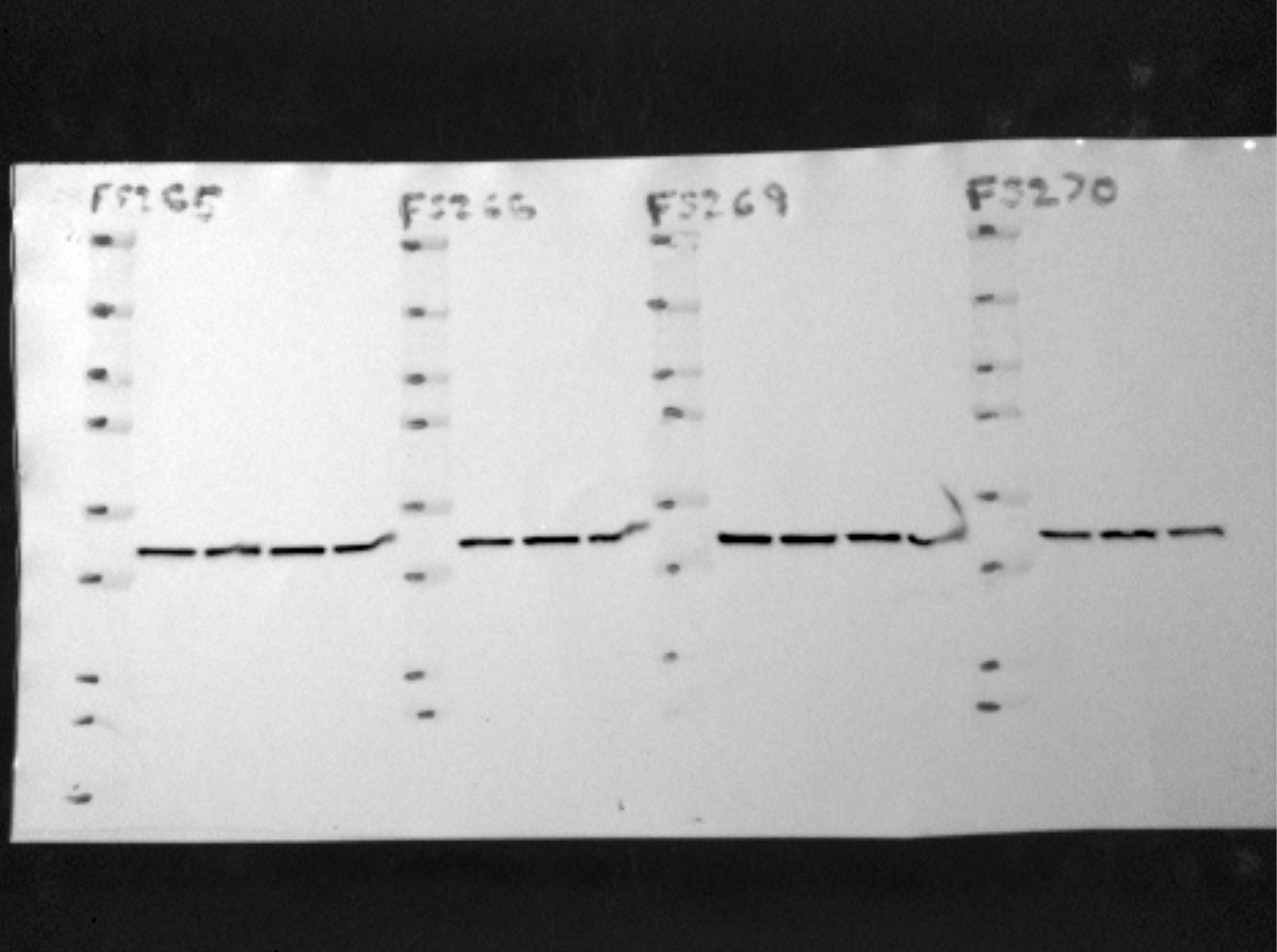

Supplement: Figure 2—source data 1. [file elife-71047-fig2-data1.zip › Figure 2A Actin.tif]

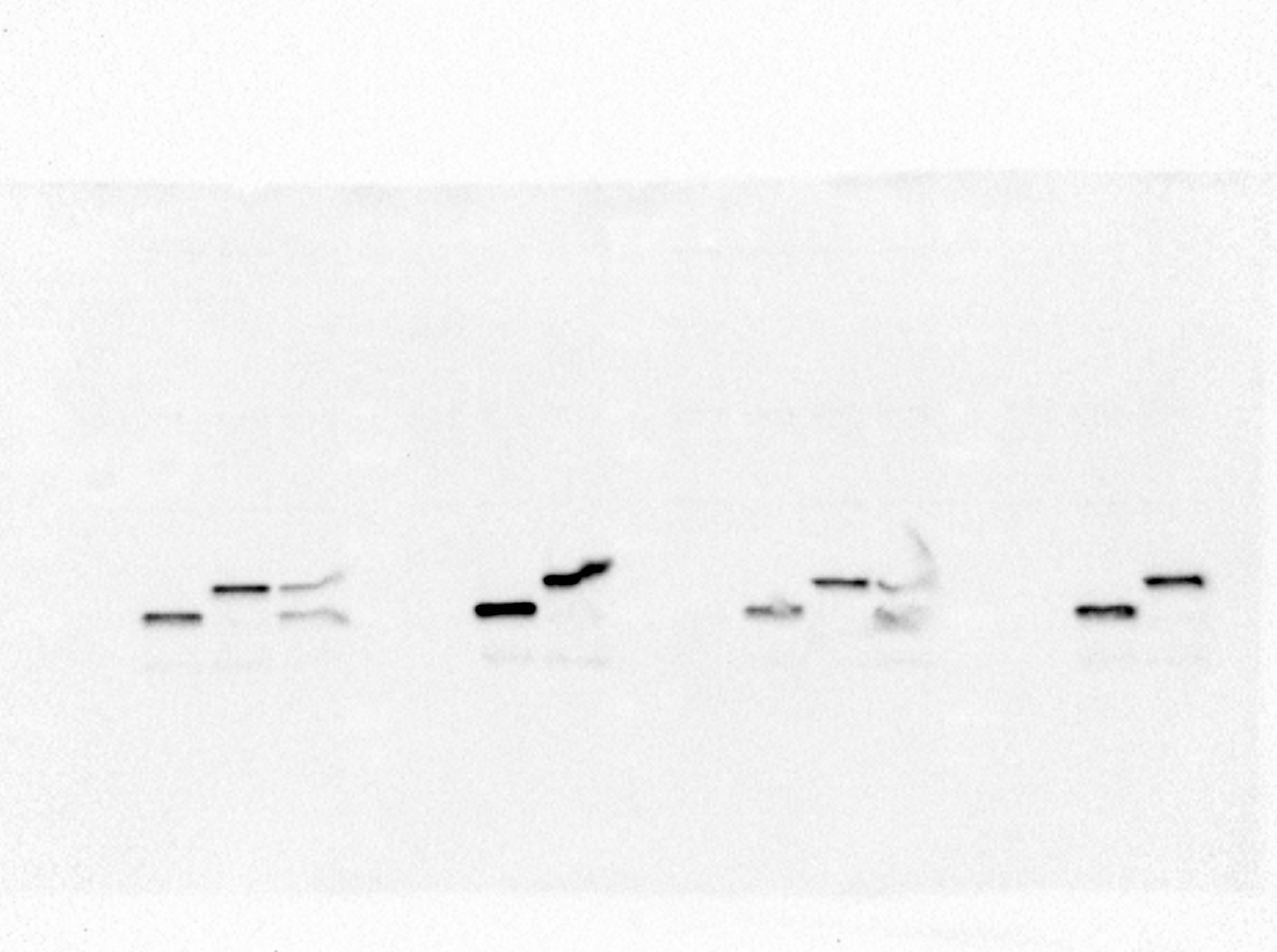

Supplement: Figure 2—source data 1. [file elife-71047-fig2-data1.zip › Figure 2A OAS1.tif]

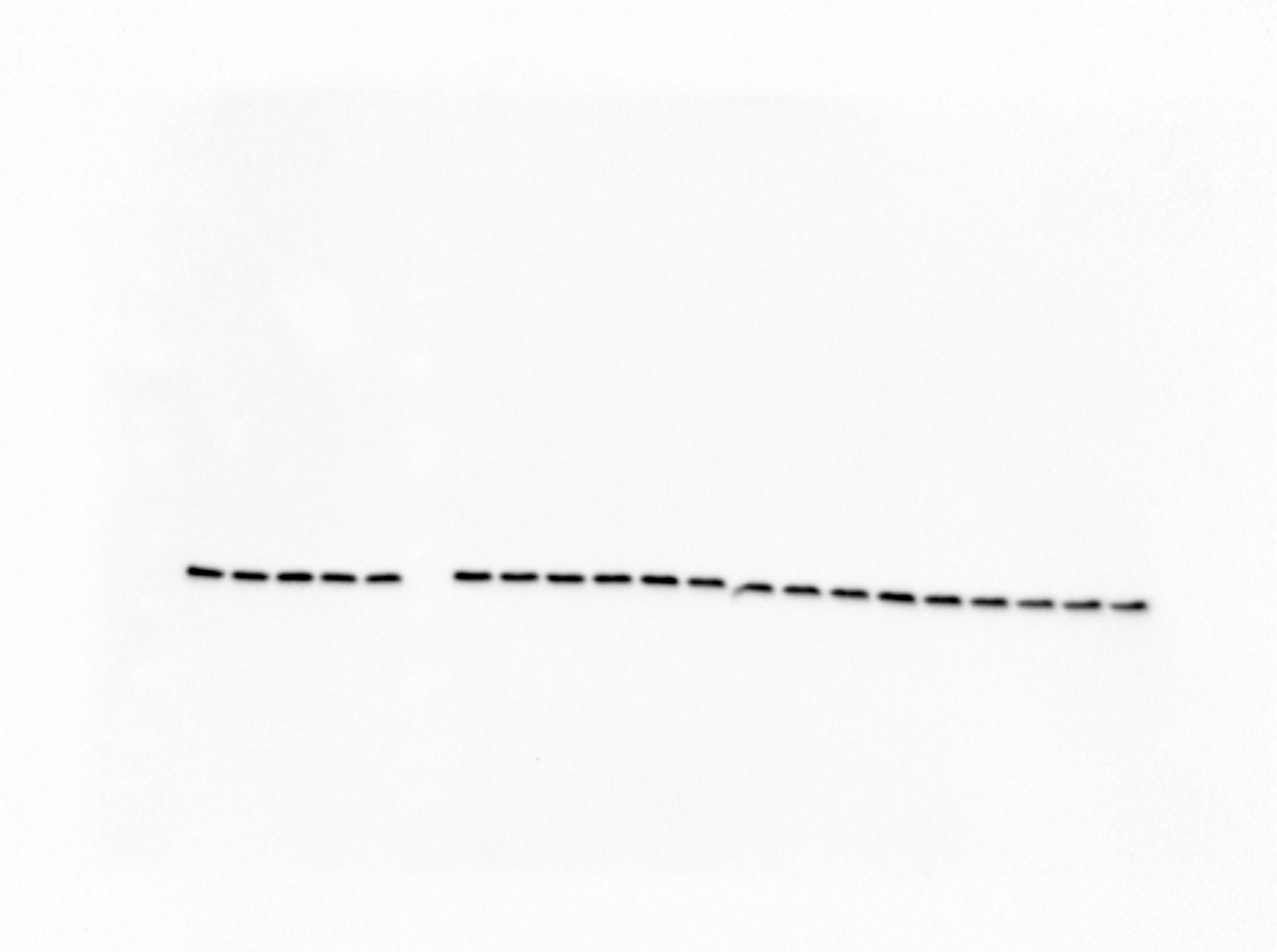

Supplement: Figure 2—source data 1. [file elife-71047-fig2-data1.zip › Figure 2D Actin.tif]

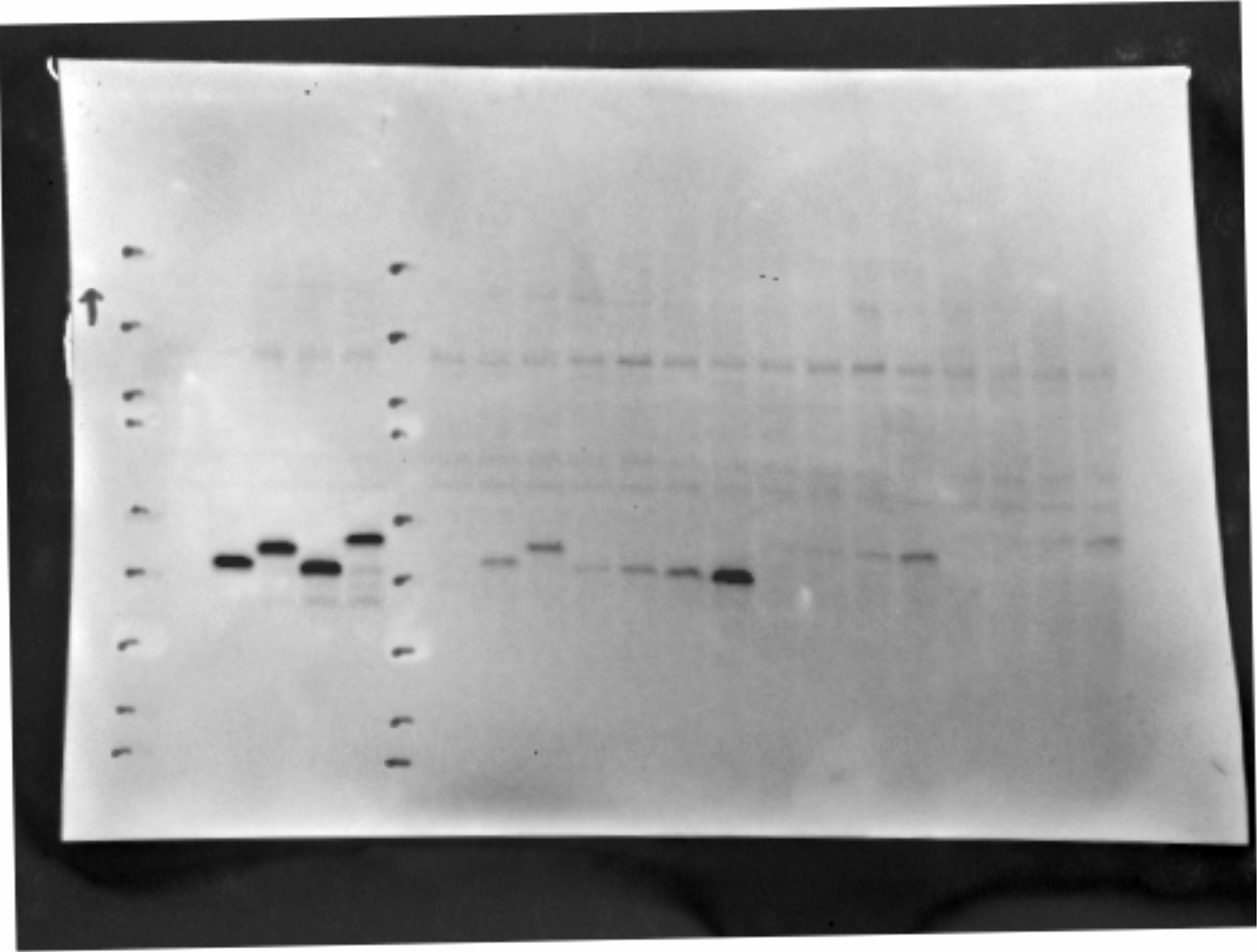

Supplement: Figure 2—source data 1. [file elife-71047-fig2-data1.zip › Figure 2D OAS1.tif]

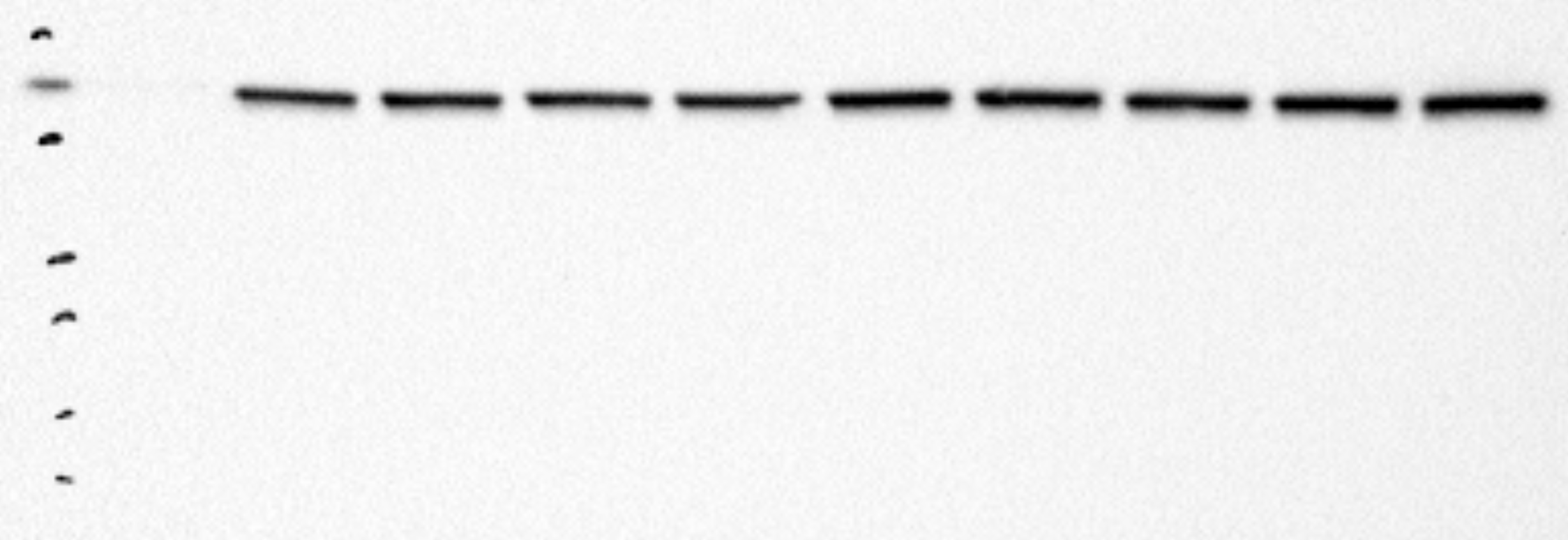

Supplement: Figure 2—source data 1. [file elife-71047-fig2-data1.zip › Figure 2F Actin.tif]

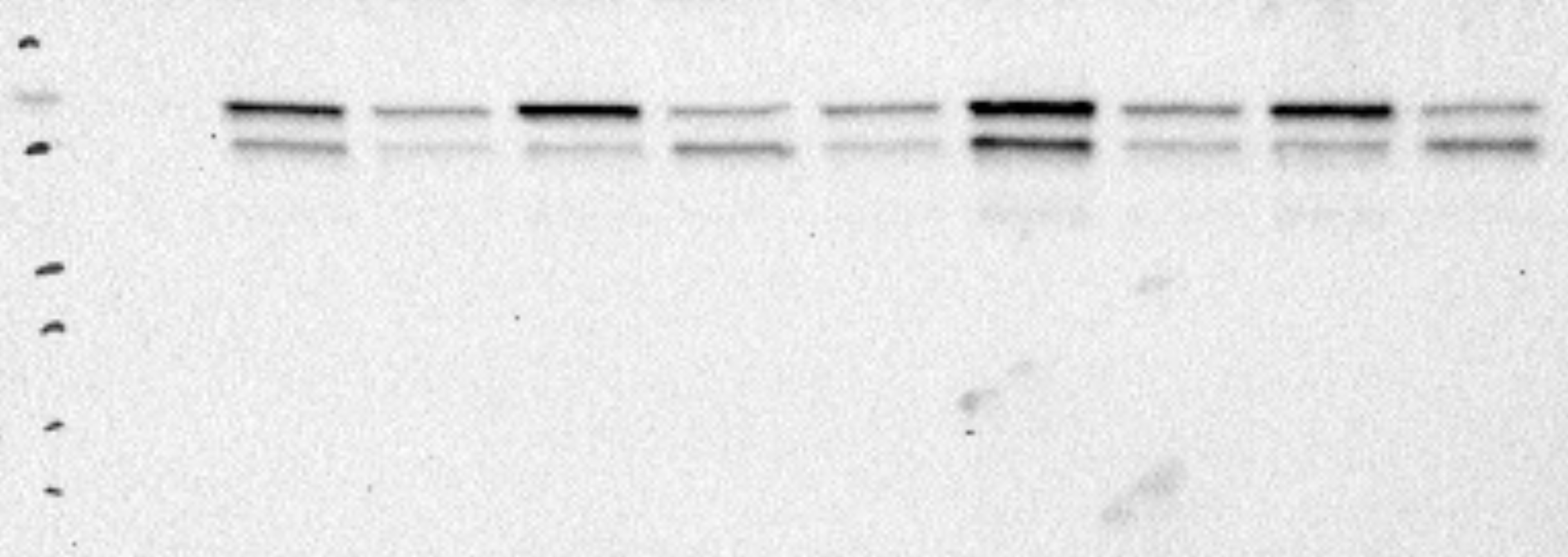

Supplement: Figure 2—source data 1. [file elife-71047-fig2-data1.zip › Figure 2F OAS1.tif]

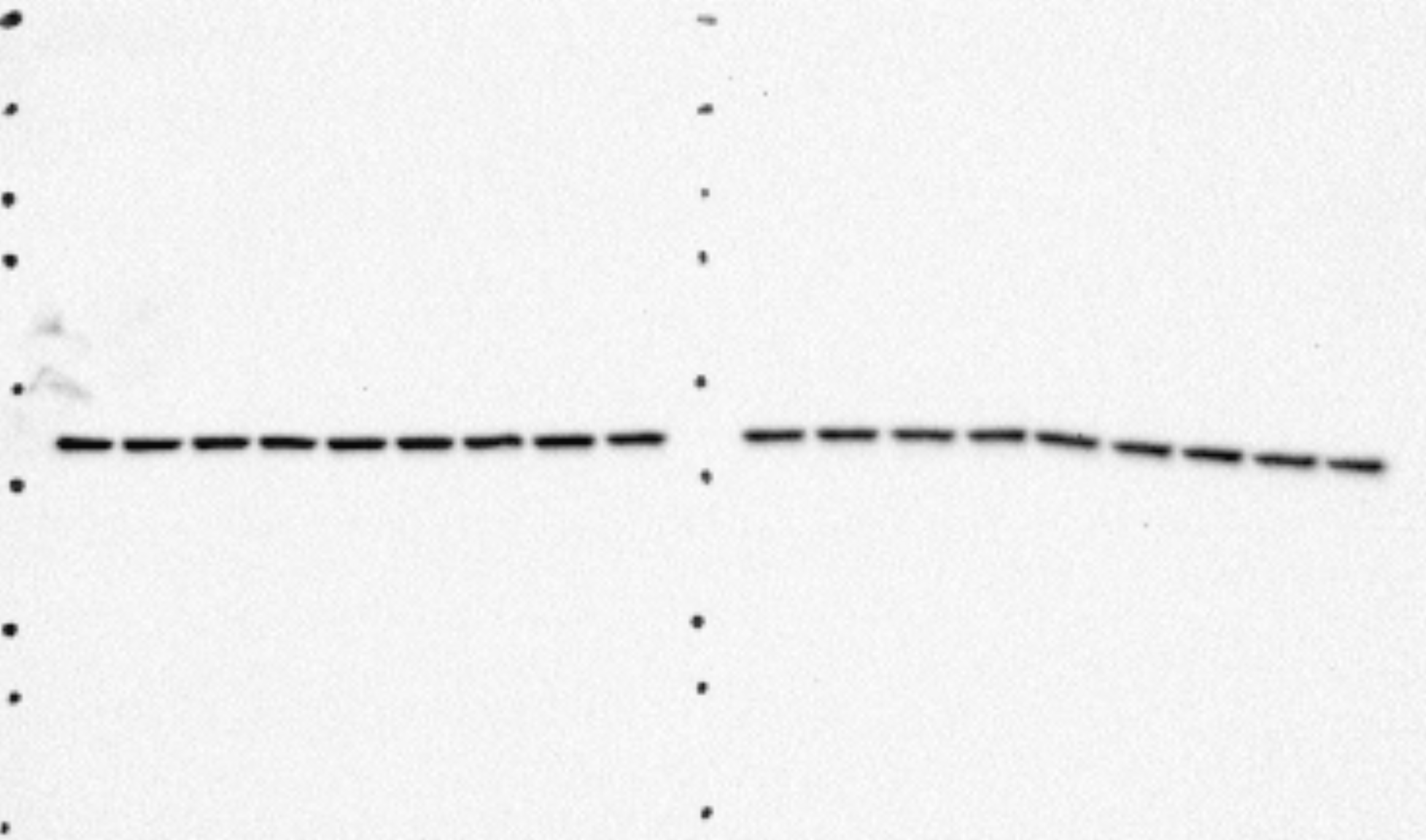

Supplement: Figure 3—source data 1. [file elife-71047-fig3-data1.zip › Figure 3 - Figure Supplement 1A Actin.tif]

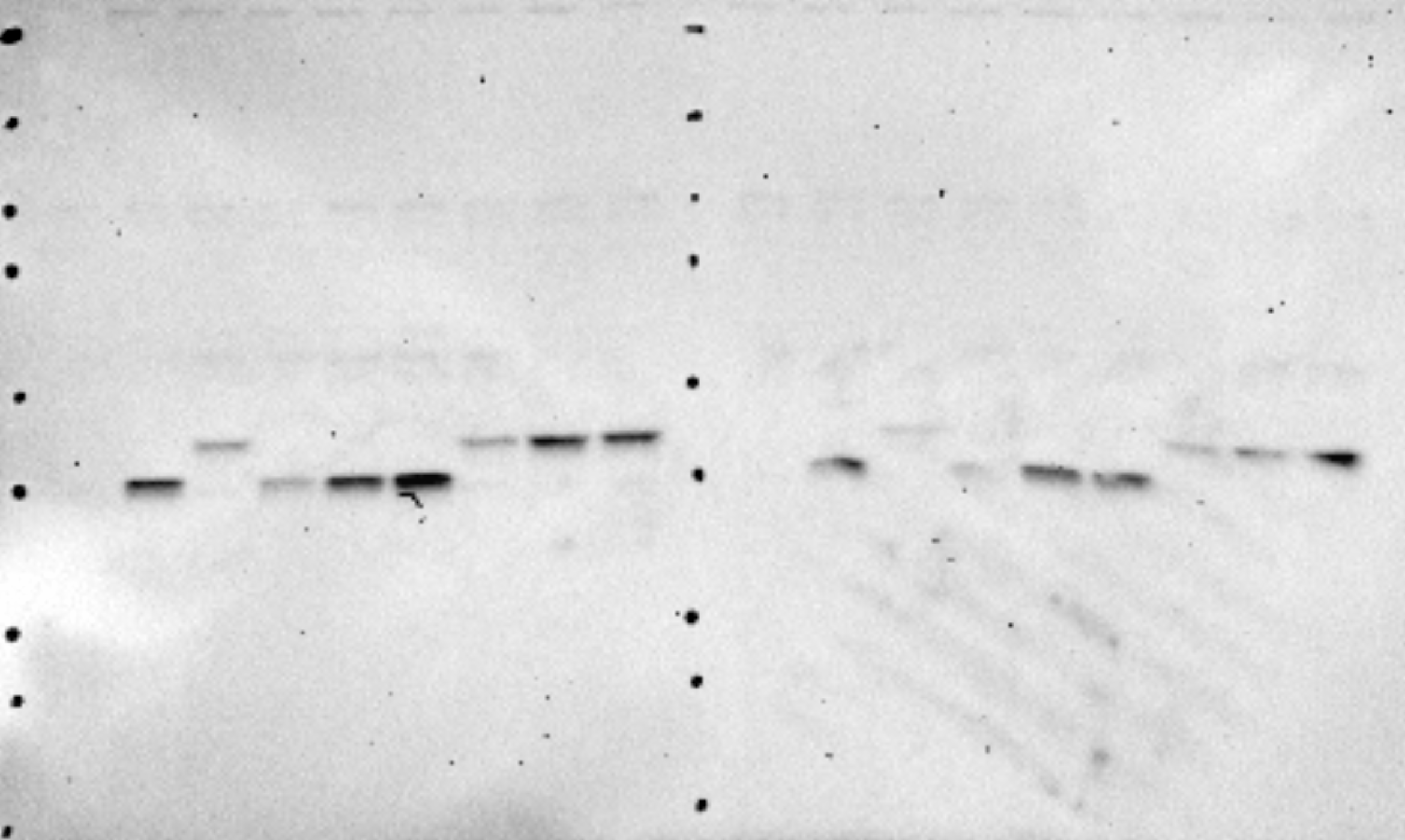

Supplement: Figure 3—source data 1. [file elife-71047-fig3-data1.zip › Figure 3 - Figure Supplement 1A OAS1.tif]

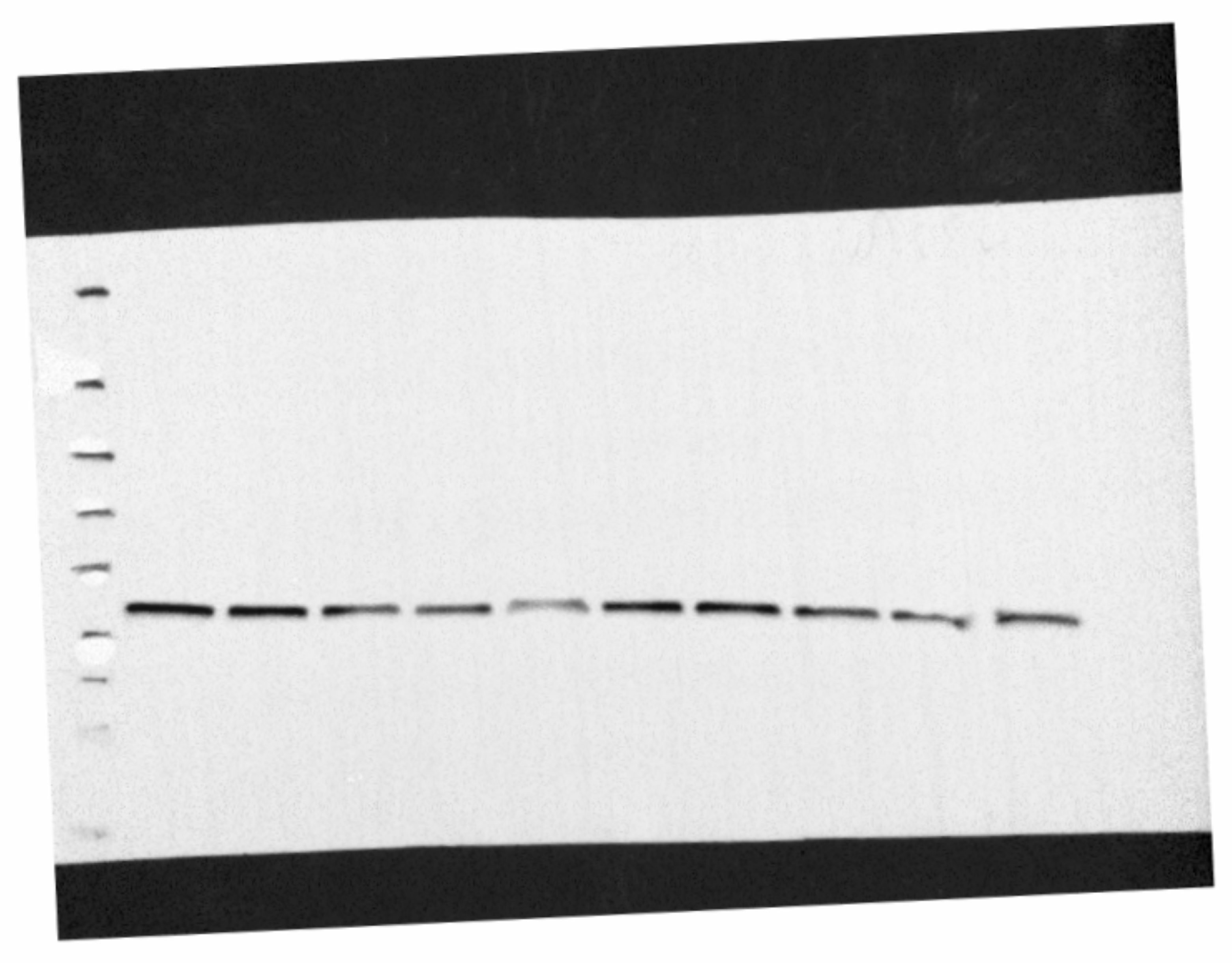

Supplement: Figure 3—source data 1. [file elife-71047-fig3-data1.zip › Figure 3 - Figure Supplement 1C Actin.tif]

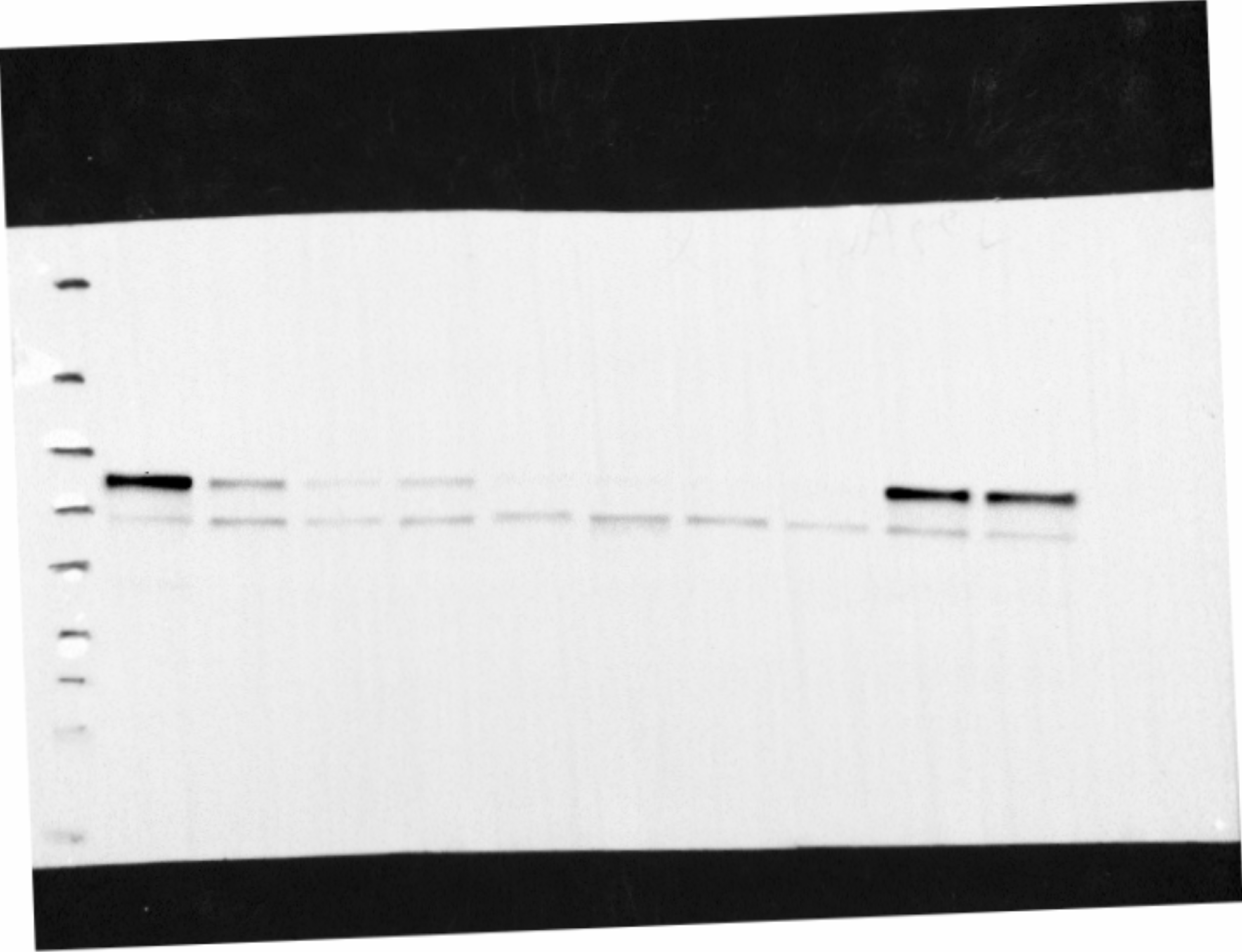

Supplement: Figure 3—source data 1. [file elife-71047-fig3-data1.zip › Figure 3 - Figure Supplement 1C RNaseL.tif]

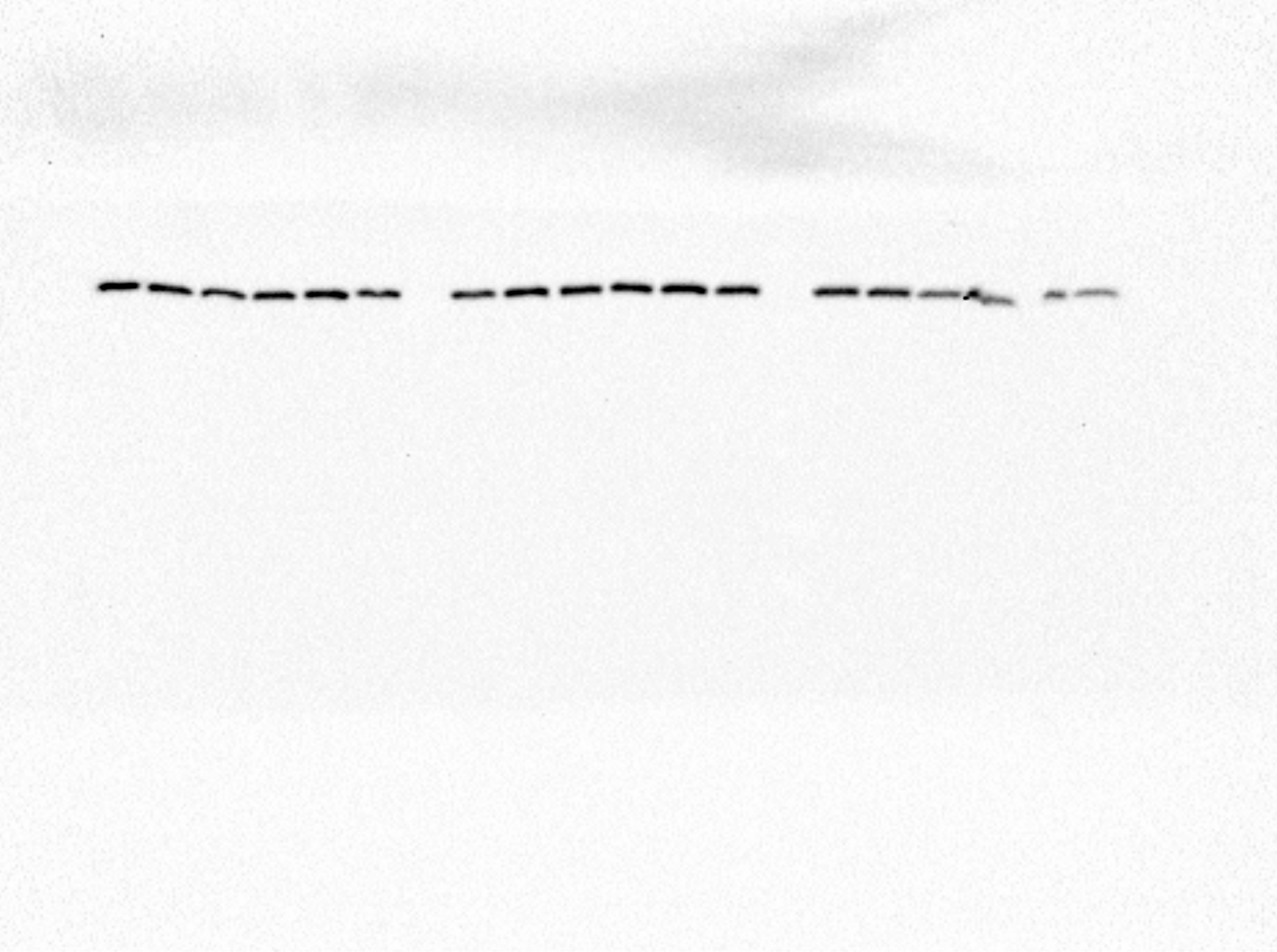

Supplement: Figure 3—source data 1. [file elife-71047-fig3-data1.zip › Figure 3 - Figure Supplement 1D Actin.tif]

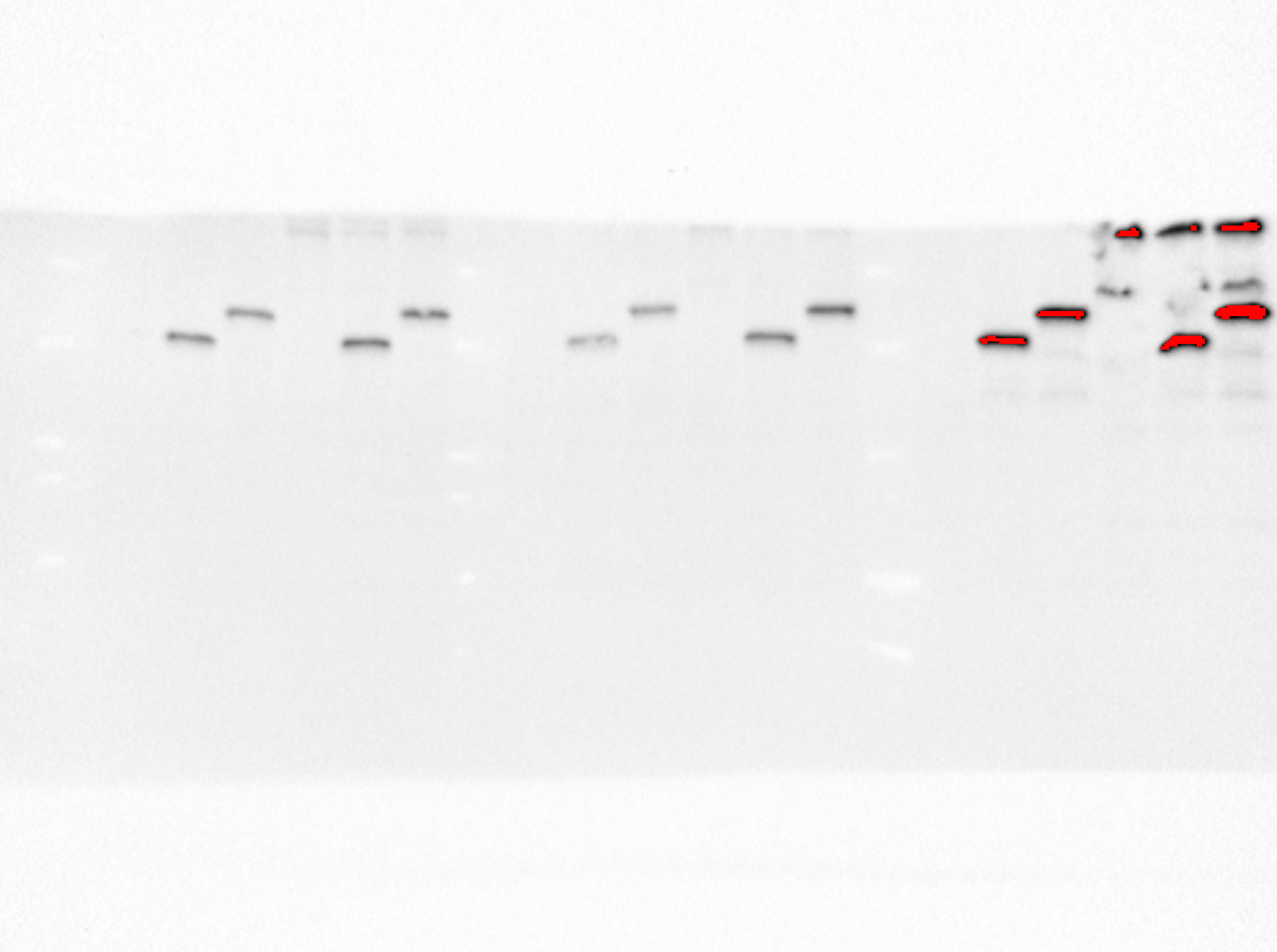

Supplement: Figure 3—source data 1. [file elife-71047-fig3-data1.zip › Figure 3 - Figure Supplement 1D OAS1.tif]

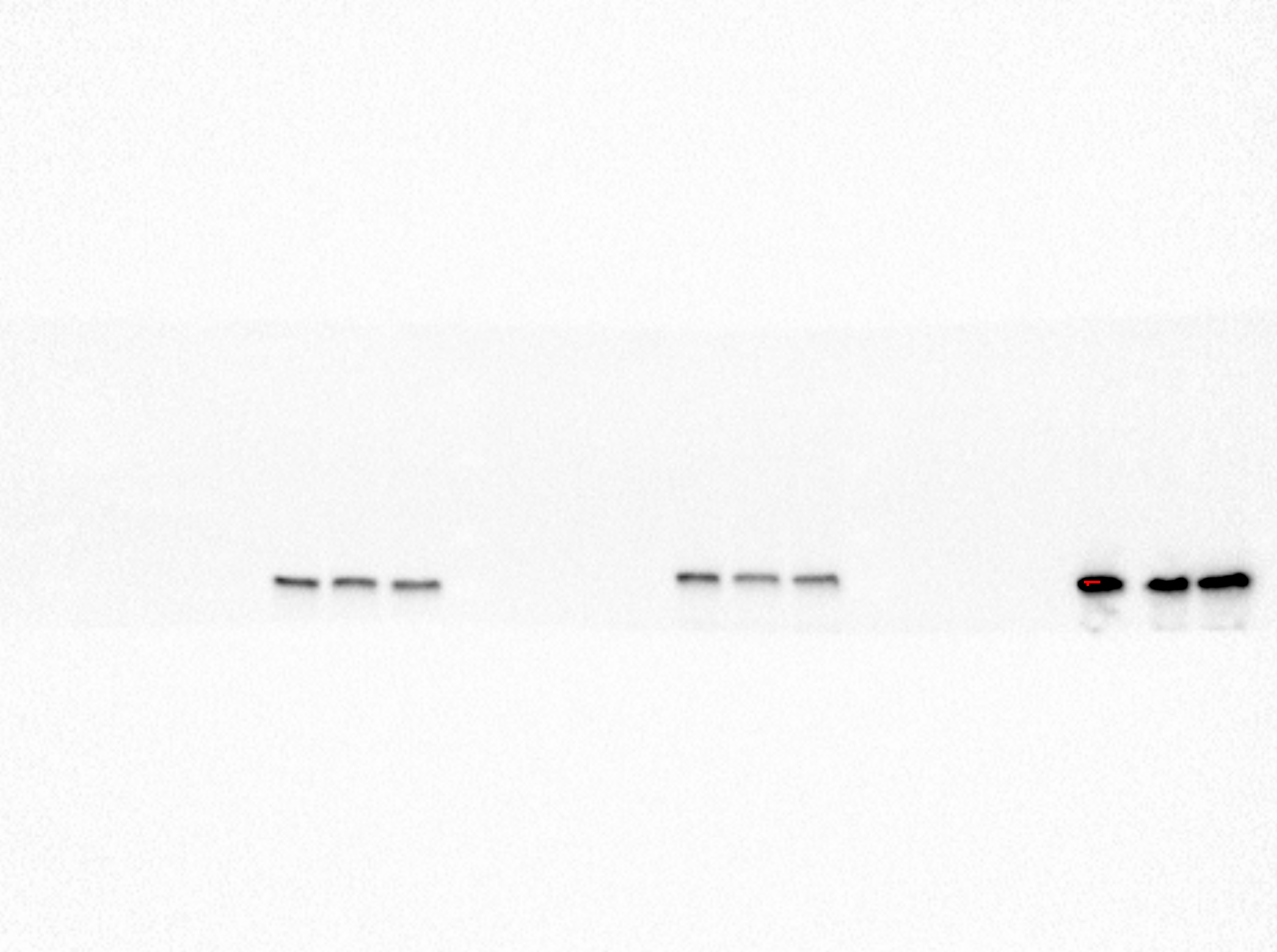

Supplement: Figure 3—source data 1. [file elife-71047-fig3-data1.zip › Figure 3 - Figure Supplement 1D RNaseL.tif]

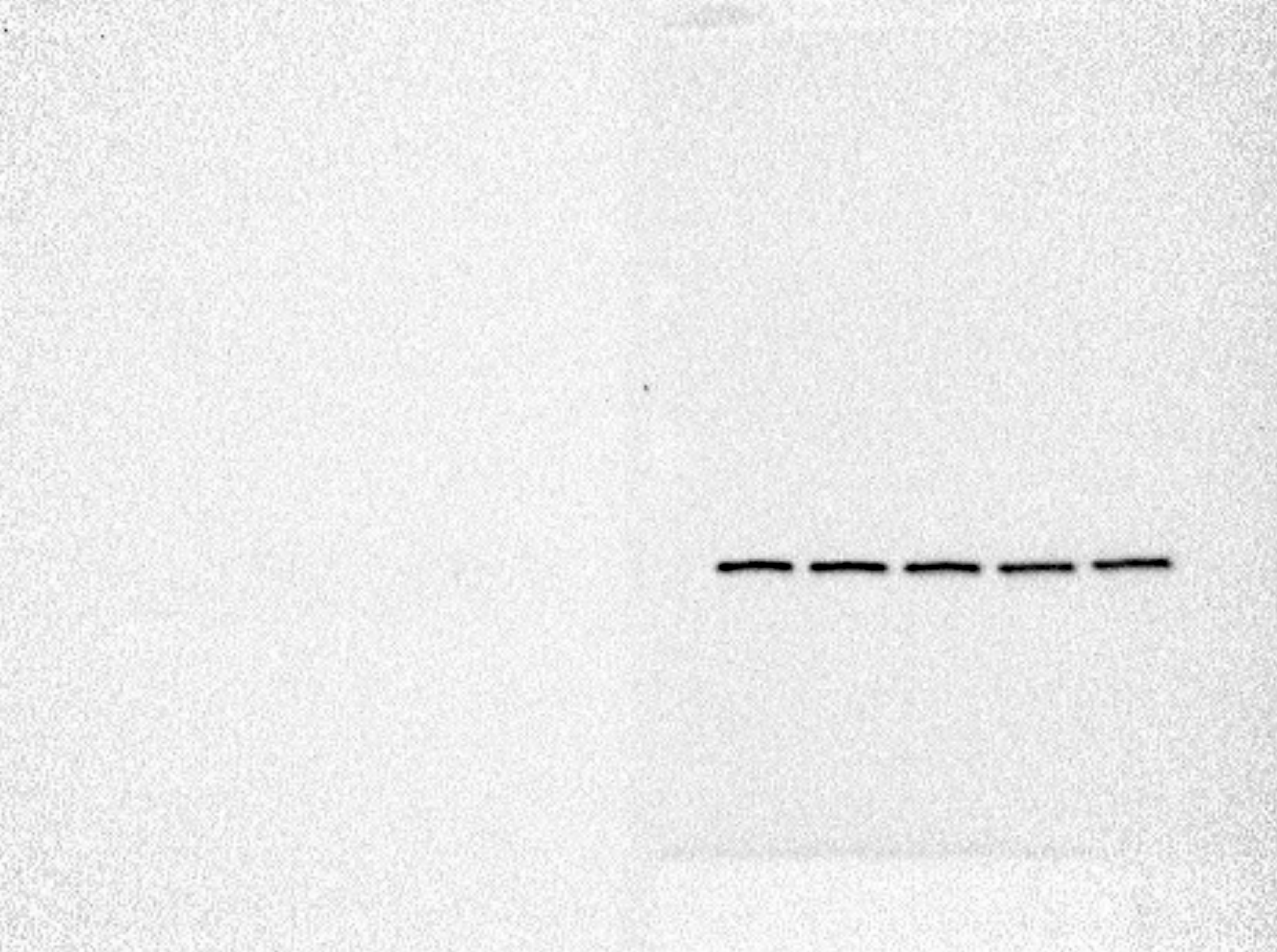

Supplement: Figure 3—source data 1. [file elife-71047-fig3-data1.zip › Figure 3A Actin.tif]

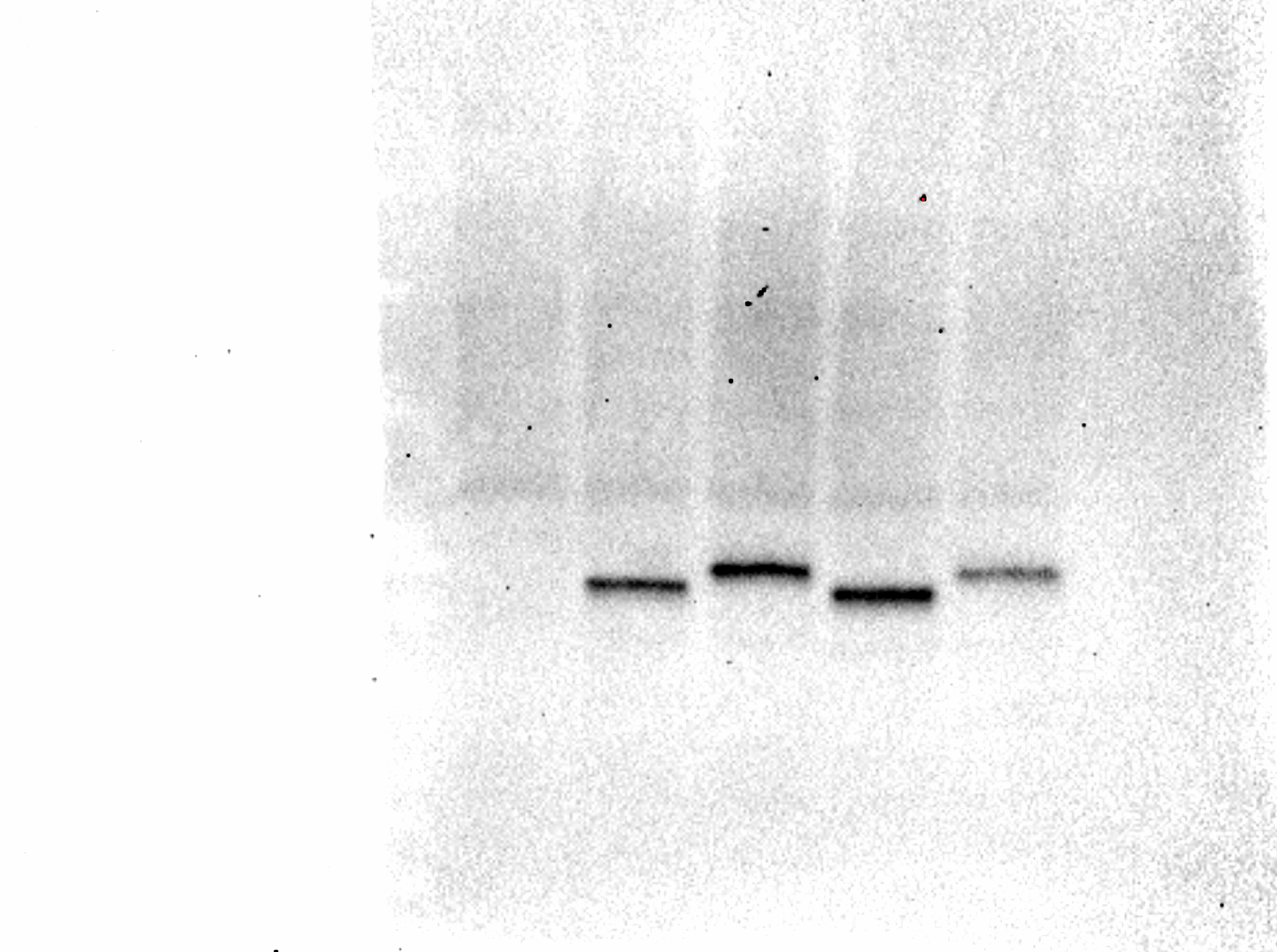

Supplement: Figure 3—source data 1. [file elife-71047-fig3-data1.zip › Figure 3A OAS1.tif]

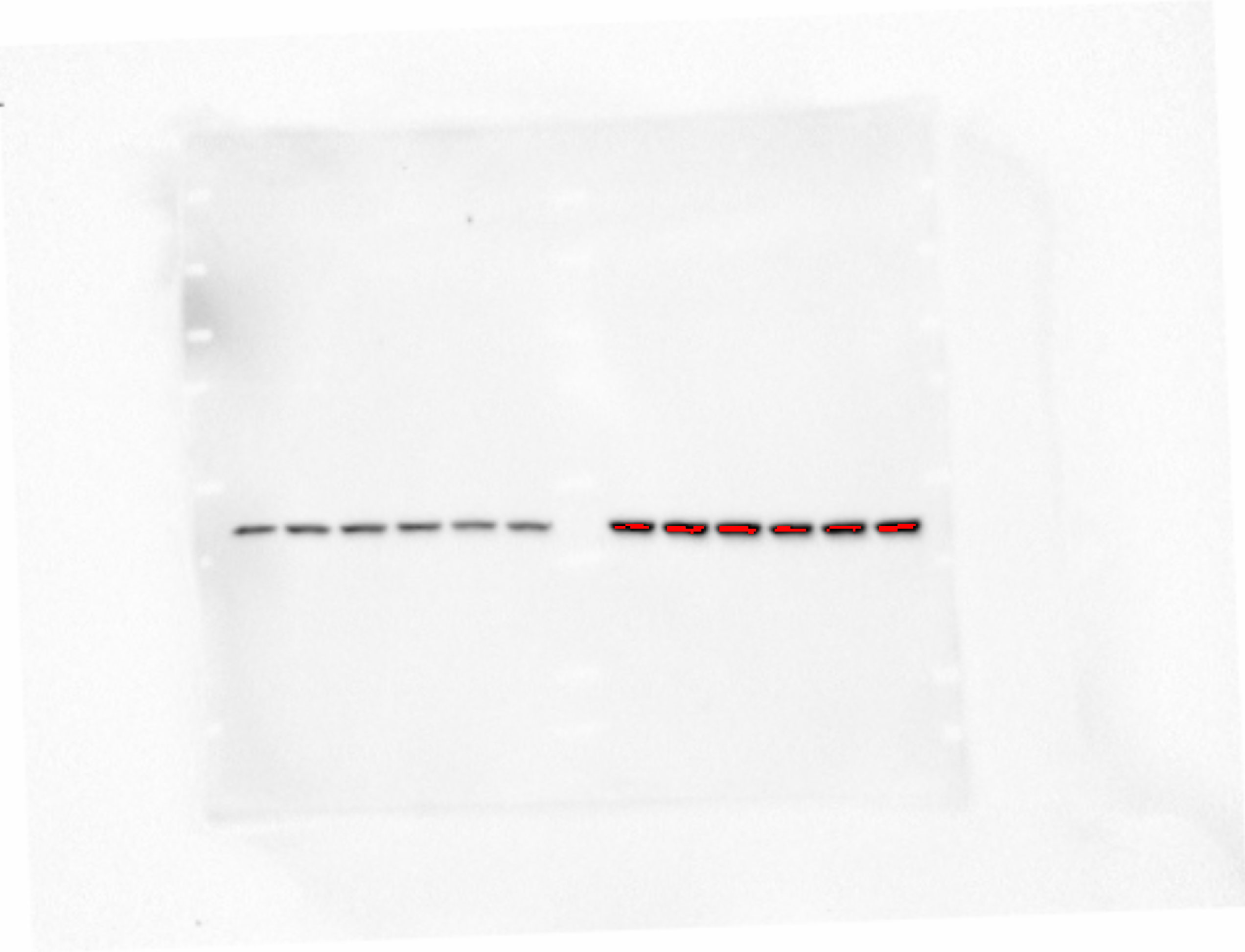

Supplement: Figure 3—source data 1. [file elife-71047-fig3-data1.zip › Figure 3D Actin.tif]

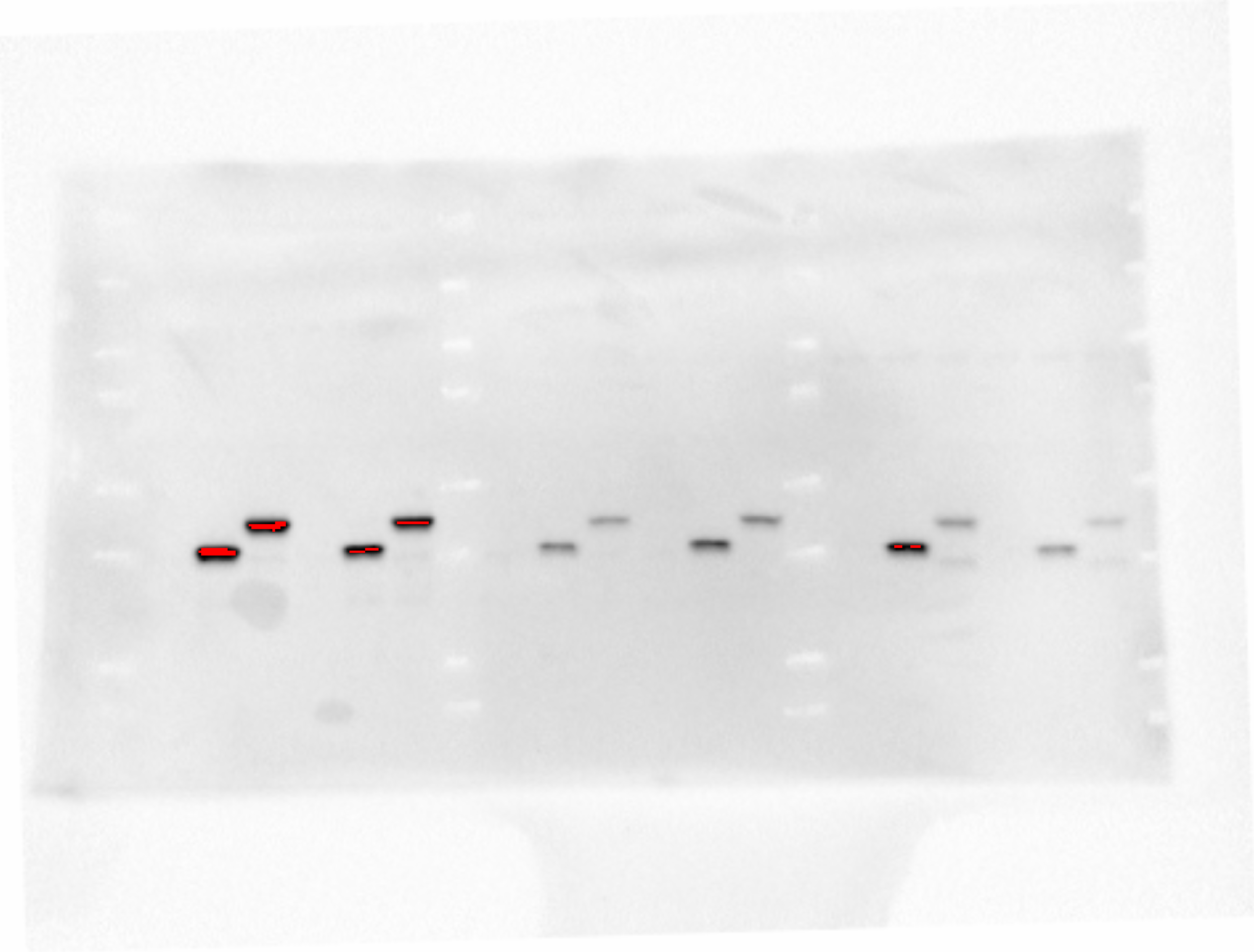

Supplement: Figure 3—source data 1. [file elife-71047-fig3-data1.zip › Figure 3D OAS1.tif]

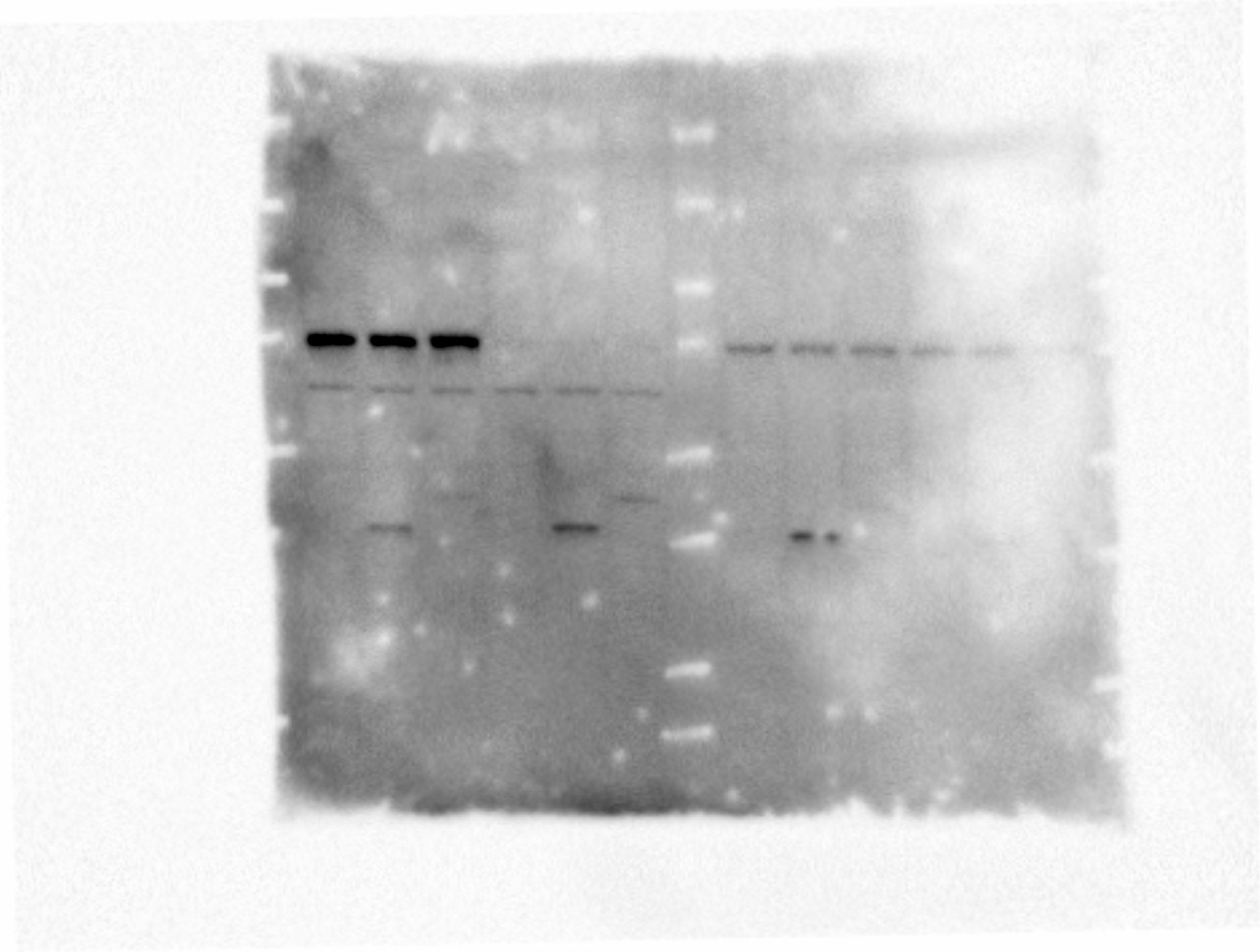

Supplement: Figure 3—source data 1. [file elife-71047-fig3-data1.zip › Figure 3D RNaseL.tif]

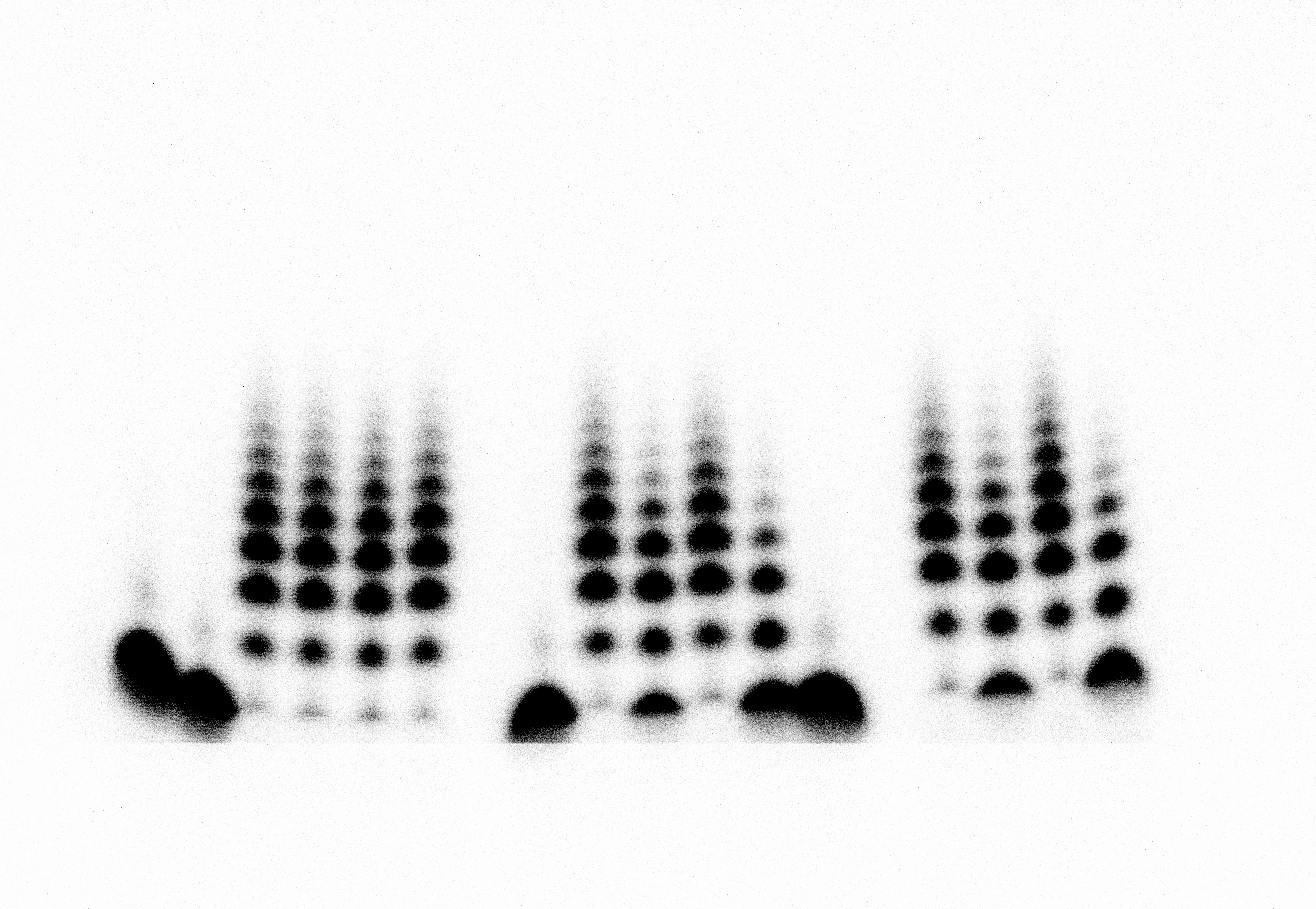

Supplement: Figure 3—source data 1. [file elife-71047-fig3-data1.zip › Figure 3G.jpg]

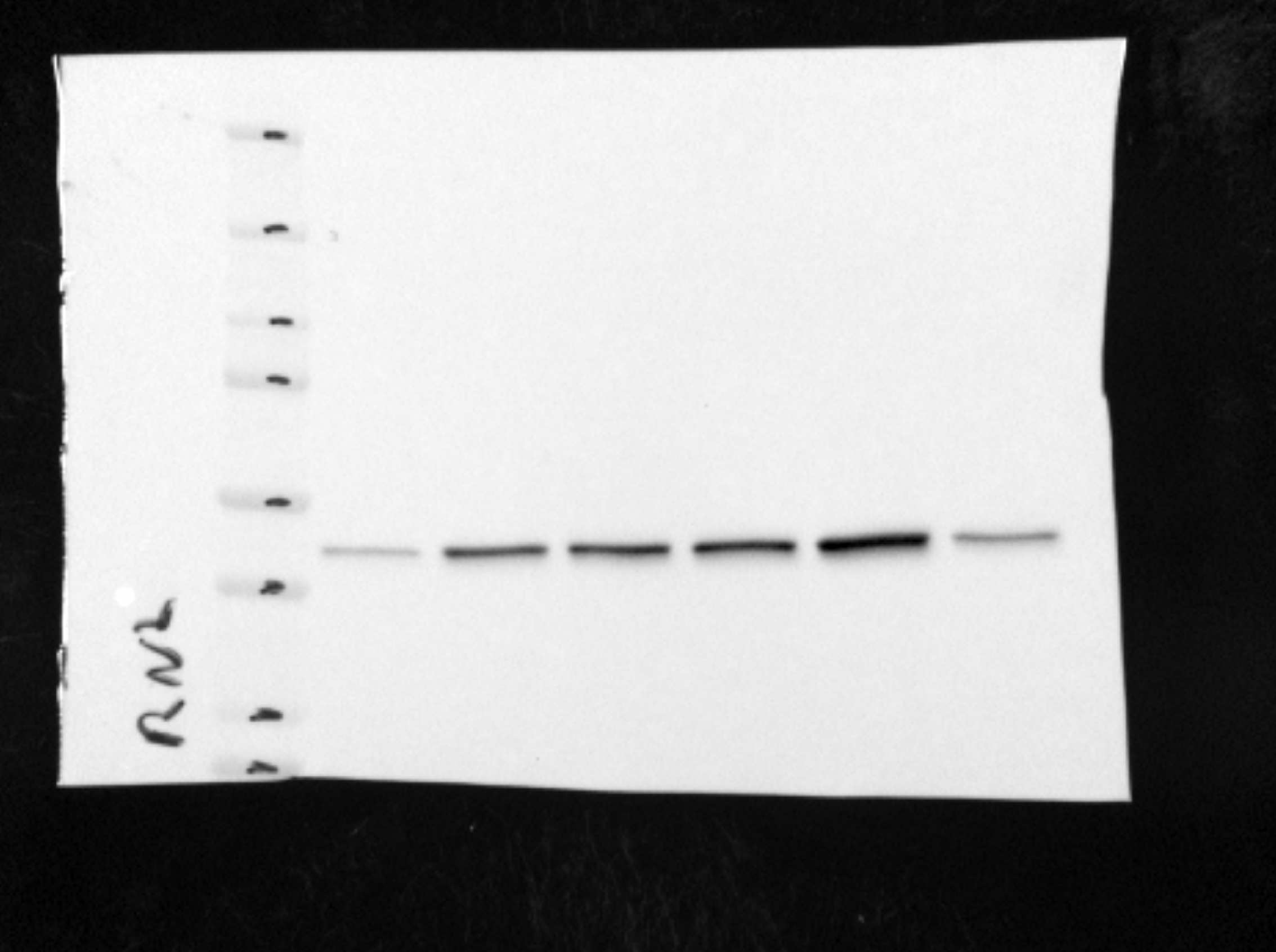

Supplement: Figure 4—source data 1. [file elife-71047-fig4-data1.zip › Figure 4 - Figure Supplement 1A Actin.tif]

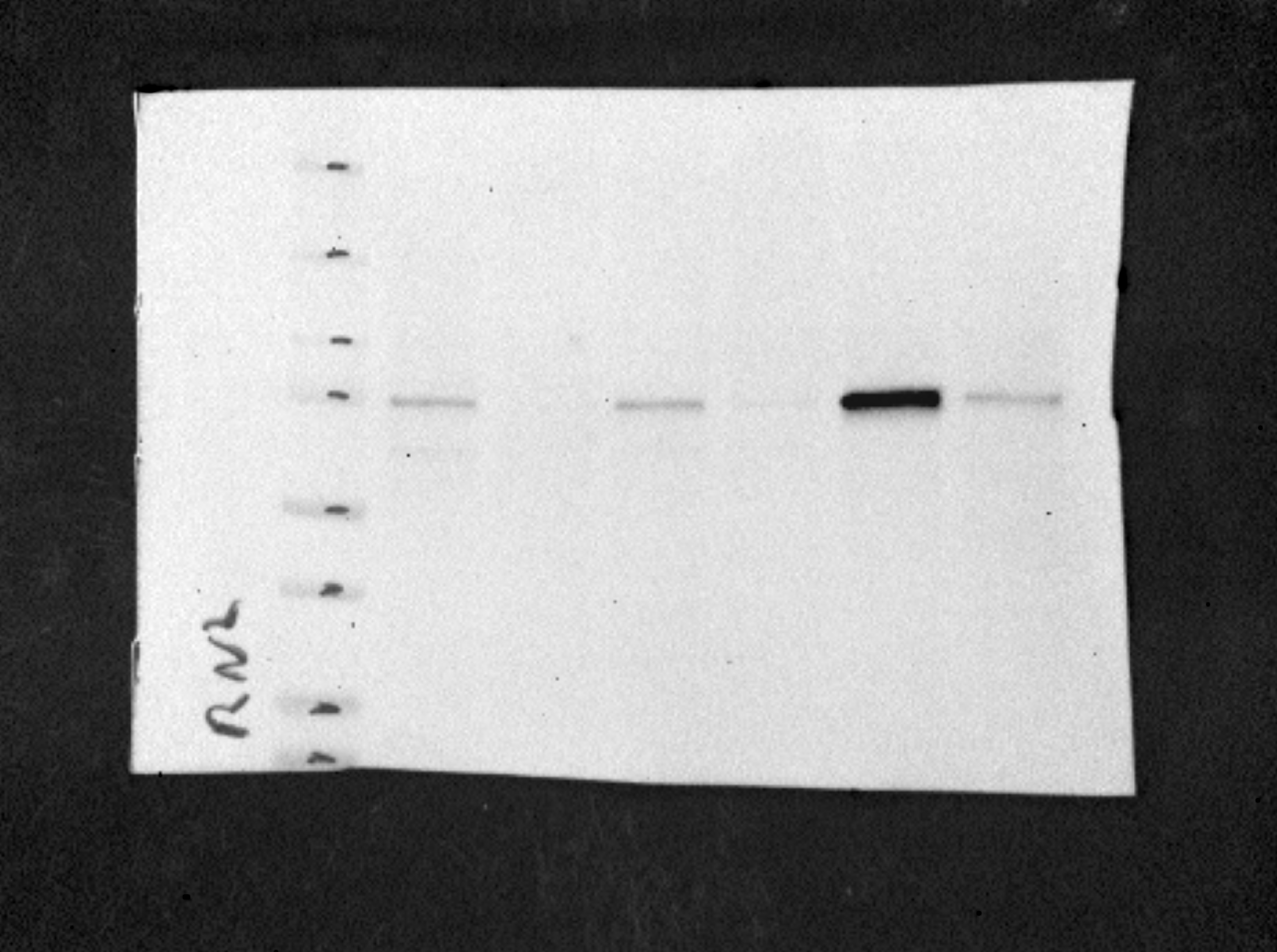

Supplement: Figure 4—source data 1. [file elife-71047-fig4-data1.zip › Figure 4 - Figure Supplement 1A RNaseL.tif]

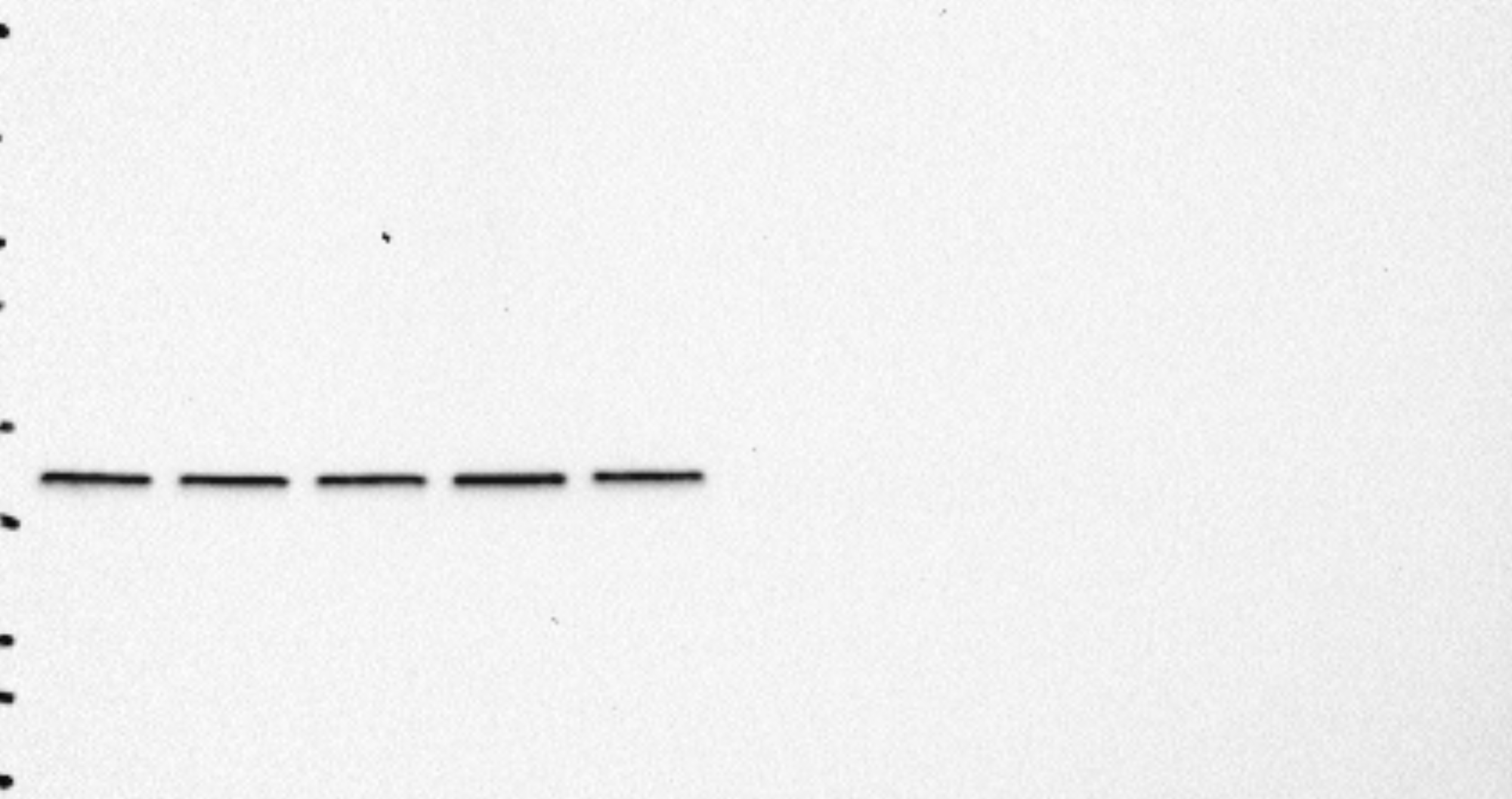

Supplement: Figure 4—source data 1. [file elife-71047-fig4-data1.zip › Figure 4B Actin.tif]

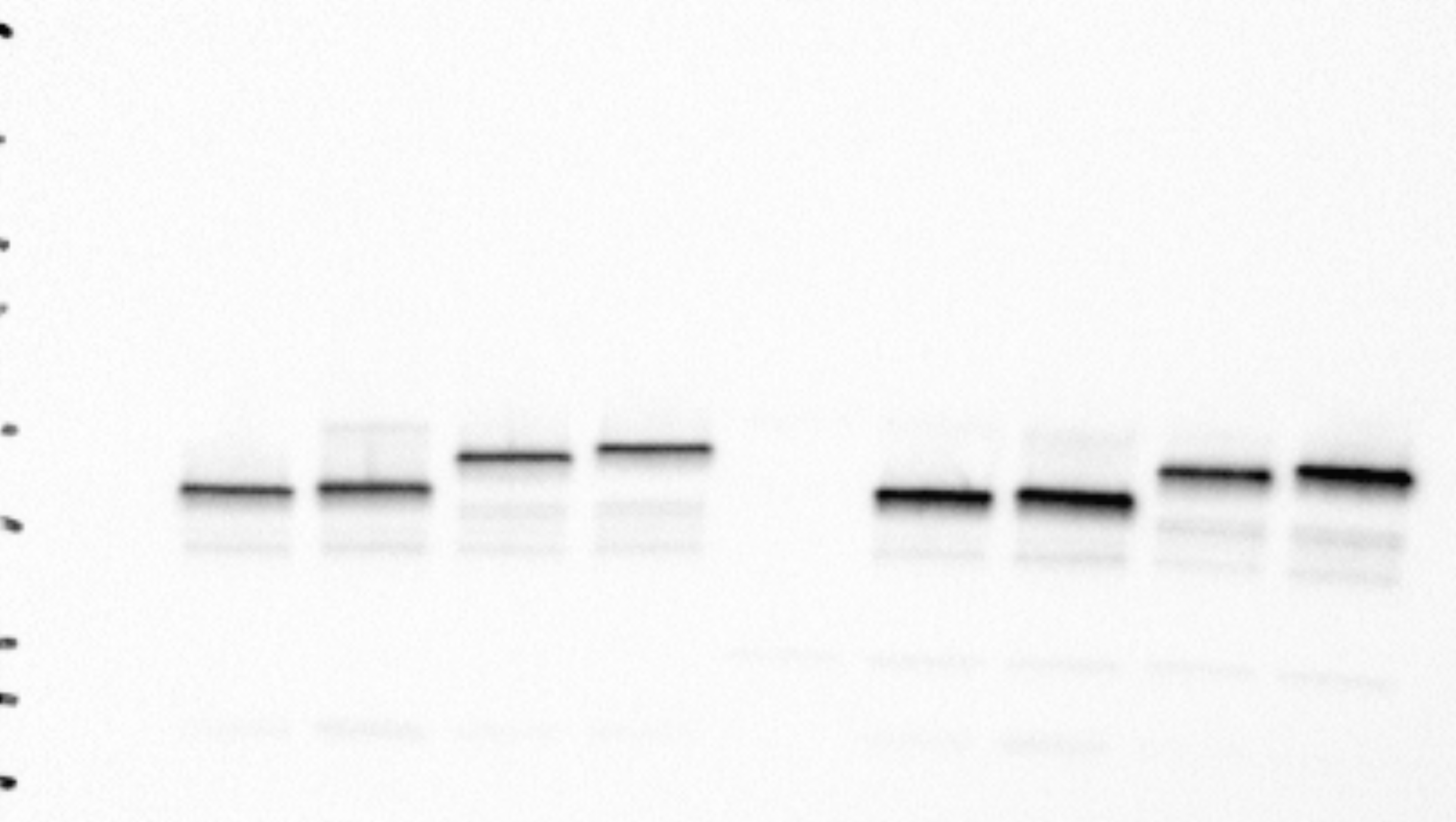

Supplement: Figure 4—source data 1. [file elife-71047-fig4-data1.zip › Figure 4B FLAG.tif]

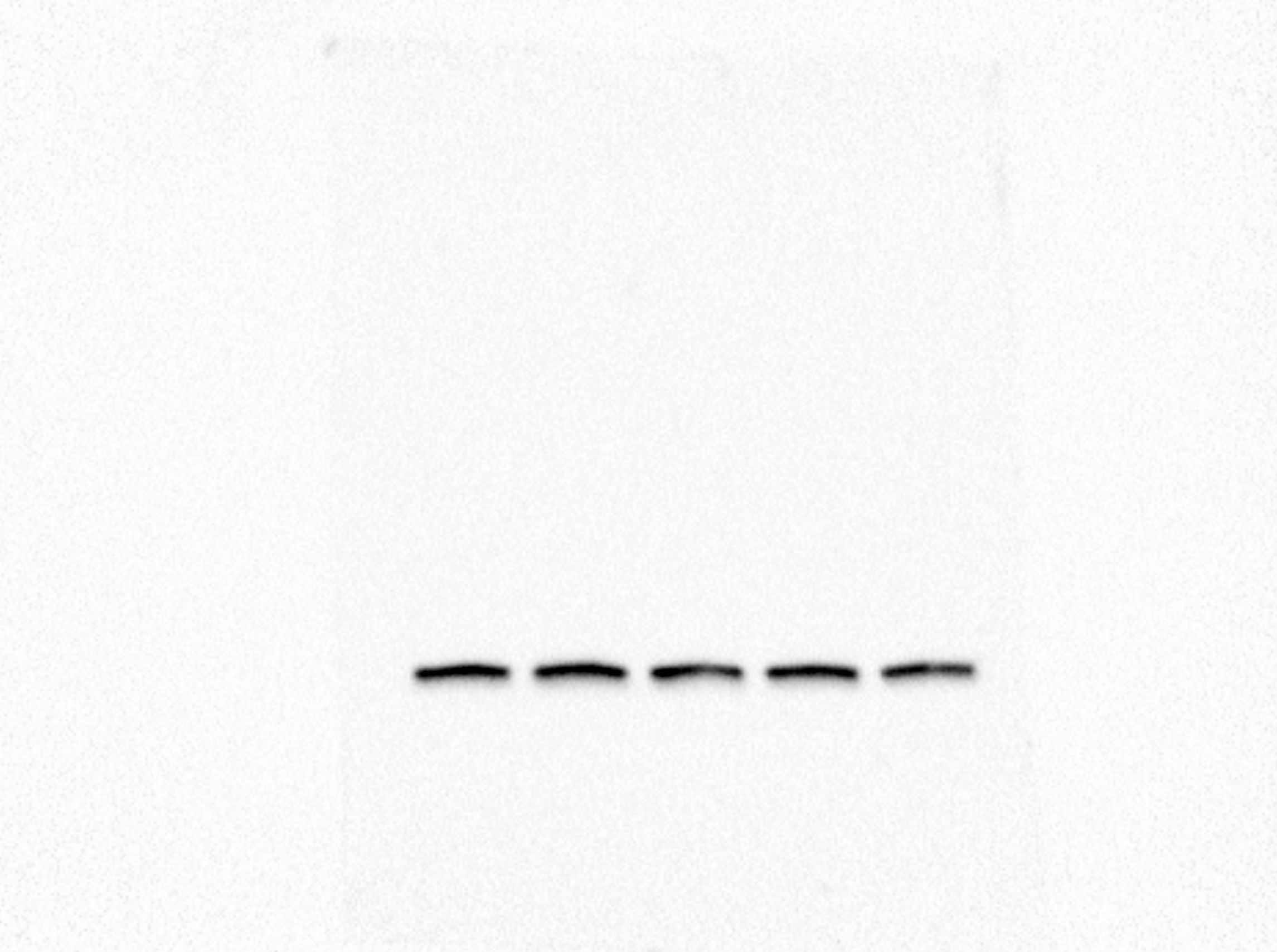

Supplement: Figure 4—source data 1. [file elife-71047-fig4-data1.zip › Figure 4C Actin.tif]

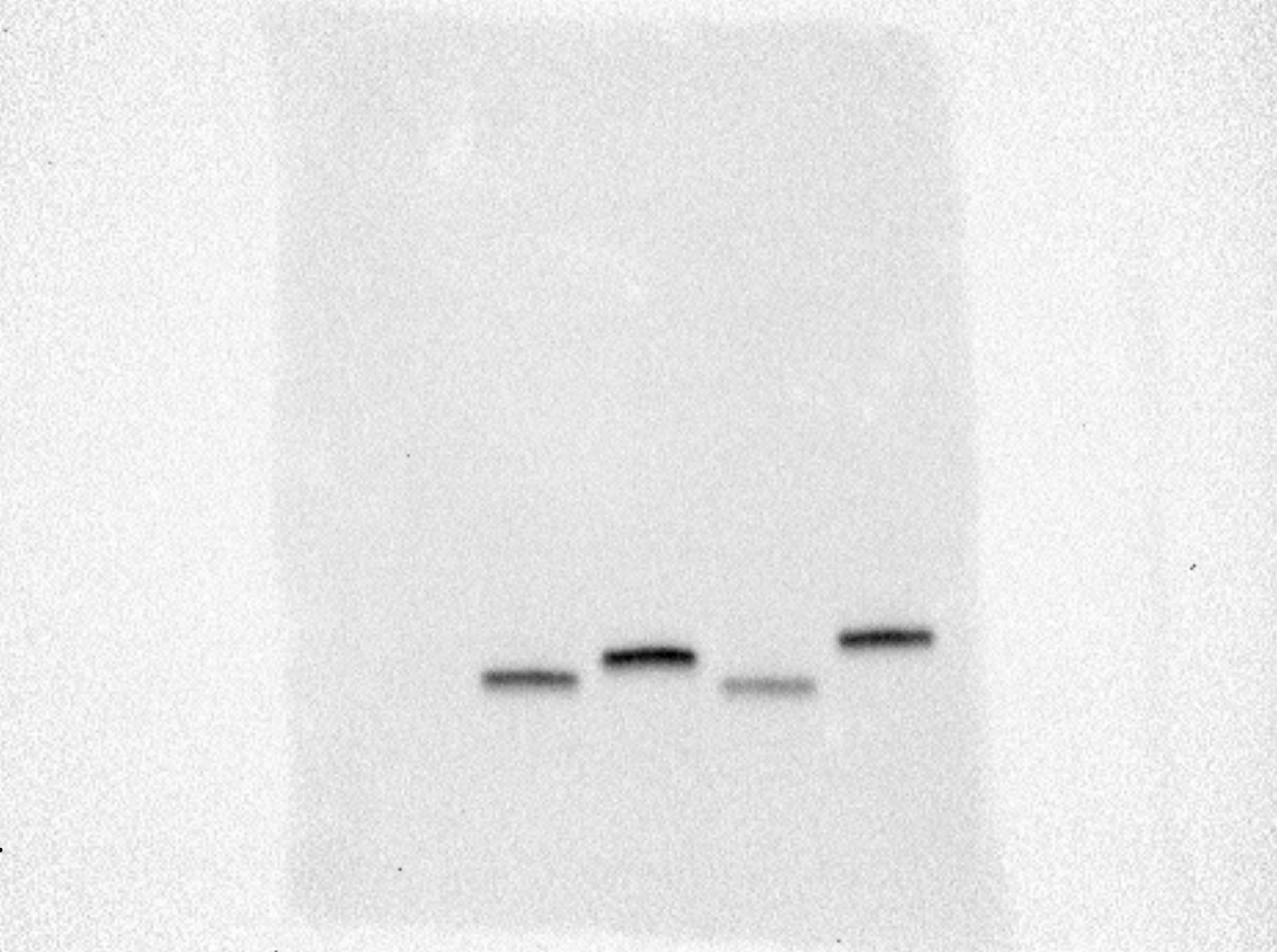

Supplement: Figure 4—source data 1. [file elife-71047-fig4-data1.zip › Figure 4C OAS1.tif]

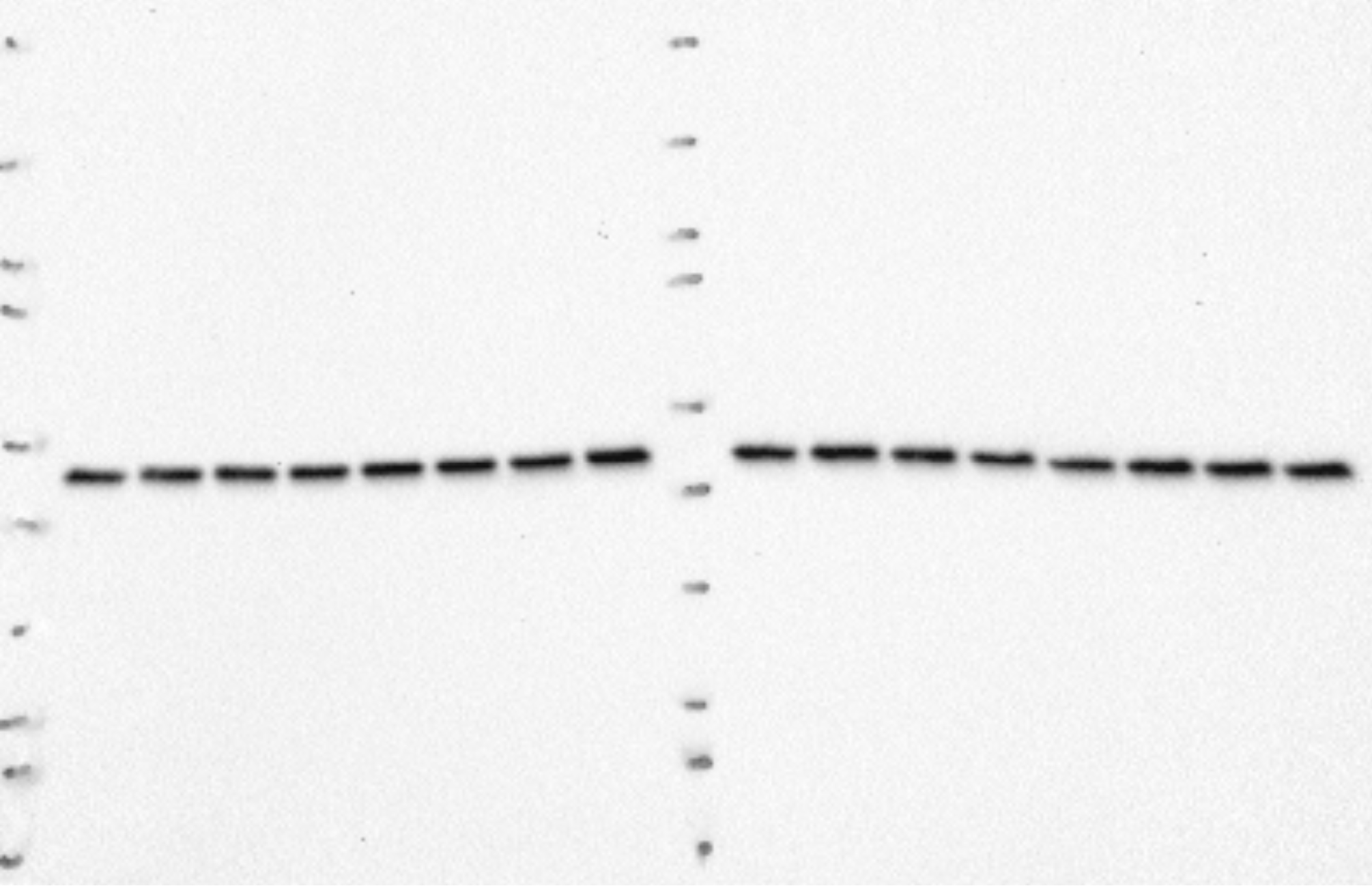

Supplement: Figure 4—source data 1. [file elife-71047-fig4-data1.zip › Figure 4G Actin.tif]

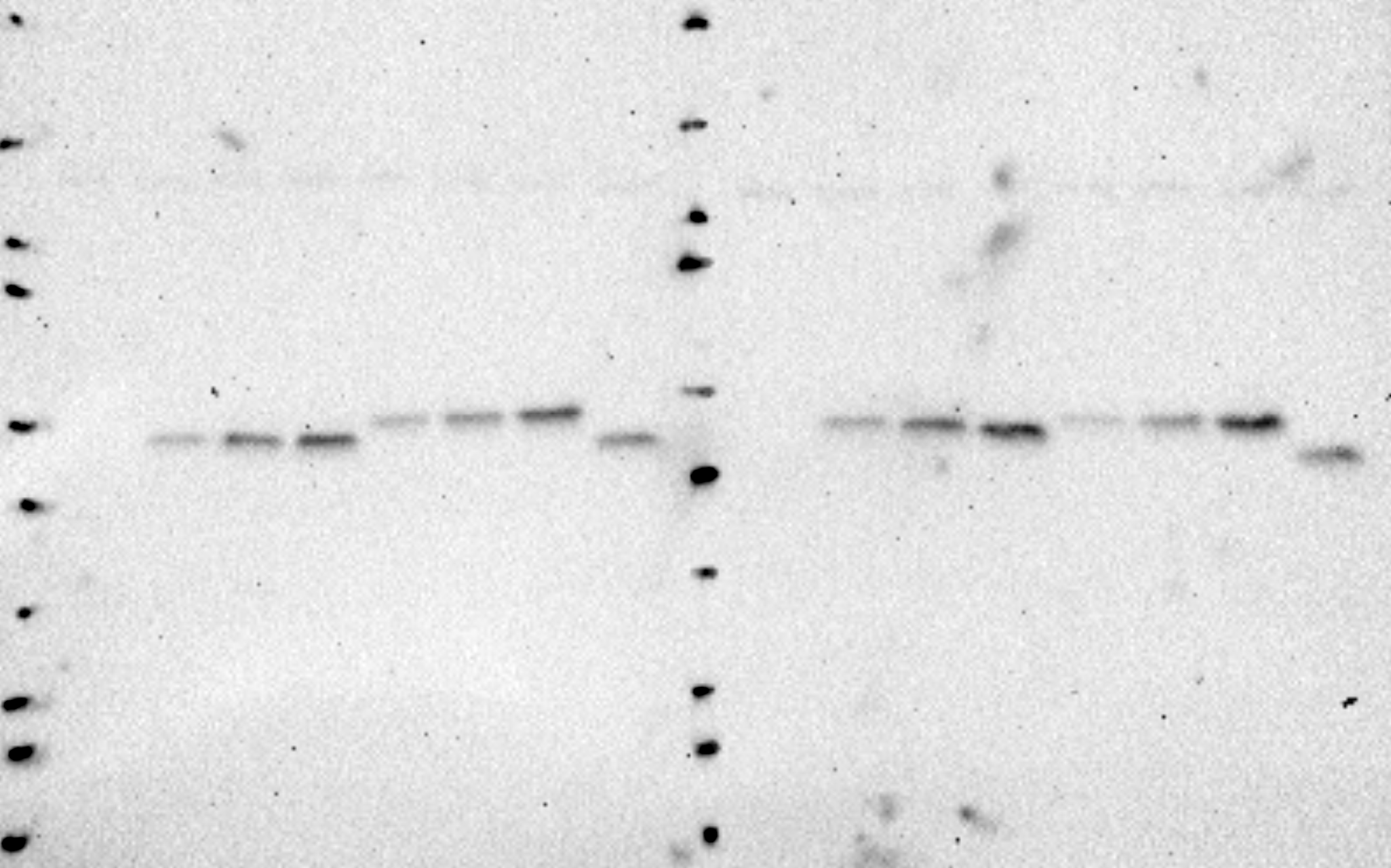

Supplement: Figure 4—source data 1. [file elife-71047-fig4-data1.zip › Figure 4G OAS1.tif]

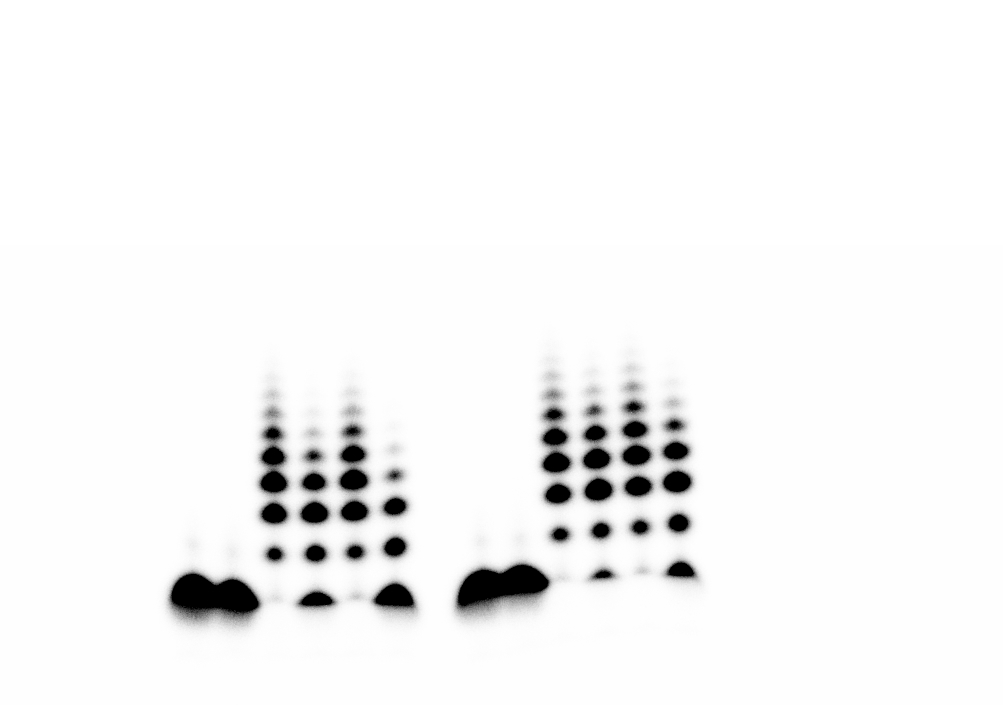

Supplement: Figure 5—source data 1. [file elife-71047-fig5-data1.zip › Figure 5 - Figure Supplement 1D.jpg]

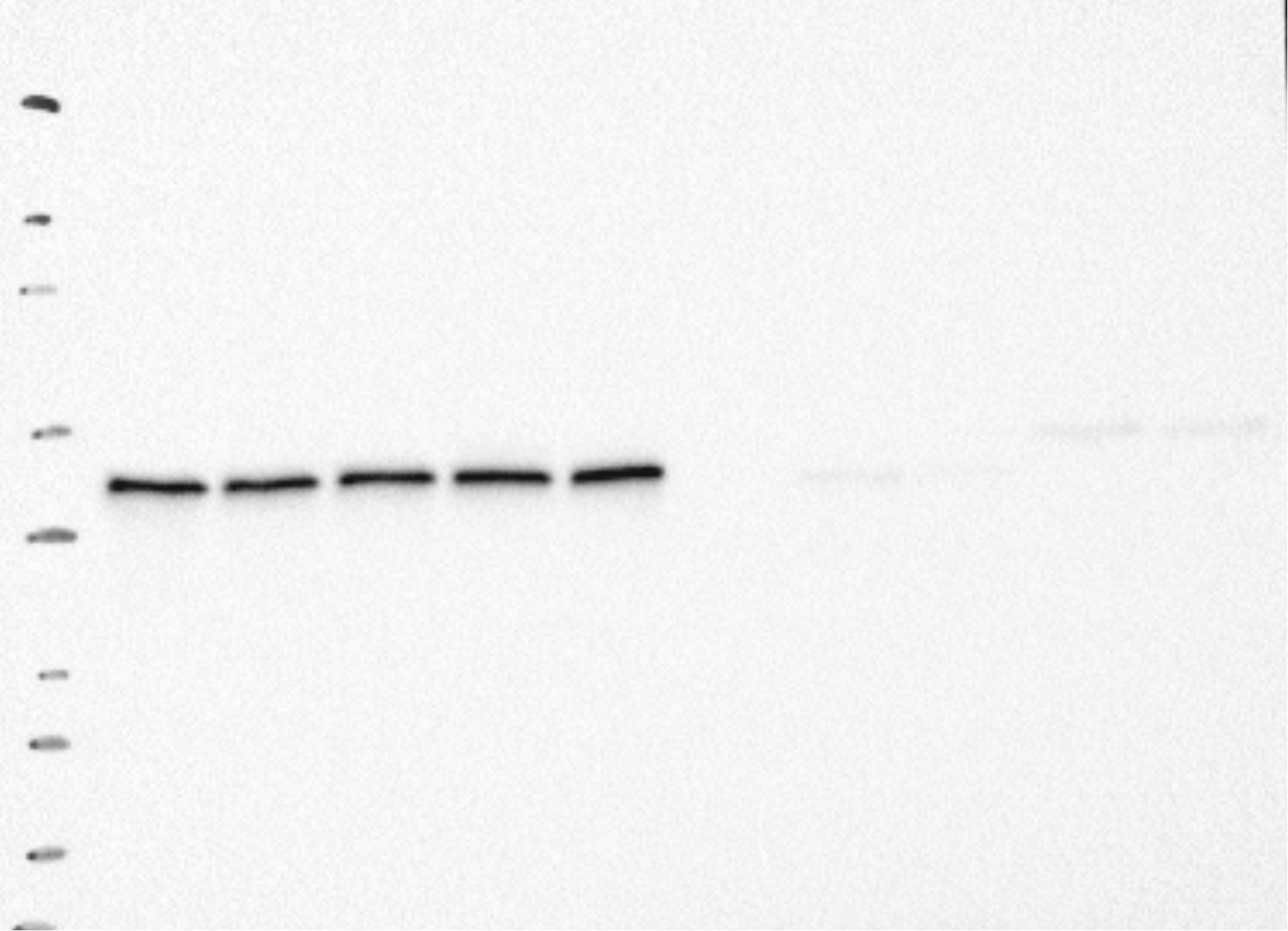

Supplement: Figure 5—source data 1. [file elife-71047-fig5-data1.zip › Figure 5 - Figure Supplement 1E Actin.tif]

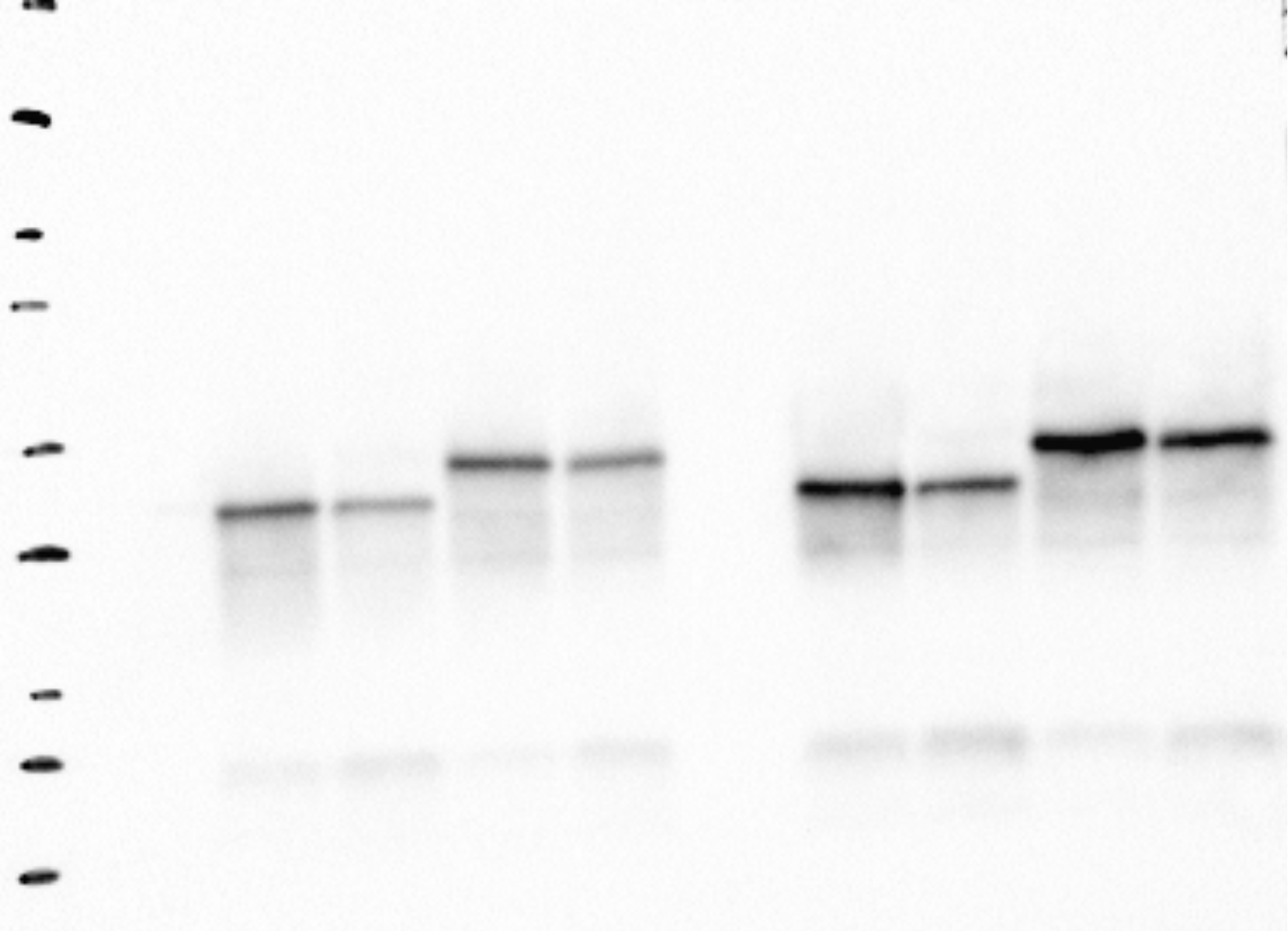

Supplement: Figure 5—source data 1. [file elife-71047-fig5-data1.zip › Figure 5 - Figure Supplement 1E FLAG.tif]

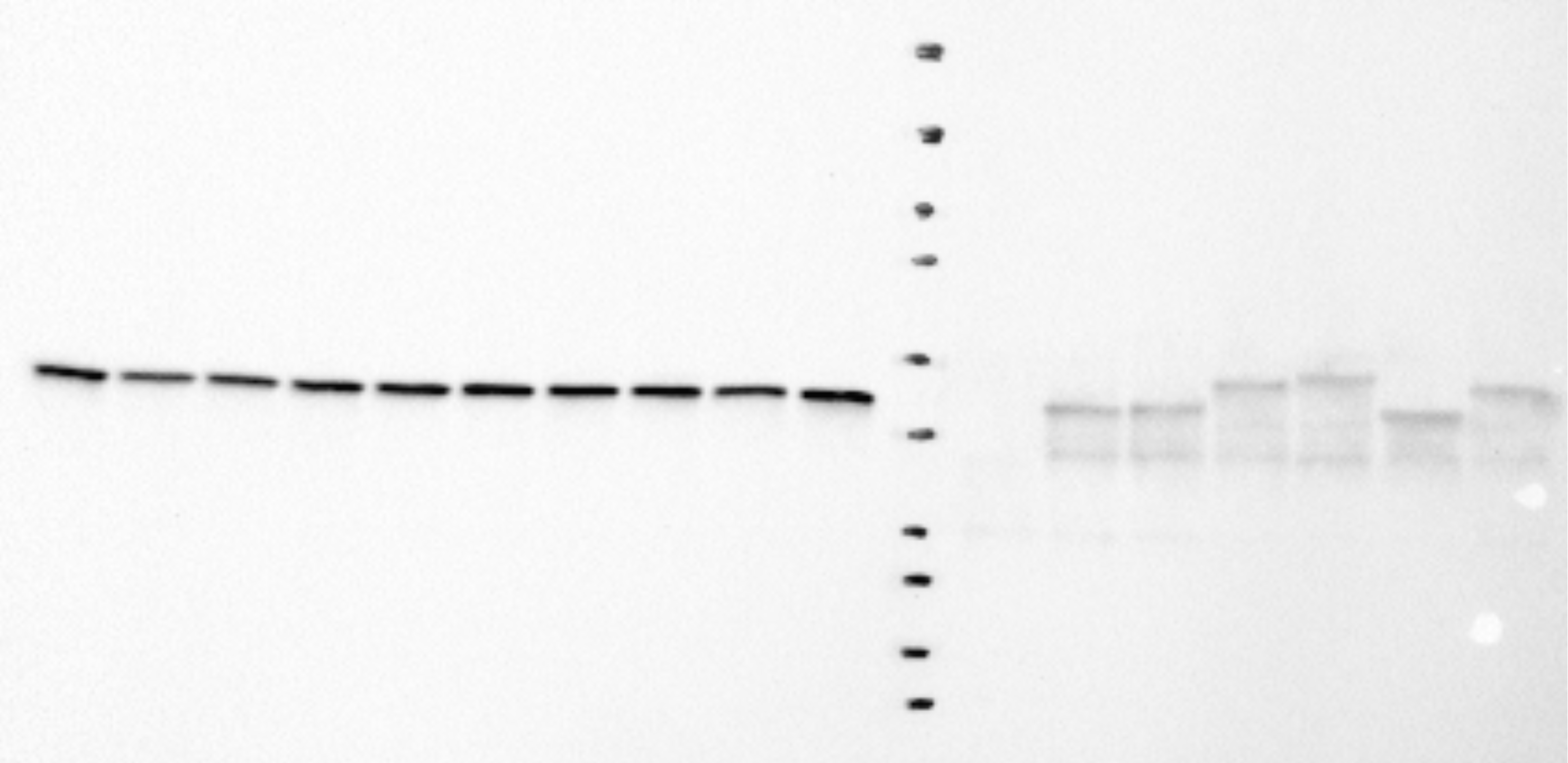

Supplement: Figure 5—source data 1. [file elife-71047-fig5-data1.zip › Figure 5 - Figure Supplement 1J Actin.tif]

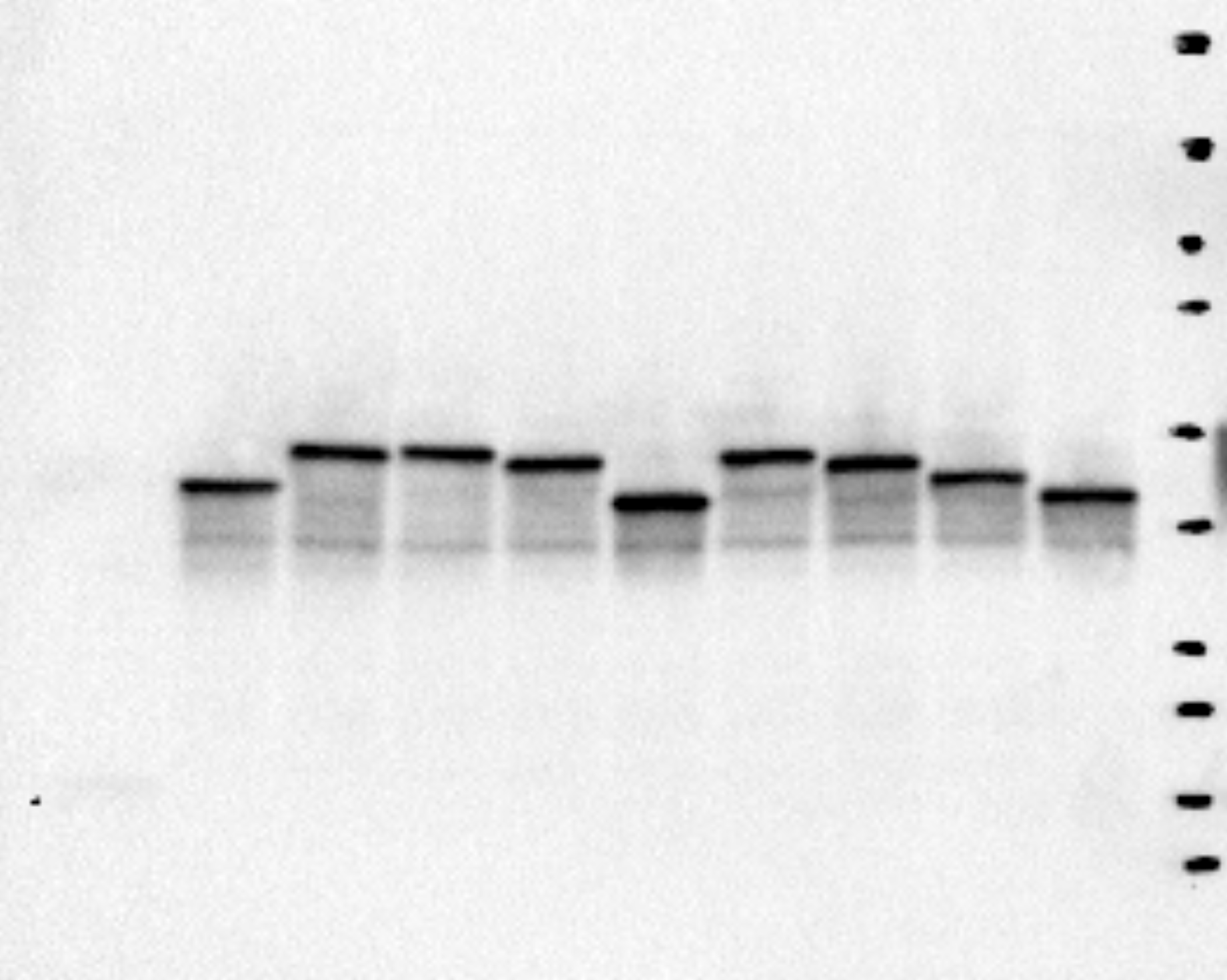

Supplement: Figure 5—source data 1. [file elife-71047-fig5-data1.zip › Figure 5 - Figure Supplement 1J FLAG.tif]

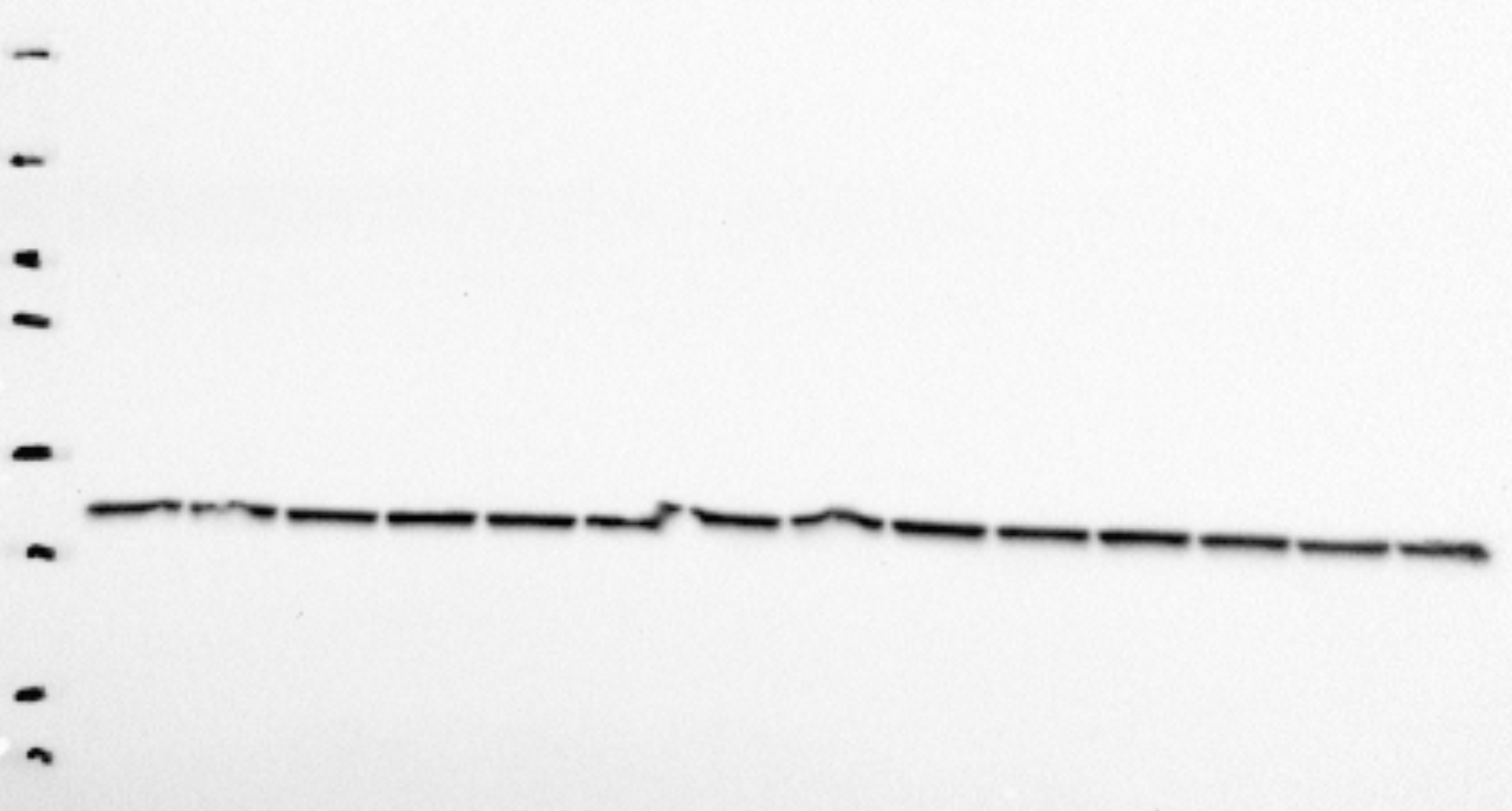

Supplement: Figure 5—source data 1. [file elife-71047-fig5-data1.zip › Figure 5A Actin.tif]

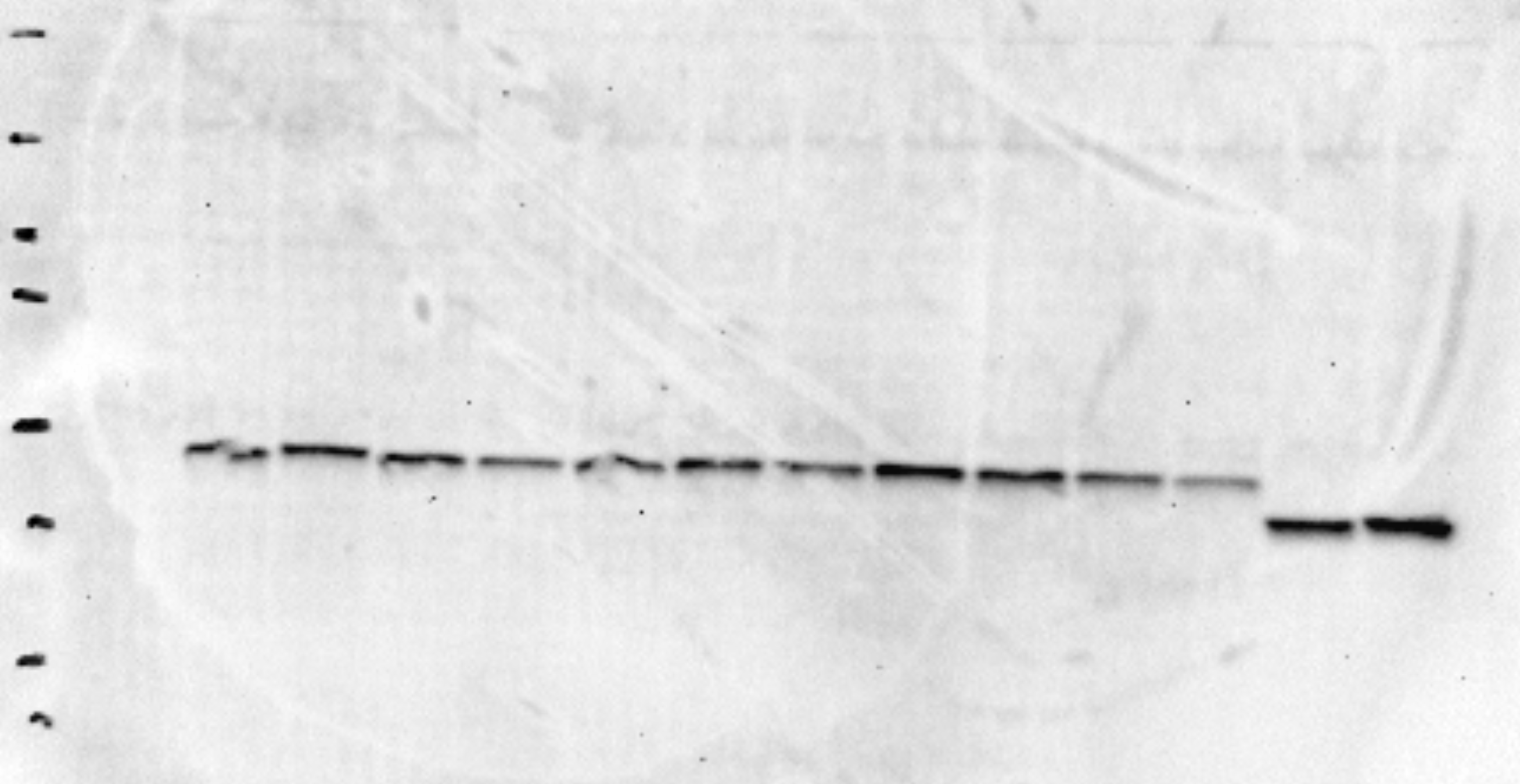

Supplement: Figure 5—source data 1. [file elife-71047-fig5-data1.zip › Figure 5A OAS1.tif]

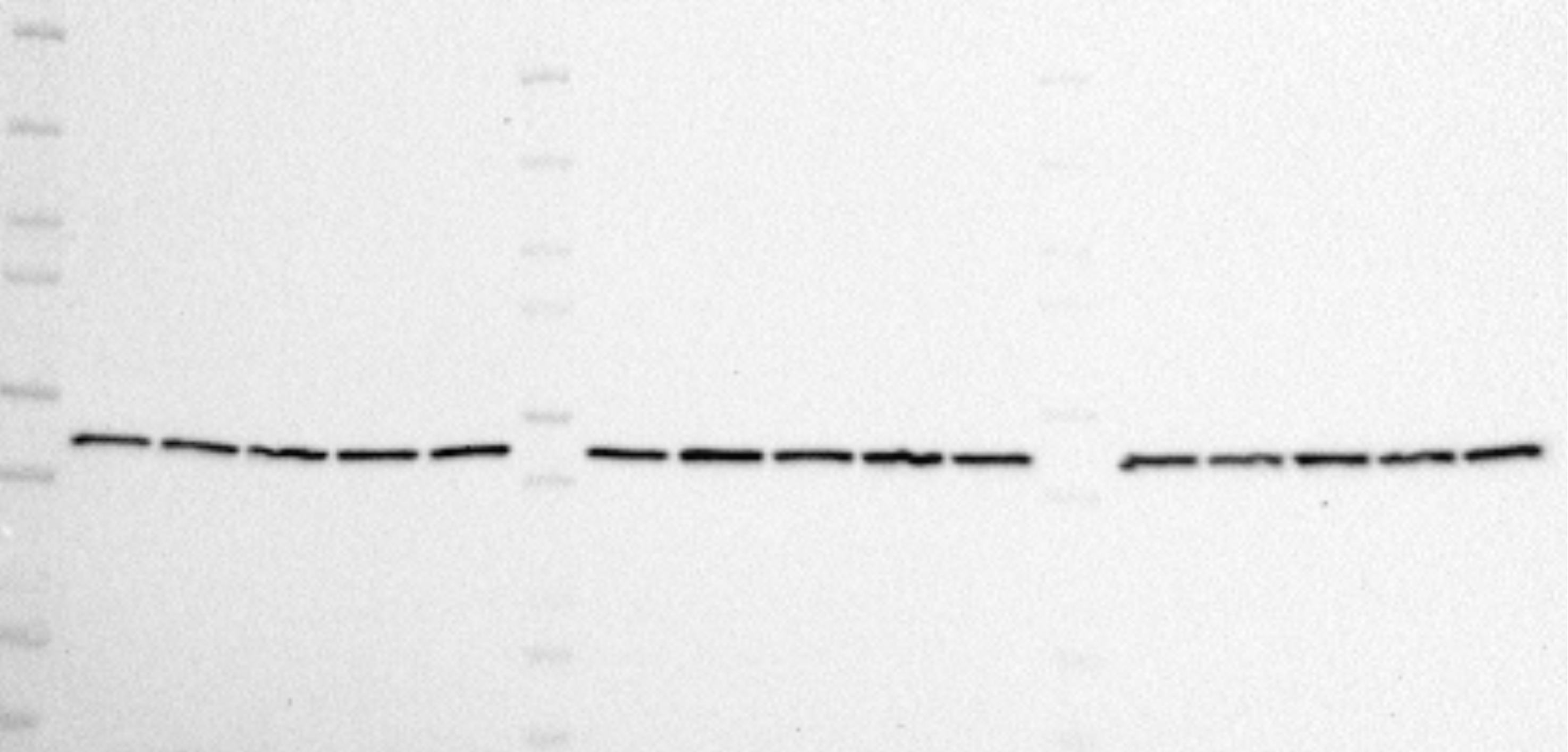

Supplement: Figure 5—source data 1. [file elife-71047-fig5-data1.zip › Figure 5D Actin.tif]

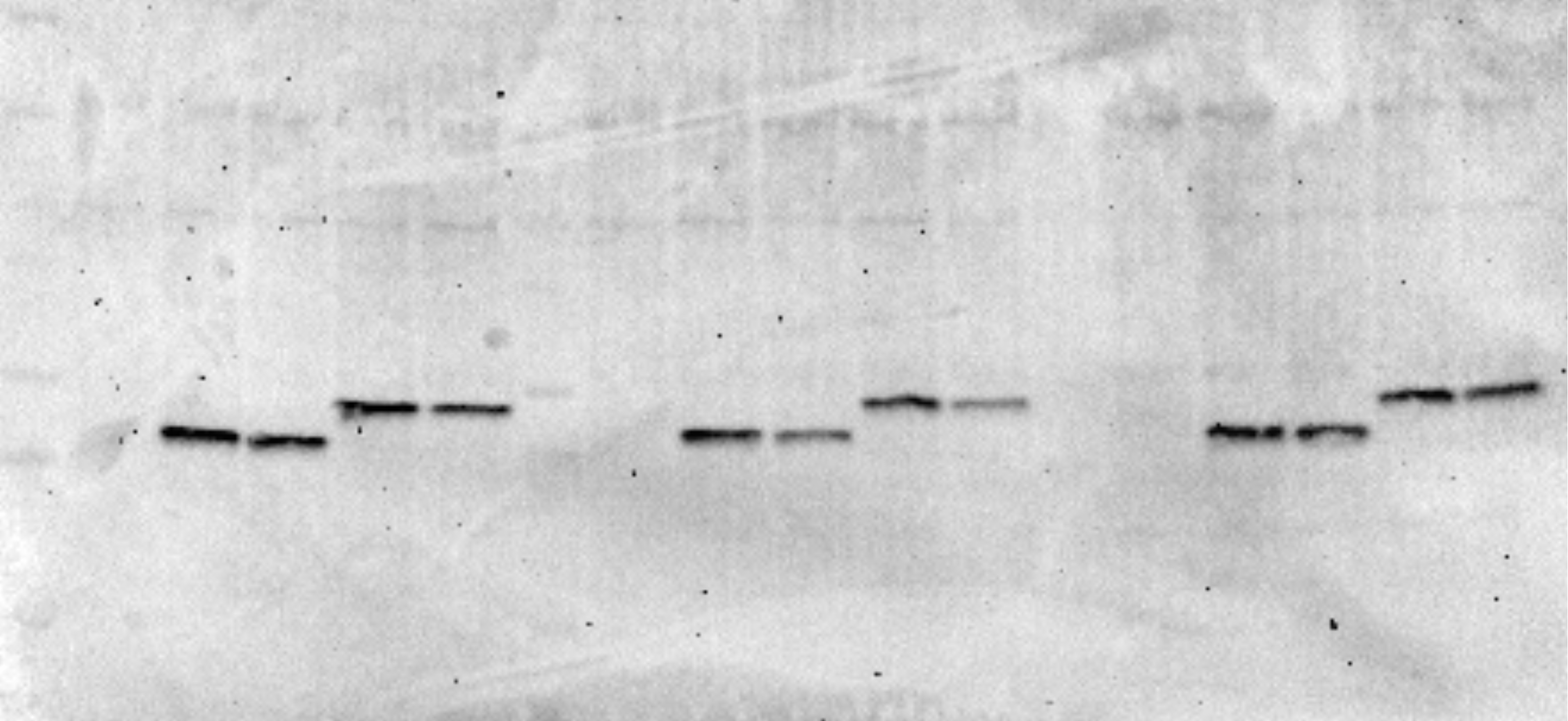

Supplement: Figure 5—source data 1. [file elife-71047-fig5-data1.zip › Figure 5D OAS1.tif]

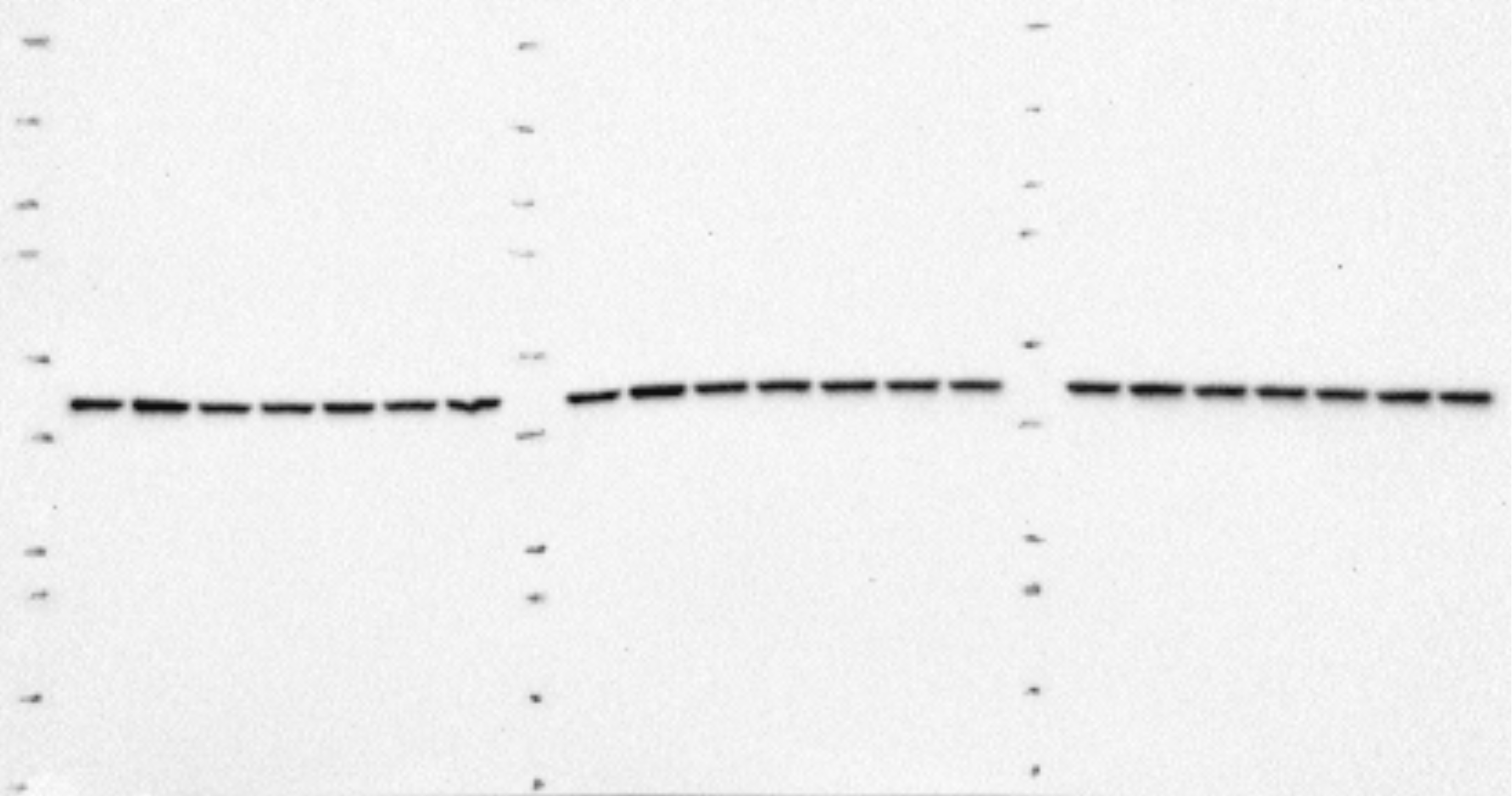

Supplement: Figure 5—source data 1. [file elife-71047-fig5-data1.zip › Figure 5G Actin.tif]

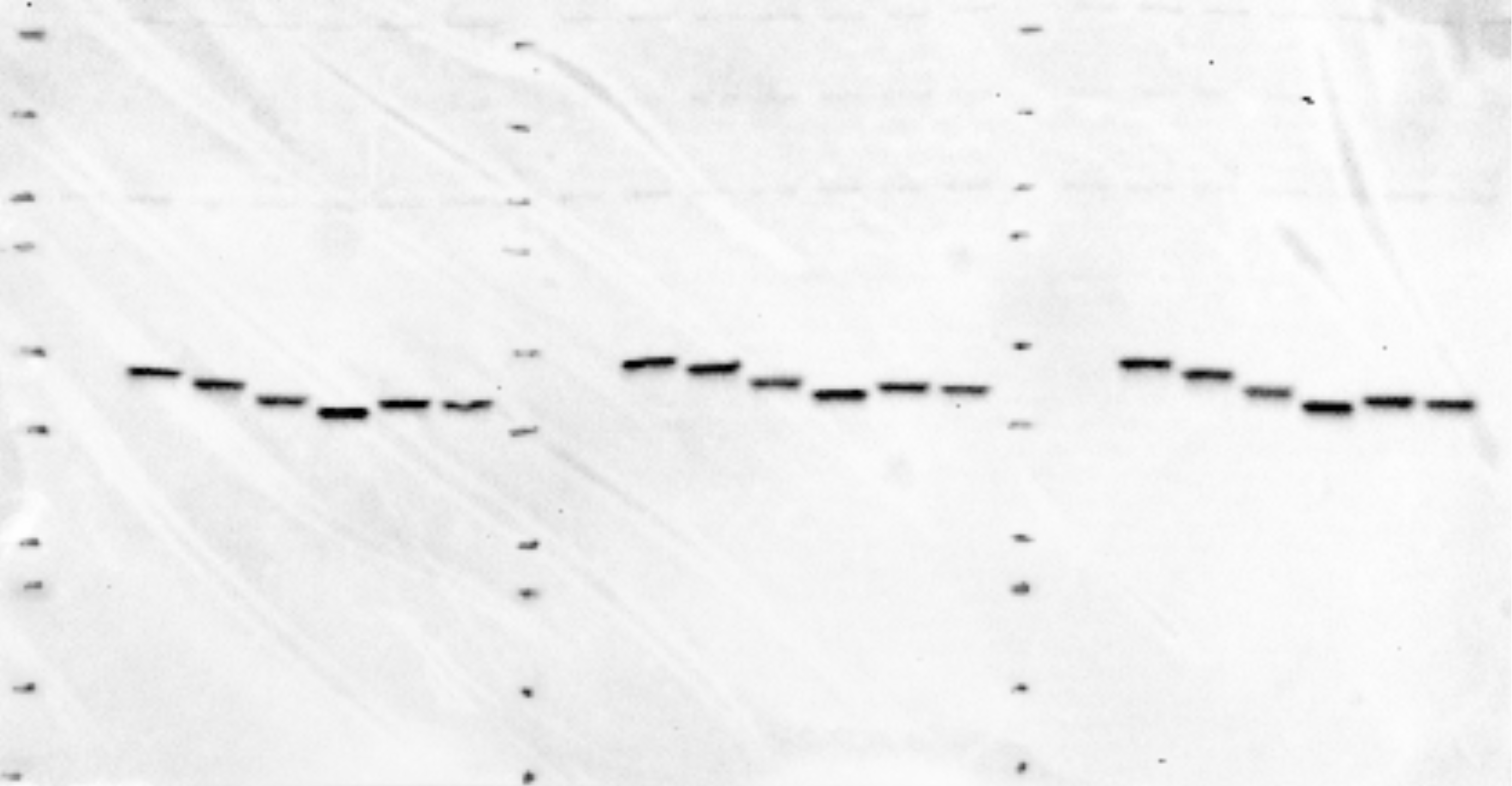

Supplement: Figure 5—source data 1. [file elife-71047-fig5-data1.zip › Figure 5G OAS1.tif]
