# Supplementary material for: Endomembrane targeting of human OAS1 p46 augments antiviral activity
Source: eLife. 2021 Aug 3;10:e71047. doi: 10.7554/eLife.71047 (PMC8357416; doi:10.7554/eLife.71047)

**Figure 1B)**

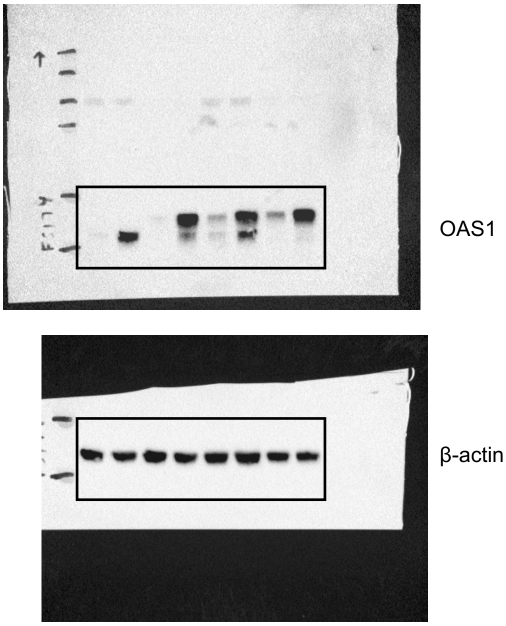


**Figure 1D)**

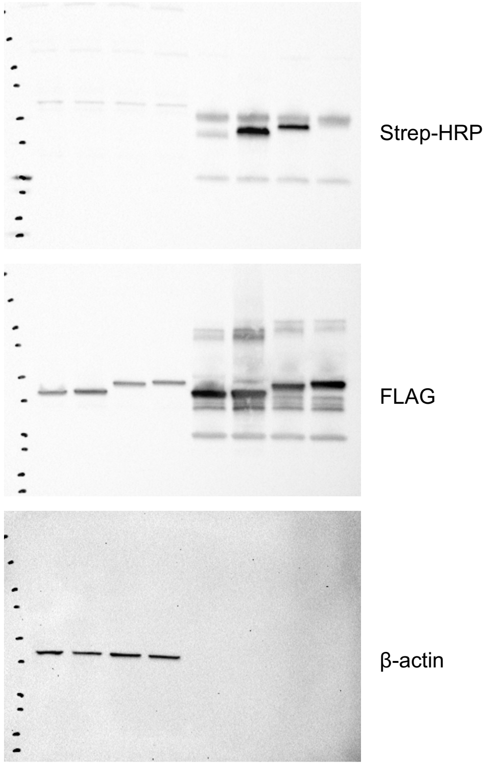


**Figure 1C)**
**left**

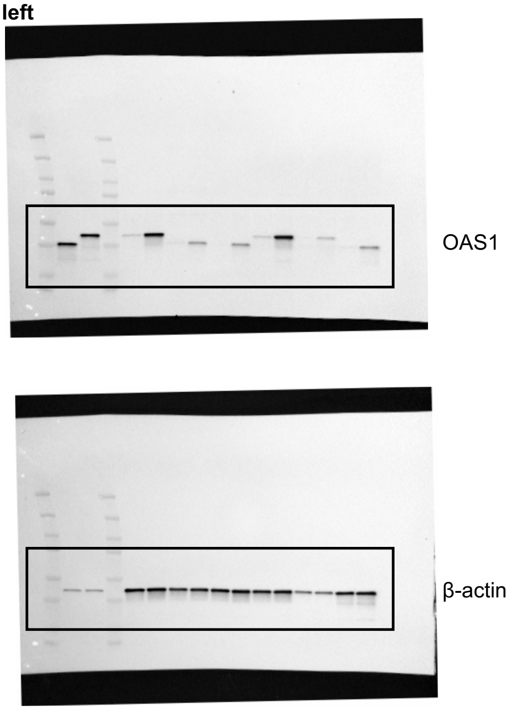


**Figure 1C)**
**right**

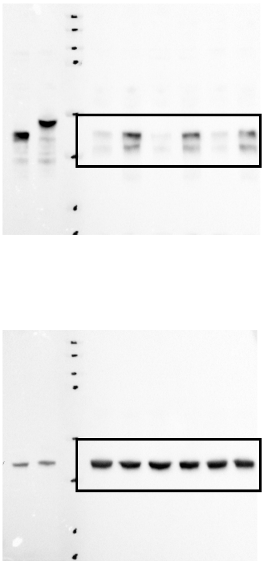

**Figure 2A)**

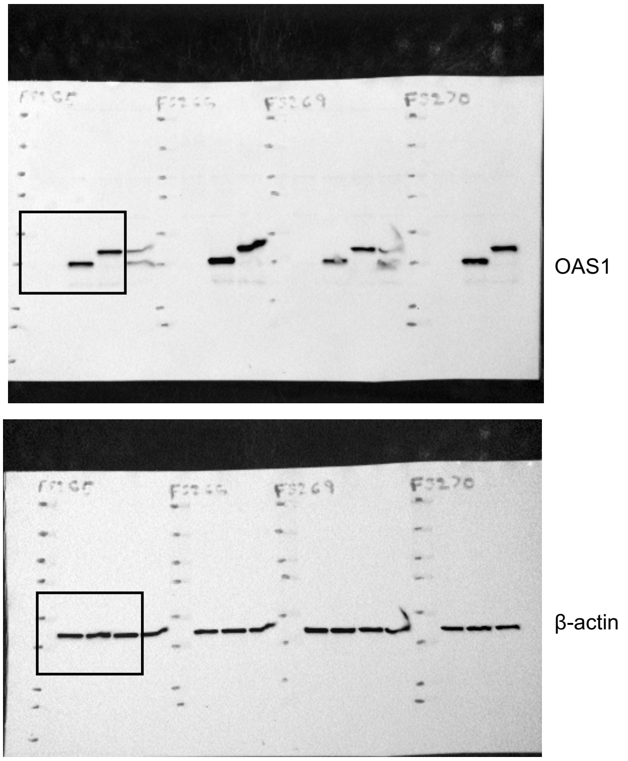


**Figure 2D)**

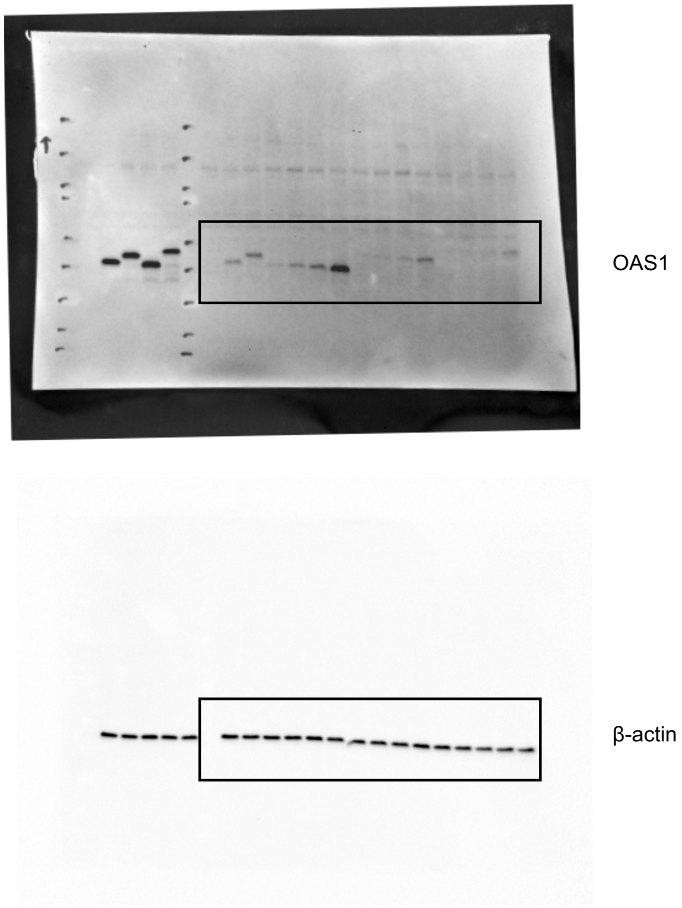


**Figure 2F)**

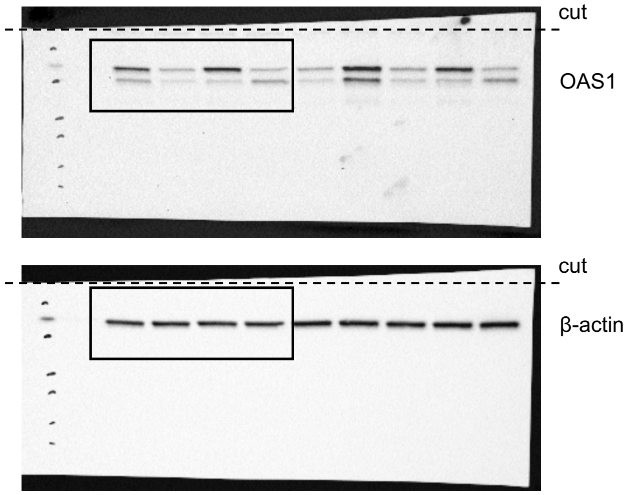

**Figure 3A)**

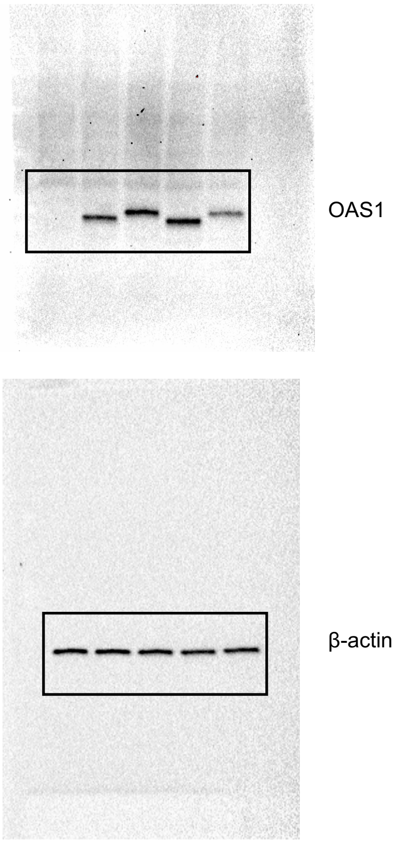


**Figure 3D)**

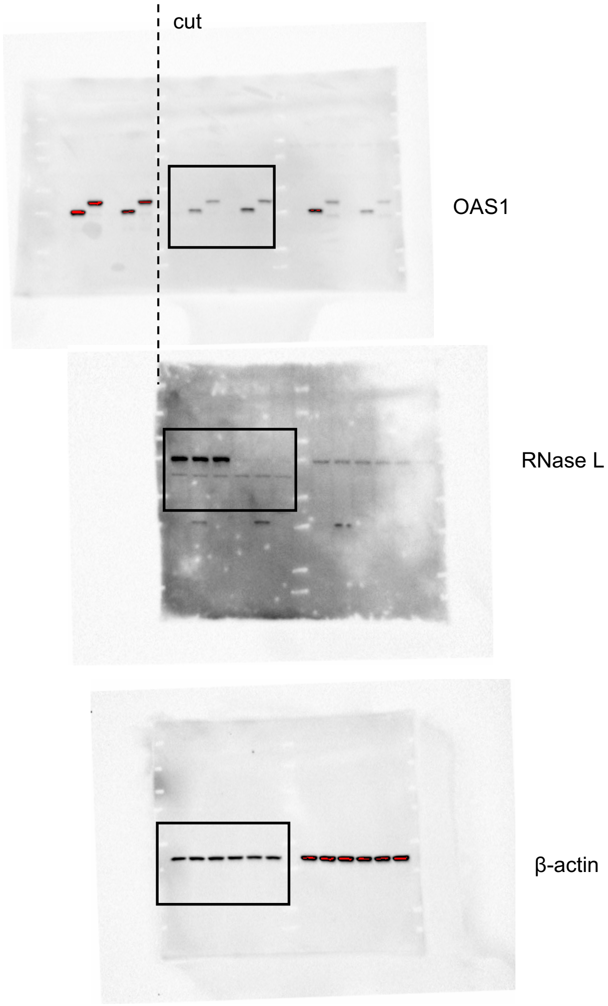


**Figure 3G)**

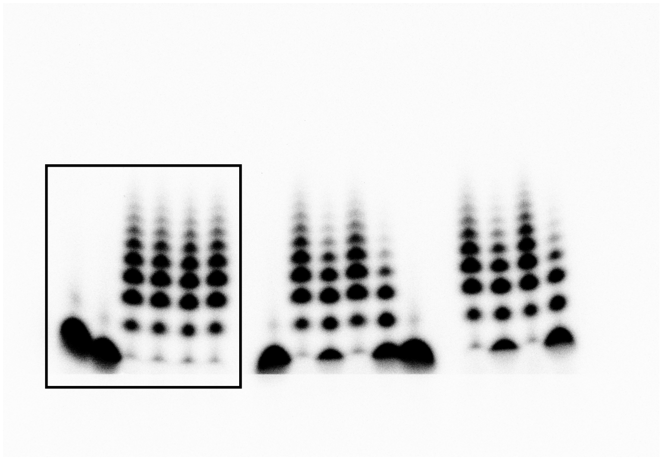

**Figure 3H)**

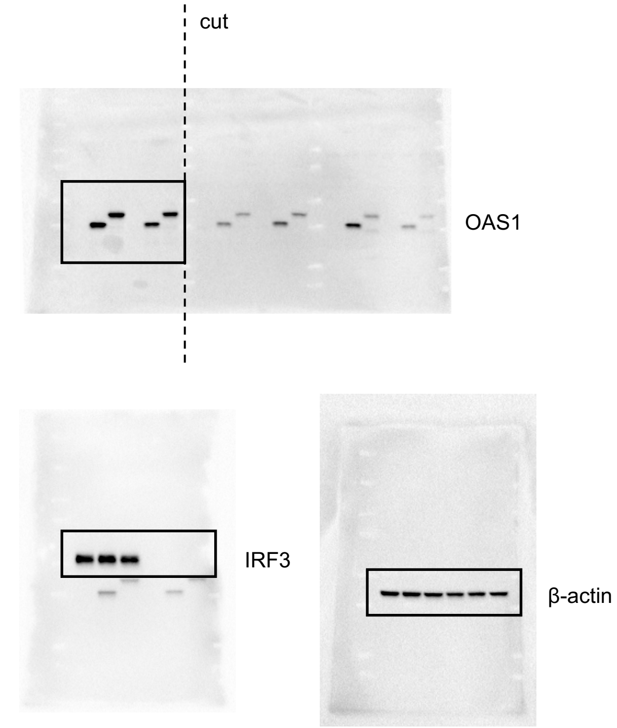

**Figure 4B)**

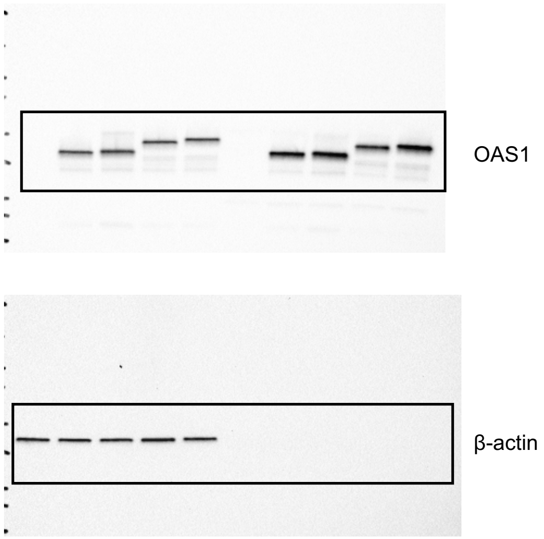


**Figure 4C)**

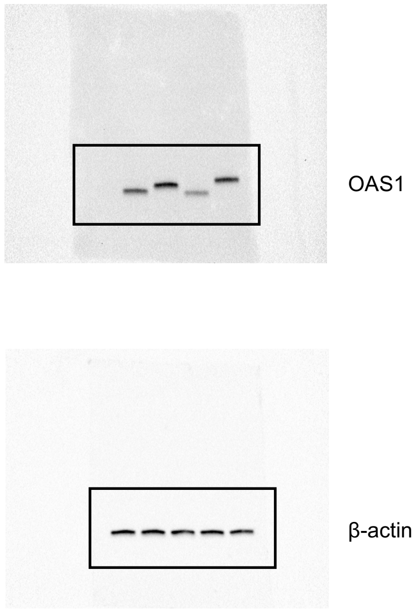


**Figure 4G)**

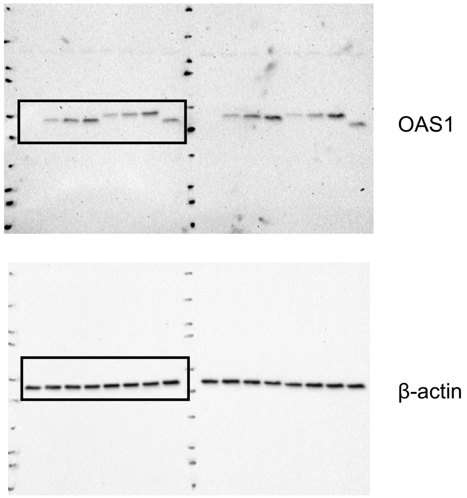

**Figure 5B)**

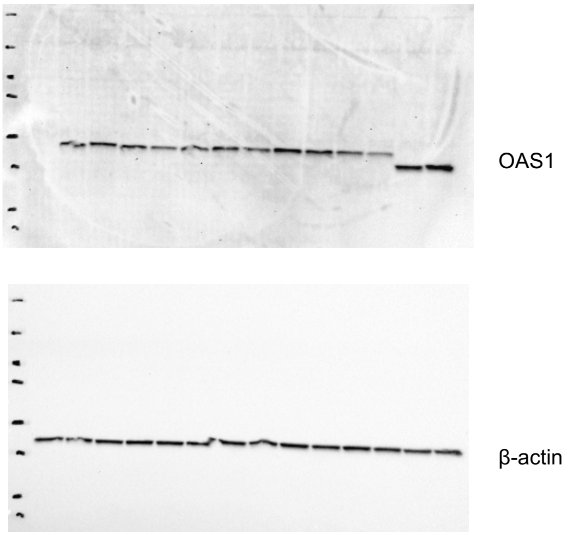


**Figure 5D)**

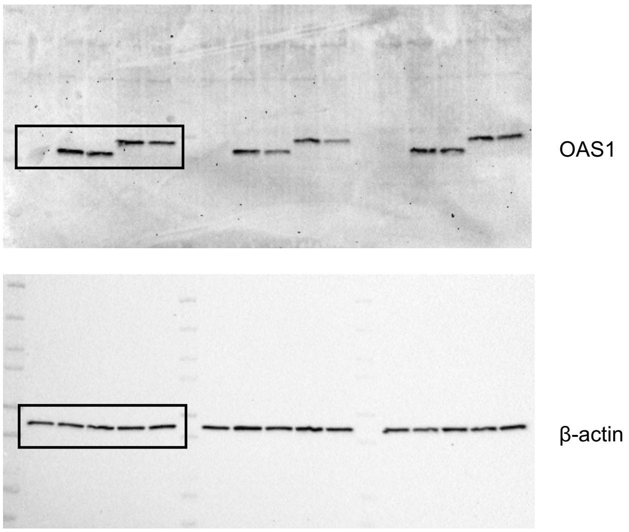


**Figure 5G)**

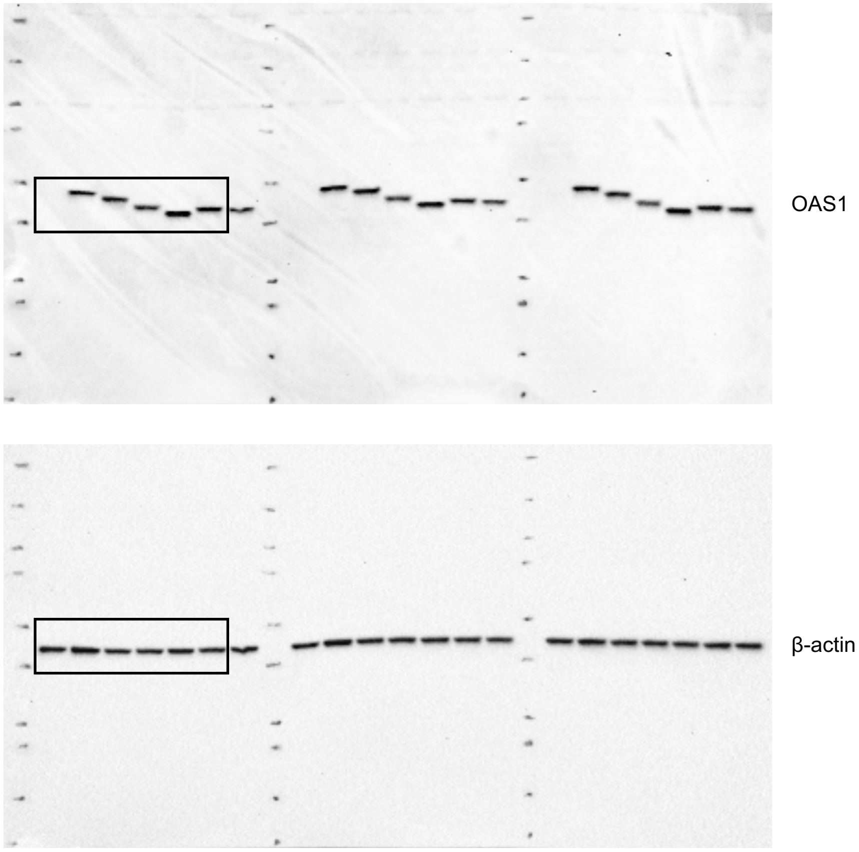

**Figure 6G)**

Hardy Weinberg equilibrium

| CHR | SNP | TEST | A1 | A2 | GENO | O(HET) | E(HET) | P |
|---|---|---|---|---|---|---|---|---|
| 12 | rs10774671 | ALL | G | A | 17/58/58 | 0.4361 | 0.4525 | 0.7023 |
| 12 | rs10774671 | AFF | G | A | 4/10/20 | 0.2941 | 0.3893 | 0.1841 |
| 12 | rs10774671 | UNAFF | G | A | 13/48/38 | 0.4848 | 0.4681 | 0.8313 |

Allele frequency

| CHR | SNP | A1 | A2 | MAF | NCHROBS |
|---|---|---|---|---|---|
| 12 | rs10774671 | G | A | 0.3459 | 266 |

Missingness

1 control subject with missing genotype

Association

Logistic regression dominant model ref allele G, adjusted for covariates

| CHR | SNP | BP | A1 | TEST | NMISS | OR | SE | L95 | U95 | STAT | P |
|---|---|---|---|---|---|---|---|---|---|---|---|
| 12 | rs10774671 | 1.13E+08 | G | DOM | 131 | 0.3506 | 0.4402 | 0.1479 | 0.8308 | -2.381 | 0.01727 |
| 12 | rs10774671 | 1.13E+08 | G | Sex | 131 | 1.217 | 0.4345 | 0.5193 | 2.852 | 0.4521 | 0.6512 |
| 12 | rs10774671 | 1.13E+08 | G | RaceEthnic | 131 | 1.28 | 0.133 | 0.9862 | 1.661 | 1.855 | 0.06355 |

**Figure 1 – Figure Supplement 2B)**

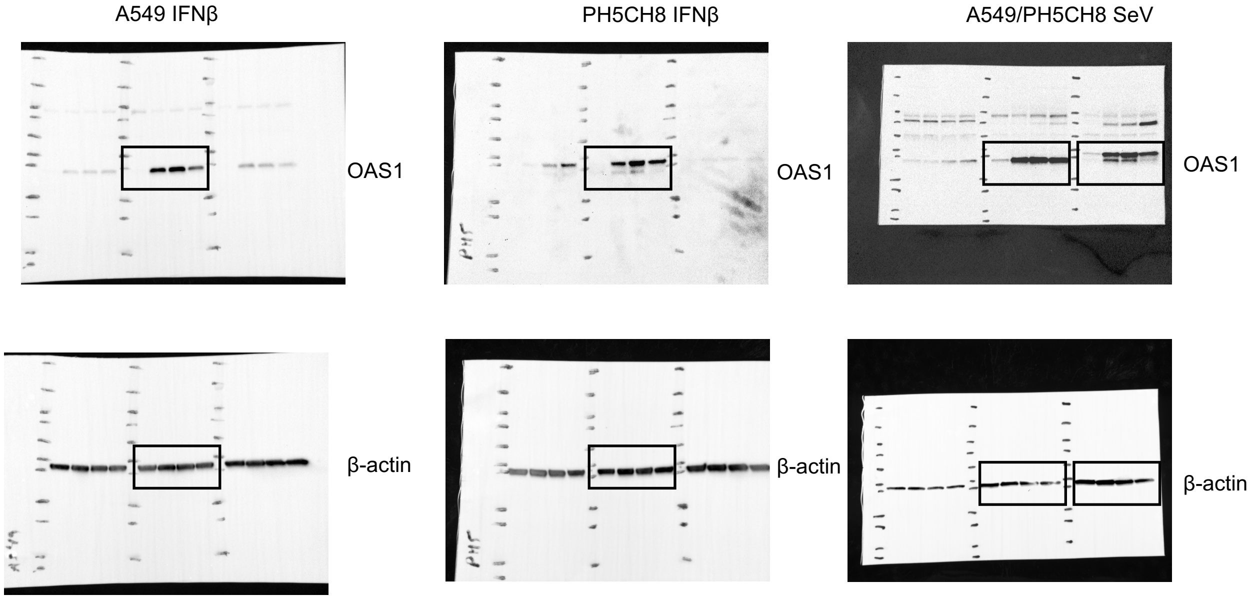


**Figure 1 – Figure Supplement 2C)**

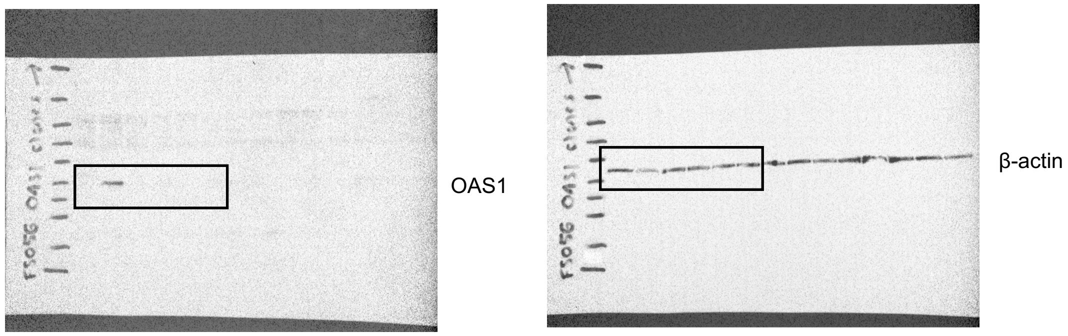


**Figure 1 – Figure Supplement 2F)**

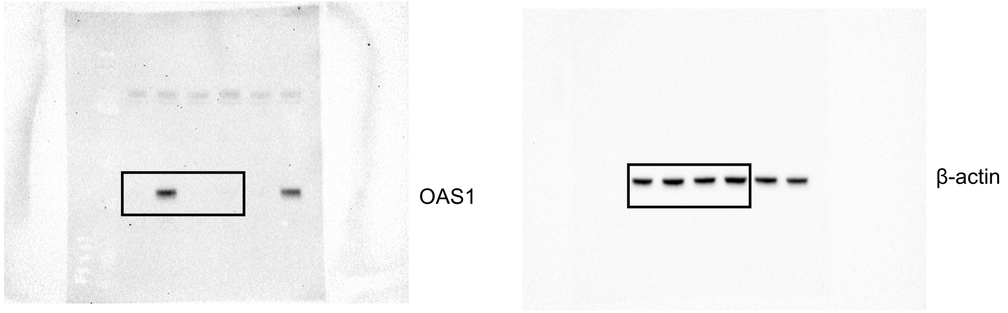


**Figure 1 – Figure Supplement 2G)**

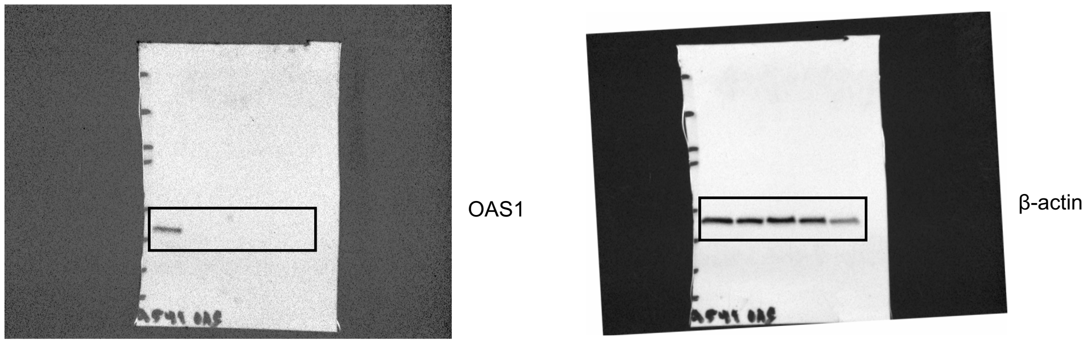

**Figure 3 – Figure Supplement 1A)**

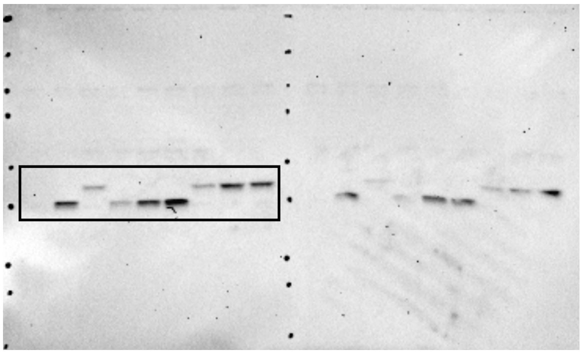

OAS1

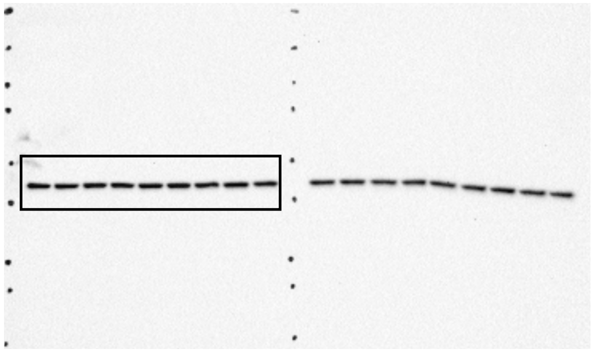

β-actin

**Figure 3 – Figure Supplement 1C)**

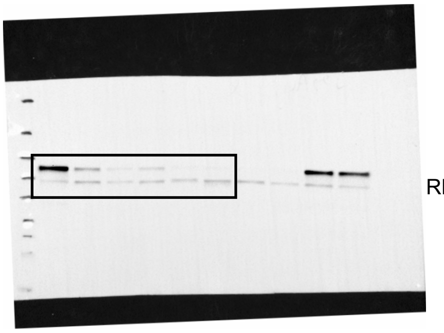

RNase L

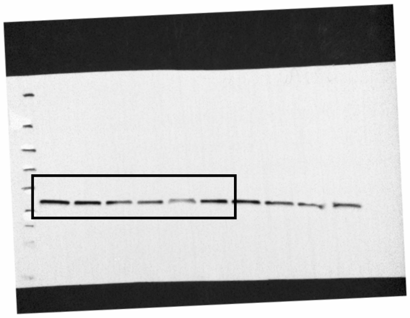

β-actin

**Figure 3 – Figure Supplement 1D)**

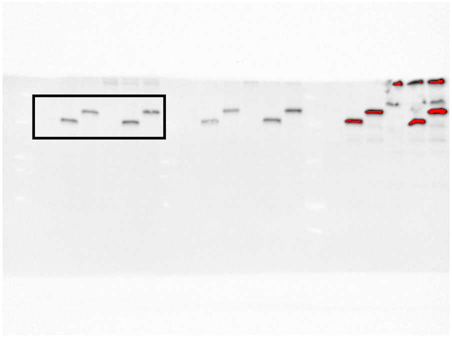

OAS1

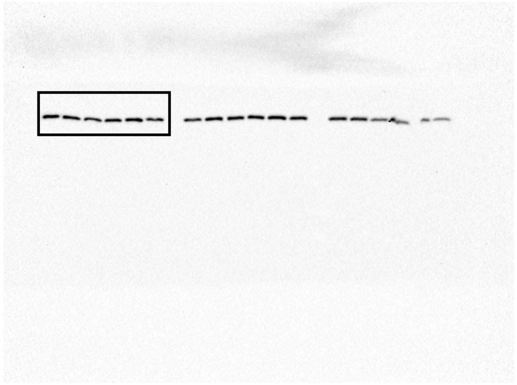

β-actin

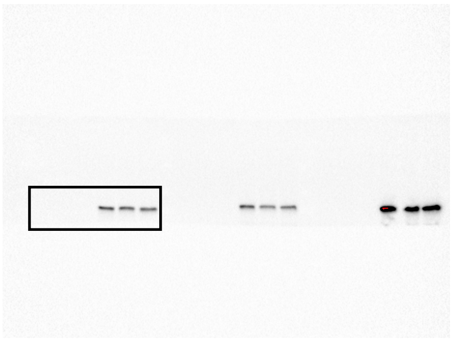

RNase L

**Figure 4 – Figure Supplement 1A)**

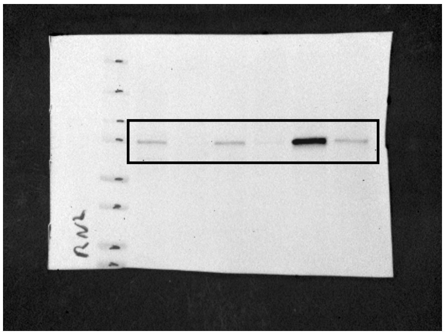

RNase L

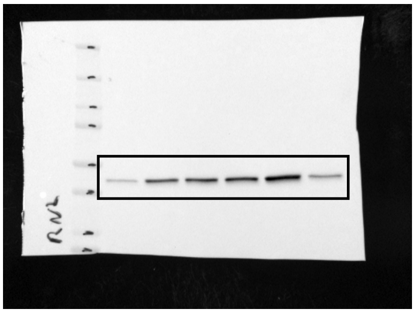

β-actin

**Figure 5 – Figure Supplement 1D)**

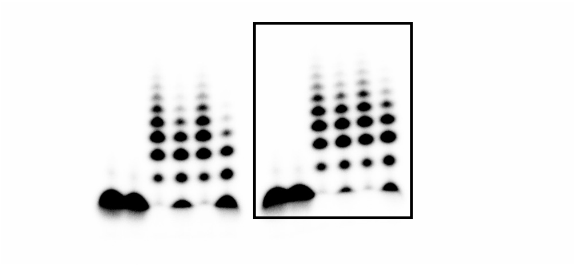

**Figure 5 – Figure Supplement 1E)**

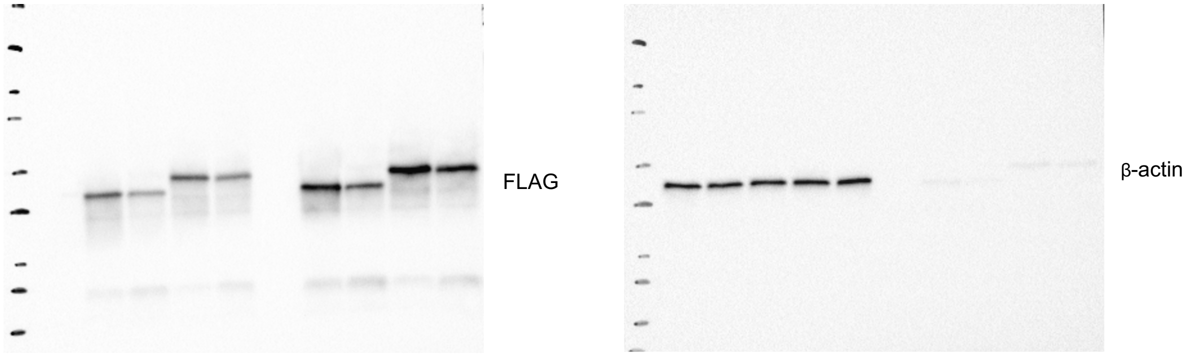


**Figure 5 – Figure Supplement 1J)**

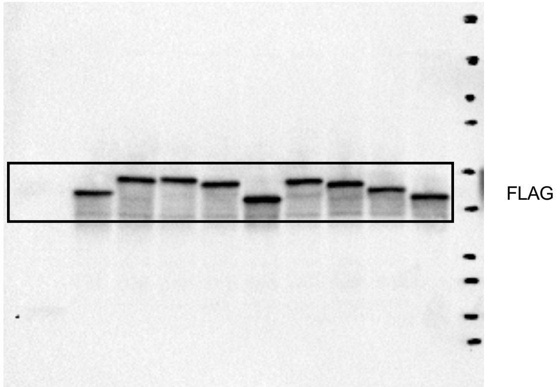


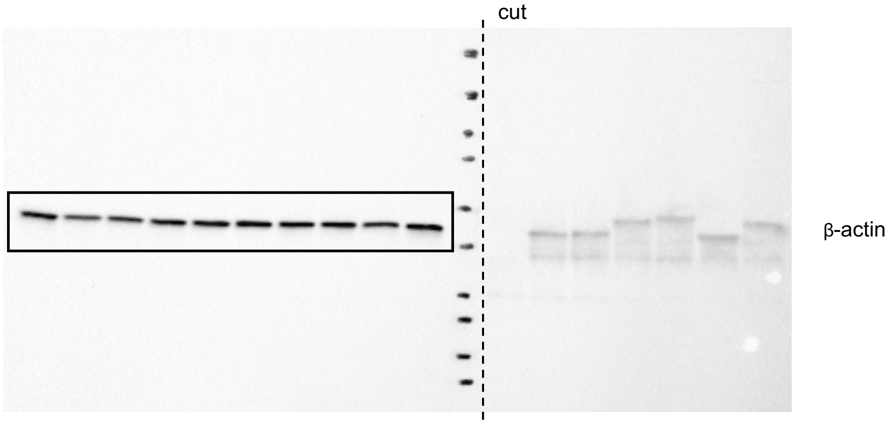

Supplement: Source data 1. [file elife-71047-data1.pdf]
