# Supplementary material for: Endomembrane targeting of human OAS1 p46 augments antiviral activity
Source: eLife. 2021 Aug 3;10:e71047. doi: 10.7554/eLife.71047 (PMC8357416; doi:10.7554/eLife.71047)
Supplement: Supplementary file 1. [file elife-71047-supp1.docx]

**Supplementary file 1. Cells used in this study.**

| **Name** | **Vendor/Source** |
| --- | --- |
| *OAS1* KO HEK293T | Daniel Stetson, UW |
| *RNASEL* KO HEK293T | Daniel Stetson, UW |
| *OAS1* KO Huh7 | This manuscript |
| *OAS1* KO A549 | This manuscript |
| ACE2 HEK293T | This manuscript |
| *IRF3* KO HEK293FT | Daniel Stetson, UW |
| HEK293FT | ATCC |
| HEK293T | ATCC |
| A549 | ATCC |
| PH5CH8 | Michael Gale, Jr., UW |
| THP-1 | ATCC |
| Daudi | Saumendra Sarkar, UPitt/ATCC |
| MDCK | Michael Gale, Jr., UW |
| Huh7 | Michael Gale, Jr., UW |
| Vero WHO | Michael Gale, Jr., UW |
| Vero R6 | Ralph Baric, UNC |
| PBMCs | Karen Cerosaletti, BRI |
| Human primary fibroblasts | Eric Allenspach, SCRI |
