# Supplementary material for: Endomembrane targeting of human OAS1 p46 augments antiviral activity
Source: eLife. 2021 Aug 3;10:e71047. doi: 10.7554/eLife.71047 (PMC8357416; doi:10.7554/eLife.71047)
Supplement: Supplementary file 2. [file elife-71047-supp2.docx]

**Supplementary file 2. Antibodies used in this study.**

| **Name** | **Vendor/Source** | **Catalog** | **RRID** |
| --- | --- | --- | --- |
| OAS1 (D1W3A) Rabbit mAb | Cell Signaling Technology | 14498 | AB_2798498 |
| RNase L (D4B4J) Rabbit mAb | Cell Signaling Technology | 27281 | AB_2798941 |
| Golgin-97 (CDF4) Mouse mAb | Cell Signaling Technology | 97537 | AB_2800280 |
| Monoclonal Anti-PDIA3 antibody raised in mouse | Sigma-Aldrich | AMAB90988 | AB_2665750 |
| Golgin-97 (CDF4), Unconjugated, Species Reactivity: Human, Host: Mouse / IgG1 | Thermo Scientific | A-21270 | AB_221447 |
| Monoclonal ANTI-FLAG® M2 antibody raised in mouse | Sigma-Aldrich | F3165 | AB_259529 |
| J2 monoclonal antibody (mAb) anti dsRNA, mouse, IgG2a | Scicons | 10010200 | AB_2651015 |
| Mouse monoclonal antibody 9D5 anti dsRNA | Adam Geballe lab | N/A | N/A |
| β-Actin (13E5) Rabbit mAb | Cell Signaling Technology | 4970 | AB_2223172 |
| Goat anti Mouse IgG2a Alexa Fluor 647 | Thermo Fisher Scientific | A21241 | AB_141698 |
| Goat anti Mouse IgG1 Alexa Fluor 594 | Thermo Fisher Scientific | A21125 | AB_2535767 |
| Goat anti Rabbit Alexa Fluor 488 | Thermo Fisher Scientific | A11008 | AB_143165 |
