# Supplementary material for: Endomembrane targeting of human OAS1 p46 augments antiviral activity
Source: eLife. 2021 Aug 3;10:e71047. doi: 10.7554/eLife.71047 (PMC8357416; doi:10.7554/eLife.71047)
Supplement: Supplementary file 3. [file elife-71047-supp3.docx]

**Supplementary file 3: Nucleic acids used in this study.**

| **Primers** | **Sequence 5'-3'** |
| --- | --- |
| OAS1 common For | GCTTGGTACCGAGCTCGGATCC |
| OAS1 common Rev | CAGCAGAATCCAGGAGCTCACTGG |
| OAS1 p42 For | AGTGAGCTCCTGGATTCTGCTGGTGAGACCTCCTGCTTCCTCCC |
| OAS1 p42 wt Rev | CTATAGAATAGGGCCCTCTAGATCAAGCTTCATGGAGAGGGGCAG |
| OAS1 p42 CaaX Rev | TATAGAATAGGGCCCTCTAGATCAGAGGATGGTGCAAGCTTCAT |
| OAS1 p46 For | CCCAGTGAGCTCCTGGATTCTGCTGGCTGAAAGCAACAGTGCAGACG |
| OAS1 p46 wt CaaX Rev | ACTATAGAATAGGGCCCTCTAGATCAGAGGATGGTGCAGGTCCA |
| OAS1 p46 mut CaaX Rev | ACTATAGAATAGGGCCCTCTAGATCAGAGGATGGTGGCGGTC |
| OAS1 p48 For | AGTGAGCTCCTGGATTCTGCTGACCCAGCACACTCCAGGCAG |
| OAS1 p46 C-term mut_F | TAAGAATTGGGATGGGTCCCCAG |
| OAS1 p46 C-term mut_R | GACACTATAGAATAGGGCCCTCTAGA |
| OAS1 p48 Rev | CTATAGAATAGGGCCCTCTAGATCAGGAGACCTGGGTTCTGTCC |
| OAS1DADA_F | GAAGACAACCAGGGCAGCGGCAGATCGGCCTCT |
| OAS1DADA_R | AGAGGCCGATCTGCCGCTGCCCTGGTT |
| OAS1FLAG_F | CGACTCACTATAGGGAGACCCAAGCTTGGTACCGAGCTCGATGGACTACAAAGAC |
| OAS1FLAG_R | GTCCAGAGATTTGGCTGGGGTATTTCTGAGATCCATCATGCTTGTCATCGTCATCCTTGTAATCGATG |
| EMCV_F | AGATCCGGATTGCCAGTCT |
| EMCV_R | CACTATCGTAGCCTTCACGTTG |
| EMCV_probe | /56-FAM/AT ATC GCA G/ZEN/G CTG GGT CCG T/3IABkFQ/ |
| IAV NP Fwd | CGTTCTCCATCAGTCTCCATC |
| IAV NP Rev | GAGTGACATCAAAATCATGGCG |
| OAS1 CFK mut SDM fwd | CTGAGGCCTGGCTGAATTACCCAGCCGCTGCGAATTGGGATGGGTCCCCAGTGA |
| OAS1 CFK mut SDM rev | TCACTGGGGACCCATCCCAATTCGCAGCGGCTGGGTAATTCAGCCAGGCCTCAG |
| OAS1 p46Δ32 SDM fwd | ACGATCCCAGGAGGTATCAGAAATGCACCATCCTC |
| OAS1 p46Δ32 SDM rev | GAGGATGGTGCATTTCTGATACCTCCTGGGATCGT |
| OAS1 p46Δ22 SDM fwd | ACATTGGAACACATGAGTACCCTTGCACCATCCTCT |
| OAS1 p46Δ22 SDM rev | AGAGGATGGTGCAAGGGTACTCATGTGTTCCAATGT |
| OAS1 p46Δ12 SDM fwd | CCCAGCACACTCCAGTGCACCATCCTCTGA |
| OAS1 p46Δ12 SDM rev | TCAGAGGATGGTGCACTGGAGTGTGCTGGG |
| OAS1 SDM common+CTIL fwd | AGAGGGGCAGGGATGAATGGCAGGGAGGACTAAAGAATCGTGCACAGCAGAATCCAGGAGCTCACTGGGGACC |
| OAS1 SDM common+CTIL rev | GGTCCCCAGTGAGCTCCTGGATTCTGCTGTGCACGATTCTTTAGTCCTCCCTGCCATTCATCCCTGCCCCTCT |
| OAS1 p46 Ala mut9 SDM fwd | CAGAGGATGGTGCAGGCCGCGTCCTCTTCTGCCTGT |
| OAS1 p46 Ala mut9 SDM rev | ACAGGCAGAAGAGGACGCGGCCTGCACCATCCTCTG |
| p52_F | CCCAGTGAGCTCCTGGATTCTGCTGCTGAAAGCAACAGTACAGACGATG |
| p52_R | ACTATAGAATAGGGCCCTCTAGATTATCTATGATAGGATAGAGGGCATAGAA |
|  |  |
| **S/AS oligos** | **Sequence 5'-3'** |
| OAS1 p42 WT S | GTGAGACCTCCTGCTTCCTCCCTGCCATTCATCCCTGCCCCTCTCCATGAAGCTTGA |
| OAS1 p42 WT AS | TCAAGCTTCATGGAGAGGGGCAGGGATGAATGGCAGGGAGGAAGCAGGAGGTCTCAC |
| OAS1 p42 CaaX S | GTGAGACCTCCTGCTTCCTCCCTGCCATTCATCCCTGCCCCTCTCCATGAAGCTTGCACCATCCTCTGA |
| OAS1 p42 CaaX AS | TCAGAGGATGGTGCAAGCTTCATGGAGAGGGGCAGGGATGAATGGCAGGGAGGAAGCAGGAGGTCTCAC |
| OAS1 p44 WT S | CCCAGTGAGCTCCTGGATTCTGCTGGTAAACCTCACACTGGTTGGCAGAAGGAACTATACCAATAATTAGTCTAGAGGGCCCTATTCTATAGTGT |
| OAS1 p44 WT AS | ACACTATAGAATAGGGCCCTCTAGACTAATTATTGGTATAGTTCCTTCTGCCAACCAGTGTGAGGTTTACCAGCAGAATCCAGGAGCTCACTGGG |
|  |  |
| **gBlocks (partial)** | **Sequence 5'-3'** |
| OAS1 p46 WT | GCTGAAAGCAACAGTGCAGACGATGAGACCGACGATCCCAGGAGGTATCAGAAATATGGTTACATTGGAACACATGAGTACCCTCATTTCTCTCATAGACCCAGCACACTCCAGGCAGCATCCACCCCACAGGCAGAAGAGGACTGGACCTGCACCATCCTCTGA |
| OAS1 p46 mutCaaX | GCTGAAAGCAACAGTGCAGACGATGAGACCGACGATCCCAGGAGGTATCAGAAATATGGTTACATTGGAACACATGAGTACCCTCATTTCTCTCATAGACCCAGCACACTCCAGGCAGCATCCACCCCACAGGCAGAAGAGGACTGGACCGCCACCATCCTCTGA |
| OAS1 p48 WT | ACCCAGCACACTCCAGGCAGCATCCACCCCACAGGCAGAAGAGGACTGGACCTGCACCATCCTCTGAATGCCAGTGCATCTTGGGGGAAAGGGCTCCAGTGTTATCTGGACCAGTTCCTTCATTTTCAGGTGGGACTCTTGATCCAGAGAGGACAAAGCTCCTCAGTGAGCTGGTGTATAATCCAGGACAGAACCCAGGTCTCCTGA |
| OAS1 p52 WT | CTGAAAGCAACAGTACAGACGATGAGACCGACGATCCCAGGACGTATCAGAAATATGGTTACATTGGAACACATGAGTACCCTCATTTCTCTCATAGACCCAGCACACTCCAGGCAGCATCCACCCCACAGGCAGAAGAGGACTGGACCTGCACCATCCTCTGAATGCCAGTGCATCTTGGGGGAAAGGGCTCCAGTGTTATCTGGACCAGTTCCTTCATTTTCAGGTGGGACTCTTGATCCAGAGAAGACAAAGCTCCTCAGTGAGCTGGTGTATAATCCAGGACAGAACCCAGGTCTCCTGACTCCTGGCCTTCTATGCCCTCTATCCTATCATAGATAA |
| OAS1 p46 Ala mut1 | TGCTTTAAGAATTGGGATGGGTCCCCAGTGAGCTCCTGGATTCTGCTGGCTGCCGCGGCAGCTGCAGACGATGAGACCGACGATCCCAGGAGGTATCAGAAATATGGTTACATTGGAACACATGAGTACCCTCATTTCTCTCATAGACCCAGCACACTCCAGGCAGCATCCACCCCACAGGCAGAAGAGGACTGGACCTGCACCATCCTCTGATCTAGAGGGCCCTATTCTATAGTGTCACCTA |
| OAS1 p46 Ala mut2 | TGCTTTAAGAATTGGGATGGGTCCCCAGTGAGCTCCTGGATTCTGCTGGCTGAAAGCAACAGTGCAGCGGCAGCTGCCGCAGCGCCCAGGAGGTATCAGAAATATGGTTACATTGGAACACATGAGTACCCTCATTTCTCTCATAGACCCAGCACACTCCAGGCAGCATCCACCCCACAGGCAGAAGAGGACTGGACCTGCACCATCCTCTGATCTAGAGGGCCCTATTCTATAGTGTCACCTA |
| OAS1 p46 Ala mut3 | TGCTTTAAGAATTGGGATGGGTCCCCAGTGAGCTCCTGGATTCTGCTGGCTGAAAGCAACAGTGCAGACGATGAGACCGACGATGCTGCCGCGGCAGCTGCATATGGTTACATTGGAACACATGAGTACCCTCATTTCTCTCATAGACCCAGCACACTCCAGGCAGCATCCACCCCACAGGCAGAAGAGGACTGGACCTGCACCATCCTCTGATCTAGAGGGCCCTATTCTATAGTGTCACCTA |
| OAS1 p46 Ala mut4 | TGCTTTAAGAATTGGGATGGGTCCCCAGTGAGCTCCTGGATTCTGCTGGCTGAAAGCAACAGTGCAGACGATGAGACCGACGATCCCAGGAGGTATCAGAAAGCTGCCGCGGCAGCTGCACATGAGTACCCTCATTTCTCTCATAGACCCAGCACACTCCAGGCAGCATCCACCCCACAGGCAGAAGAGGACTGGACCTGCACCATCCTCTGATCTAGAGGGCCCTATTCTATAGTGTCACCTA |
| OAS1 p46 Ala mut5 | TGCTTTAAGAATTGGGATGGGTCCCCAGTGAGCTCCTGGATTCTGCTGGCTGAAAGCAACAGTGCAGACGATGAGACCGACGATCCCAGGAGGTATCAGAAATATGGTTACATTGGAACAGCTGCCGCGGCAGCTGCATCTCATAGACCCAGCACACTCCAGGCAGCATCCACCCCACAGGCAGAAGAGGACTGGACCTGCACCATCCTCTGATCTAGAGGGCCCTATTCTATAGTGTCACCTA |
| OAS1 p46 Ala mut6 | TGCTTTAAGAATTGGGATGGGTCCCCAGTGAGCTCCTGGATTCTGCTGGCTGAAAGCAACAGTGCAGACGATGAGACCGACGATCCCAGGAGGTATCAGAAATATGGTTACATTGGAACACATGAGTACCCTCATTTCGCTGCCGCGGCAGCTGCACTCCAGGCAGCATCCACCCCACAGGCAGAAGAGGACTGGACCTGCACCATCCTCTGATCTAGAGGGCCCTATTCTATAGTGTCACCTA |
| OAS1 p46 Ala mut7 | TGCTTTAAGAATTGGGATGGGTCCCCAGTGAGCTCCTGGATTCTGCTGGCTGAAAGCAACAGTGCAGACGATGAGACCGACGATCCCAGGAGGTATCAGAAATATGGTTACATTGGAACACATGAGTACCCTCATTTCTCTCATAGACCCAGCACAGCTGCCGCGGCAGCTGCACCACAGGCAGAAGAGGACTGGACCTGCACCATCCTCTGATCTAGAGGGCCCTATTCTATAGTGTCACCTA |
| OAS1 p46 Ala mut8 | TGCTTTAAGAATTGGGATGGGTCCCCAGTGAGCTCCTGGATTCTGCTGGCTGAAAGCAACAGTGCAGACGATGAGACCGACGATCCCAGGAGGTATCAGAAATATGGTTACATTGGAACACATGAGTACCCTCATTTCTCTCATAGACCCAGCACACTCCAGGCAGCATCCACCGCTGCCGCGGCAGCTGCATGGACCTGCACCATCCTCTGATCTAGAGGGCCCTATTCTATAGTGTCACCTA |
| OAS1 p46  *Alligator mississippiensis* C-terminus | TGCTTTAAGAATTGGGATGGGTCCCCAGTGAGCTCCTGGATTCTGCTGCCTGAACAAACCCTCAAAGGGAGCAAGGGAGTCTGTGTTCGCTCTGTGGCTAAGCATGAAGCACGAAAACAGAAAGCTGCAGAACTTCAGCCTGTGCTGGTCTCTTCCTACAGCATCCCTGCCTTGGCCCCACAAGAGTTGGAAAAGCAACCCTCCTTTTGCAGCATACTCTGATCTAGAGGGCCCTATTCTATAGTGTCACCTA |
| OAS1 p46  *Bos taurus*  C-terminus | TGCTTTAAGAATTGGGATGGGTCCCCAGTGAGCTCCTGGATTCTGCTGCCCCAAGAACACAGTGACCTGATGTTCCAGGCCTATGATTTTAGACAGCACTGTAGACCCTCTCCAGGAATCCAGTTCCACGGAGGAGCCTCTCCCCAGGTGGAAGAGAACTGGACATGTACCATCCTCTGATCTAGAGGGCCCTATTCTATAGTGTCACCTA |
| OAS1 p46  *Pteropus alecto*  C-terminus | TGCTTTAAGAATTGGGATGGGTCCCCAGTGAGCTCCTGGATTCTGCTGCCCTACGACACACCCCACGTGGAAGAGGACCAGTGGTGTGCCATCCTCTGATCTAGAGGGCCCTATTCTATAGTGTCACCTA |
| OAS1 p46  *Vulpes vulpes*  C-terminus | TGCTTTAAGAATTGGGATGGGTCCCCAGTGAGCTCCTGGATTCTGCTGCTTGAAGAAGACTATGAGGACAATTGGATAACCTCTGAACACAGGACATATTCATACCATGATTATGGTTGGCGCCCTGTATCCTCTGGGAGCCTCAACACAGGCATGACACAGTCCATTCCCCAGCAGGAAGAAAACTGGATGTGTACCATCCTCTGATCTAGAGGGCCCTATTCTATAGTGTCACCTA |
| **siRNA** | **Sequence 5'-3'** |
| OAS1 | AGUCAAGCACUGGUACCAACAAUUUUGGUACCAGUG |
| p42 | GGUCACAAUCGAGGGUUAUUCCAGAAACCCUCGAU |
| p46 | AGAGAGAUUUAGAUAAGAUUCAUUCUCUUAUCUAAA |
