# Supplementary material for: Endomembrane targeting of human OAS1 p46 augments antiviral activity
Source: eLife. 2021 Aug 3;10:e71047. doi: 10.7554/eLife.71047 (PMC8357416; doi:10.7554/eLife.71047)
Supplement: Supplementary file 4. [file elife-71047-supp4.docx]

**Supplementary file 4. Viruses used in this study.**

| **Name** | **Vendor/Source** | **Catalog** |
| --- | --- | --- |
| Encephalomyocarditis virus | ATCC | VR-1762 |
| West Nile virus Texas | Michael Gale, Jr. |  |
| CVB3-Nancy | Raul Andino |  |
| Influenza virus A/PR/8/34 | ATCC |  |
| Influenza A virus A/Udorn/72 H3N2 R38A | Michael Gale, Jr. |  |
| Indiana vesiculovirus (VSV-GFP) | Michael Gale, Jr. |  |
| SARS-CoV-2 strain USA/WA-1/2020 | Michael Gale, Jr. |  |
| ZIKV MR766 | Michael Gale, Jr. |  |
