# Supplementary material for: Endomembrane targeting of human OAS1 p46 augments antiviral activity
Source: eLife. 2021 Aug 3;10:e71047. doi: 10.7554/eLife.71047 (PMC8357416; doi:10.7554/eLife.71047)
Supplement: Supplementary file 5. [file elife-71047-supp5.docx]

# Supplementary file 5. Clinical and demographic information for COVID-19 and matched healthy control cohort.

| **Cohort** | **Number** | **M:F** | **Ancestry**  **(self-reported)** | **rs10774671 genotype** | | | **Disease severity** |
| --- | --- | --- | --- | --- | --- | --- | --- |
|  |  |  |  | **AA** | **AG** | **GG** |  |
| COVID-19 Severe | 34 | 15:19 | 21% Caucasian  29% Latino  15% African American  6% Asian  15% American Indian  15% Other race/declined | 4  9  0  2  3  2 | 3  1  3  0  2  1 | 0  0  2  0  0  2 | Hospitalized, critical care unit, mechanical ventilation, or death |
| Ancestry matched controls | 99 | 47:52 | 34% Caucasian  22% Latino  19% African American  12% Asian  8% American Indian  3% Pacific Islander  2%Other race/declined | 16  10  6  4  1  1  0 | 14  11  9  8  4  1  1 | 4  1  4  0  2  1  1 | Not applicable |
